# Tetrabutylammonium iodide-catalyzed oxidative α-azidation of β-ketocarbonyl compounds using sodium azide

**DOI:** 10.3762/bjoc.20.135

**Published:** 2024-07-05

**Authors:** Christopher Mairhofer, David Naderer, Mario Waser

**Affiliations:** 1 Institute of Organic Chemistry, Johannes Kepler University Linz, Altenbergerstrasse 69, 4040 Linz, Austria

**Keywords:** azidation, nitration, organocatalysis, oxidation, quaternary ammonium iodides

## Abstract

We herein report the oxidative α-azidation of carbonyl compounds by using NaN_3_ in the presence of dibenzoyl peroxide catalyzed by tetrabutylammonium iodide (TBAI). By utilizing these readily available bulk chemicals a variety of cyclic β-ketocarbonyl derivatives can be efficiently α-azidated under operationally simple conditions. Control experiments support a mechanistic scenario involving in situ formation of an ammonium hypoiodite species which first facilitates the α-iodination of the pronucleophile, followed by a phase-transfer-catalyzed nucleophilic substitution by the azide. Furthermore, we also show that an analogous α-nitration by using NaNO_2_ under otherwise identical conditions is possible as well.

## Introduction

**Scheme 1:**
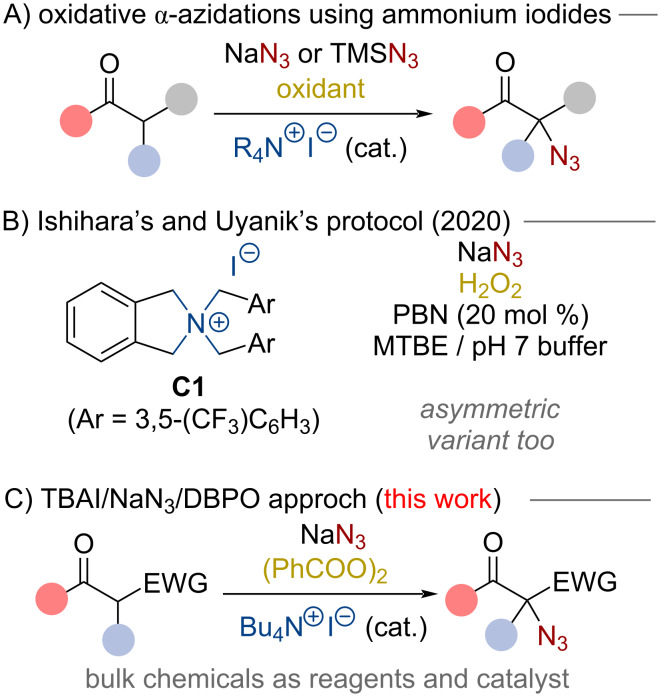
General illustration of the oxidative α-azidation of carbonyl derivatives using quaternary ammonium iodides (A), Ishihara’s protocol using NaN_3_ (B), and the herein reported combination of Bu_4_NI (TBAI), NaN_3_ and DBPO ((PhCOO)_2_) (C).

Organic compounds containing an azide functionality are highly valuable synthesis targets that offer considerable potential for various applications and further manipulations [1–14]. For example, such molecules can be utilized to access free amines [3,13] and undergo Staudinger-type ligations [14]. Furthermore, they can be very efficiently employed for triazole-forming 1,3-dipolar cycloadditions with alkynes (“click-chemistry”) [9–12]. As a consequence, the synthesis of organic azides is an important task and it comes as no surprise that a variety of conceptually complementary strategies to install azide groups in organic molecules have been reported [1–8]. α-Azido carbonyl derivatives are especially interesting targets which can be accessed by different approaches [6–8]. Maybe the most classical way to access organic azides is based on the utilization of pre-functionalized starting materials where a suited leaving group undergoes substitution using nucleophilic azide sources such as NaN_3_ or TMSN_3_ [6–7,15]. In addition, the recent years have seen remarkable progress in utilizing electrophilic azide-transfer reagents, i.e., hypervalent iodine-based compounds, for (asymmetric) α-azidations [16–23]. Besides these valuable approaches, which either require appropriate pre-functionalization of the starting materials (nucleophilic approach), or rely on more advanced N_3_-transfer agents (electrophilic approach), over the course of the last years also α-azidations of enolate-type precursors using nucleophilic azide sources under oxidative conditions have been introduced very successfully [24–31]. Such oxidative coupling strategies of two inherently nucleophilic species allow for the direct utilization of simple starting materials in an efficient manner and especially the use of quaternary ammonium iodides as redox active catalysts has emerged as a powerful catalysis concept for such transformations [32–36]. These oxidative approaches, which usually proceed via the in situ formation of catalytically-competent ammonium hypoiodite species, can normally be carried out under operationally simple conditions, thus allowing for the use of easily accessible starting materials. Our group has a longstanding research interest in α-heterofunctionalization reactions under oxidative conditions [37–39] and we [30], as well as others [28–29,31], have recently also explored the use of simple quaternary ammonium iodides for oxidative α-azidations of carbonyl compounds (Scheme 1A). Hereby different strategies using different quaternary ammonium iodide derivatives and different azide sources were investigated and especially Uyanik’s and Ishihara’s recent approach using NaN_3_ in combination with the carefully designed achiral catalyst **C1** represents a remarkable advancement in this field (Scheme 1B [31]). In contrast to previous oxidative quaternary ammonium iodide catalysis reports [28–30], this method does not require the use of TMSN_3_, thus presenting an efficient oxidative α-azidation protocol utilizing NaN_3_, which arguably represents the most easily available and cheapest nucleophilic N_3_ source (for other remarkable approaches using alternative catalysts and oxidants see references [24–26]). In addition to the racemic approach, they also showed that this reaction can be rendered enantioselective by using advanced Maruoka-type quaternary ammonium iodides [40]. Interestingly, designer catalyst **C1** was found being catalytically superior compared to Bu_4_NI (TBAI) when using H_2_O_2_ as the oxidant. Furthermore, it turned out that addition of PBN (phenyl *N*-*t*ert-butylnitrone) has a beneficial effect on the reaction and that carefully buffered conditions are best-suited. We have recently established the use of dibenzoyl peroxide (DBPO) as a very powerful oxidant for oxidative heterofunctionalization reactions using simple nucleophilic inorganic salts as heteroatom transfer reagents [39,41]. This was successfully demonstrated for the non-catalyzed α-S(e)CN-functionalization of different pronucleophiles [39] as well as the benzylic azidation of alkylphenol derivatives with NaN_3_ using TBAI as a catalyst [41]. Considering the fact that TBAI clearly represents one of the most easily available quaternary ammonium iodides and keeping in mind our successfully demonstrated matching combination of this catalyst with NaN_3_ and DBPO for our benzylic azidations [41], we were thus wondering if the use of these simple bulk chemicals also allows for the oxidative α-azidation of different carbonyl-based pronucleophiles. As outlined in this contribution, this reagent/catalyst system allows indeed for high yielding direct α-azidations of different (cyclic) β-ketocarbonyl derivatives (Scheme 1C), thus resulting in an operationally simple protocol to access α-azidated carbonyl derivatives. In addition, we have also carried out some test reactions using NaNO_2_ instead of NaN_3_ under otherwise identical conditions and obtained a first proof-of-concept for the analogous, to the best of our knowledge so far unprecedented, quaternary ammonium hypoiodite-mediated α-nitration reaction.

## Results and Discussion

We started our investigations by optimizing the α-azidation of the *tert*-butyl-containing β-ketoester **1a** (Table 1 gives an overview of the most significant results obtained hereby). First experiments testing different oxidants in combination with Bu_4_NI (30 mol %) in 1,2-dichloroethane (DCE), a solvent that we found to be well-suited for oxidative α-heterofunctionalizations before [39], showed that DBPO clearly outperforms all the other oxidants tested under these conditions (Table 1, entries 1–5). While H_2_O_2_ gave **2a** in low yield only (Table 1, entry 1), the use of mCPBA (Table 1, entry 3) and *t*-BuOOH (Table 1, entry 4) mainly resulted in the formation of the α-OH-ketoester **4**. On the other hand, oxone performed significantly better (Table 1, entry 2) but was also found to be inferior as compared to DBPO, which allowed for the more or less quantitative “spot-to-spot” formation of **2a** without any noteworthy side-product formation (Table 1, entry 5). Screening different catalyst/DBPO combinations next (Table 1, entries 5–10), showed that the reaction requires a quaternary ammonium halide containing an easily oxidizable counter anion, i.e., iodide or bromide (Table 1, entries 5 and 7). No product formation was observed in the absence of any catalyst (Table 1, entry 6) or in the presence of Bu_4_NHSO_4_ (Table 1, entry 9) and the use of Bu_4_NCl (Table 1, entry 8) was not satisfying either. On the other hand, the beneficial effect of the quaternary ammonium functionality was clearly underscored by employing KI instead of Bu_4_NI (compare Table 1, entries 10 and 5). While Bu_4_NI allowed for the clean and selective formation of **1a**, we observed significant amounts of the α-I-ketoester **3** when using KI instead. Having established the combination of DBPO and Bu_4_NI as the best-suited catalyst/oxidant combination for the α-azidation of **1a** using NaN_3_, we finally optimized stoichiometry and catalyst loading (Table 1, entries 11–15). Hereby we found the use of 1.2 equiv of NaN_3_ with 1.2 equiv of DBPO and 20 mol % Bu_4_NI as the best-suited and most economic reagent/catalyst combination, which allowed for the synthesis of **2a** in high isolated yield on 1 mmol scale as well (Table 1, entry 14). Tests using other solvents under these optimized conditions were also carried out (details not given in Table 1, which showed that CH_2_Cl_2_ (95% NMR yield), toluene (95% NMR yield), acetonitrile (90% NMR yield), and THF (85% NMR yield) are also very well-tolerated.

**Table 1:** Optimization of the α-azidation of β-ketoester **1a**^a^.

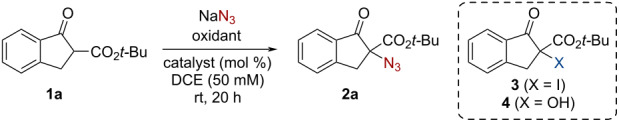						

Entry	Cat. (mol %)	Equiv NaN_3_	Oxidant (equiv)	**2a** (%)^b^	**3** (%)^c^	**4** (%)^c^

1	Bu_4_NI (30)	2.2	H_2_O_2_ (2)	20	0	traces
2	Bu_4_NI (30)	2.2	oxone (2)	85	5	0
3	Bu_4_NI (30)	2.2	mCPBA (2)	20	0	50
4	Bu_4_NI (30)	2.2	*t*-BuOOH (2)	30	traces	60
5	Bu_4_NI (30)	2.2	DBPO (2)	>95	0	0
6	–	2.2	DBPO (2)	0	0	10
7	Bu_4_NBr (30)	2.2	DBPO (2)	>95	0	0
8	Bu_4_NCl (30)	2.2	DBPO (2)	15^d^	0	0
9	Bu_4_NHSO_4_ (30)	2.2	DBPO (2)	0^d^	0	0
10	KI (50)	2.2	DBPO (2)	60	30	0
11	Bu_4_NI (30)	2.2	DBPO (1.2)	>95	0	0
12	Bu_4_NI (30)	2.2	DBPO (0.5)	45	0	0
13	Bu_4_NI (30)	1.2	DBPO (1.2)	>95	0	0
14	Bu_4_NI (20)	1.2	DBPO (1.2)	95 (94)^e^	0	0
15	Bu_4_NI (10)	1.2	DBPO (1.2)	85	0	0

^a^Unless otherwise stated, all reactions were carried out by stirring **1a** (0.1 mmol), the indicated amount of NaN_3_, the catalyst, and the oxidant in 1,2-dichloroethane (DCE, 50 mM based on **1a**) at rt for 20 h. ^b^NMR yield using 1,3,5-trimethoxybenzene as an internal standard (given in 5% intervals). ^c^Determined by ^1^H NMR of the crude product (given in 5% intervals). ^d^Complete conversion of **1a** but giving a rather complex reaction mixture. ^e^Isolated yield on 1 mmol scale.

Having identified high-yielding conditions for the synthesis of **2a**, we next carried out a series of control experiments in order to address the role of the catalyst’s counter anion and the oxidant (Table 2). Running the reaction of **1a** and NaN_3_ in the presence of stoichiometric amounts of Bu_4_NI_3_, I_2_, Bu_4_NIO_3_, or Bu_4_NIO_4_ did not lead to any noteworthy levels of product formation (Table 2, entries 1–4). In sharp contrast, the use of Bu_4_NOH + I_2_, which is known to give Bu_4_NIO in situ [41–44], results in the formation of **2a** in a yield comparable to the above-described catalytic system. Accordingly, and in strong analogy to previous reports [31,41–43], the herein reported protocol most likely proceeds via in situ formation of a catalytically competent quaternary ammonium hypoiodite species which then facilities the coupling of the two inherently nucleophilic reaction partners. To get further mechanistic insights we also carried out our standard reaction (Table 1, entry 14) in the presence of well-established radical traps like TEMPO, di-*tert*-butylhydroxytoluene (BHT), or 1,1-diphenylethene (DPE). In neither case any influence on the yield was observed, thus ruling out a mechanism involving radical species.

**Table 2:** Control experiments using different hypervalent iodine species^a^.

Entry	Oxidant (1 equiv)	**2a** (%)^b^

1	Bu_4_NI_3_	0
2	I_2_	15
3	Bu_4_NIO_3_	0
4	Bu_4_NIO_4_	0
5	I_2_/Bu_4_NOH^c^	95

^a^Carried out by reacting **1a** (0.1 mmol) and NaN_3_ (2.2 equiv) in the presence of 1 equiv of the indicated oxidant in DCE at rt for 20 h. ^b^NMR yield using 1,3,5-trimethoxybenzene as an internal standard (given in 5% intervals). ^c^Resulting in the formation of Bu_4_NIO [41–44].

**Scheme 2:**
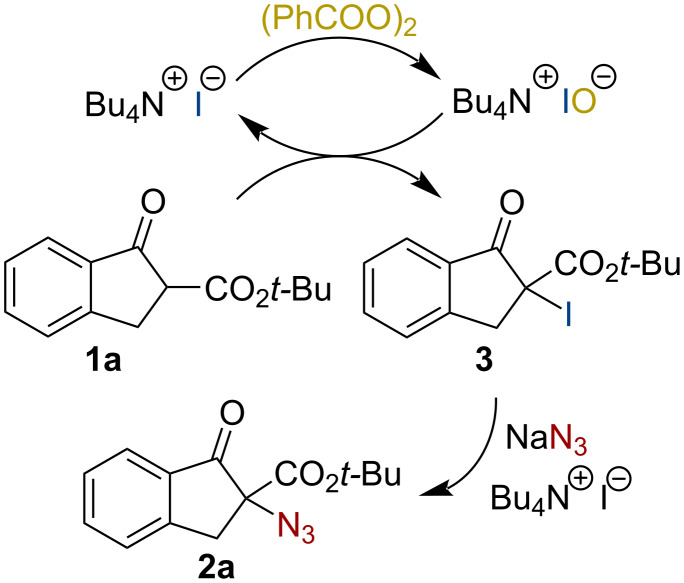
Proposed mechanistic scenario.

Based on these mechanistic details obtained so far, we were also wondering if we could get any hints concerning possible reaction intermediates. Ishihara’s group recently showed that their α-azidation protocol proceeds via in situ α-iodination first, followed by nucleophilic displacement by the azide [31]. Not surprisingly, when we analyzed reactions shortly after the addition of all reagents we detected notable amounts of the α-iodinated β-ketoester **3** which then converted to the final product **2a** over time. Furthermore, we also synthesized compound **3** independently (by reacting **1a** with TBAI and additional KI in the presence of DBPO) and then resubmitted this compound to our ammonium salt-catalyzed azidation reaction conditions, observing full conversion to **2a** as well. Considering all these details we thus propose a mechanistic scenario as outlined in Scheme 2. The catalyst gets oxidized to Bu_4_NIO first, which then facilitates the α-iodination of **1a** (hereby either the formed benzoate or the hypoiodite itself may serve as a base). Intermediate **3** then undergoes a phase-transfer-catalyzed nucleophilic substitution with NaN_3_ thus delivering the final product **2a**.

**Scheme 3:**
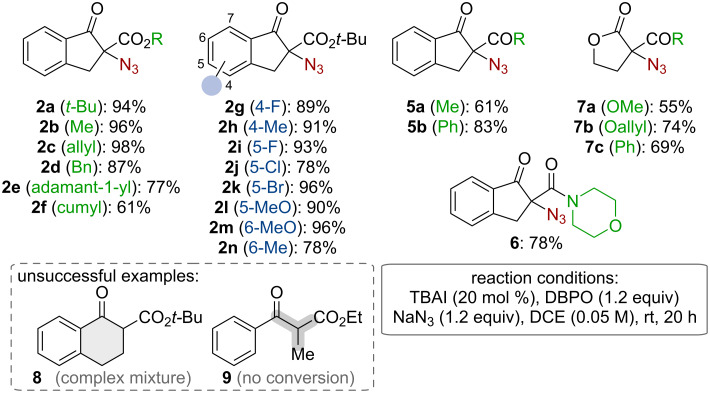
Application scope.

With optimized conditions and a plausible mechanistic understanding at hand, we next investigated the application scope and limitations of this methodology. As outlined in Scheme 3, a series of differently substituted α-azido-β-ketoesters **2** as well as analogous α-azido-β-ketoketones **5** and the α-azido-β-ketoamide **6** could be accessed straightforwardly. Furthermore, this procedure was also successfully extended to γ-butyrolactone-based products **7**. Unfortunately, this methodology came to its limits when using tetralone-based β-ketoesters like compound **8**, which resulted in a complex product mixture, or the acylic β-ketoester **9**, which did not show any conversion under these conditions. The later limitation shows a clear difference between our methodology and Uyanik’s and Ishihara’s protocol [31], underscoring the higher reactivity of their designer catalyst (Scheme 1B).

**Scheme 4:**
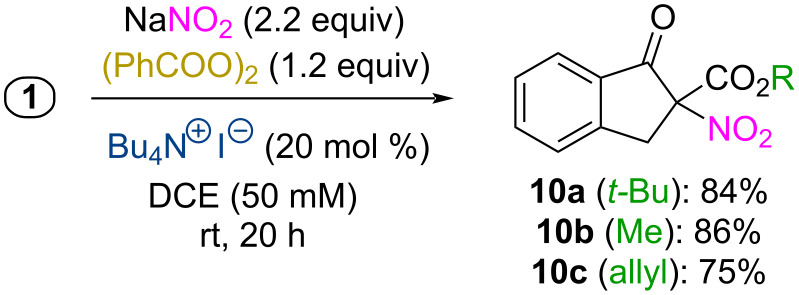
Proof-of-concept for the analogous oxidative α-nitration.

Having established the TBAI/DBPO-mediated α-azidation using NaN_3_, we also briefly tested whether this concept can be extended to an analogous α-nitration approach. Different strategies for α-nitrations of carbonyl compounds have been reported [45–50], but the use of NaNO_2_ under simple oxidative conditions has so far received relatively little attention [51]. Gratifyingly, employing NaNO_2_ (2.2 equiv) under our established oxidative α-azidation conditions we found it possible to access the α-NO_2_-β-ketoesters **10a–c** as well (Scheme 4), which in our opinion represents an interesting proof-of-concept for an ammonium hypoiodite-mediated α-nitration. In this case we observed intermediate formation of the α-iodinated β-ketoester **3** as well (vide supra), which suggests an analogous mechanistic scenario as for the azidation (compare with Scheme 3). However, it should also be stated that the α-nitro products **10** were found to be not too stable, undergoing some unspecific decomposition and also some decarboxylation during column chromatography or upon prolonged reaction times. Also, tests with analogous β-ketoketones and β-ketoamides (compare with azidation products **5** and **6**, Scheme 3) did not give any products but resulted in the formation of a variety of unidentified side-products, thus illustrating that this α-nitration methodology seems to be less general than the α-azidation, which can most likely be attributed to the sensitivity of the products (containing a carbon with three electron-withdrawing groups).

## Conclusion

α-Azidation reactions of carbonyl derivatives are powerful approaches to access valuable organic azides. In this contribution we report the direct α-azidation of cyclic β-ketocarbonyl compounds using NaN_3_. This coupling of two inherently nucleophilic species is possible by carrying out the reaction under oxidative conditions using dibenzoyl peroxide in the presence of a catalytic amount of tetrabutylammonium iodide (TBAI). Control experiments support a mechanistic scenario proceeding via in situ formation of a catalytically competent quaternary ammonium hypoiodite first. This higher oxidation state species then facilitates the α-iodination of the pronucleophile, followed by a phase-transfer-catalyzed nucleophilic substitution by the azide. Furthermore, we also obtained a first proof-of-concept for the conceptually analogous α-nitration by using NaNO_2_ under otherwise identical conditions.

## Experimental

### General details

^1^H, ^13^C and ^19^F NMR spectra were recorded on a Bruker Avance III 300 MHz spectrometer with a broad band observe probe and a sample changer for 16 samples, on a Bruker Avance DRX 500 MHz spectrometer, and on a Bruker Avance III 700 MHz spectrometer with an Ascend magnet and TCI cryoprobe, which are all property of the Austro Czech NMR Research Center “RERI uasb”. All NMR spectra were referenced on the solvent residual peak (CDCl_3_: δ = 7.26 ppm for ^1^H NMR, δ = 77.16 ppm for ^13^C NMR,^19^F NMR unreferenced). IR spectra were recorded on a Bruker Alpha II FTIR spectrometer with diamond ATR-module using the OPUS software package. HRMS spectra were recorded on an Agilent QTOF 6520 spectrometer with an ESI source. Melting points are recorded using a Büchi M-560 apparatus and are reported uncorrected. TLC was performed on Macherey-Nagel pre-coated TLC plates (silica gel, 60 F254, 0.20 mm, ALUGRAM^®^ Xtra SIL). Preparative column chromatography was carried out using Davisil LC 60 Å 70-200 MICRON silica gel. All chemicals were purchased from commercial suppliers and used without further purification unless otherwise stated.

#### General α-azidation procedure

Sodium azide (7.8 mg, 120 µmol, 1.2 equiv) and TBAI (7.4 mg, 20 µmol, 20 mol %) were suspended in a stirred solution of the respective starting material (100 µmol, 1.00 equiv) in 1.0 mL of DCE at rt. Then, a solution of anhydrous dibenzoyl peroxide (29.1 mg, 120 µmol, 1.2 equiv) in 1.0 mL of DCE was added to the suspension and the mixture was stirred for 20 h. The reaction solution was then diluted with 8 mL dichloromethane and extracted with 5 mL of sat. aq NaHCO_3_. The aqueous phase was then extracted twice with 10 mL of DCM. The organic layer and the extracts were then filtered consecutively through a pad of anhydr. sodium sulfate and deactivated silica gel. The solvents were removed in vacuo. In most cases the products were already obtained in sufficiently high purity (>95%) after this work up. If necessary, further purification by silica gel column chromatography can be carried out.

Safety considerations: It is known that the combination of inorganic azides and halogenated compounds can lead to the formation of explosive diazido compounds and thus we have also demonstrated that the azidation chemistry is possible in other solvents as well (vide supra). However, to the best of our knowledge this is mainly an issue with dichloromethane [52–53], whereas the reaction between NaN_3_ and dichloroethane usually requires higher temperatures and represents a slow process [54] and we did not detect any diazidoethane by ^1^H NMR in any of our crude products [55].

**Analytical details for the parent compound 2a**: Obtained in 94% yield (25.7 mg, 94.0 µmol). cf.: 1.0 mmol scale, 94% yield (256.9 mg, 940.0 µmol; purified by column chromatography on silica gel (eluent: heptanes/EtOAc = 19:1)). Yellowish-white solid; Analytical data match those reported in literature [30]. ^1^H NMR (300 MHz, CDCl_3_, 298 K, δ/ppm) 7.82 (d, *J* = 7.7 Hz, 1H), 7.66 (t, *J* = 7.5 Hz, 1H), 7.48–7.39 (m, 2H), 3.64 (d, *J* = 17.2 Hz, 1H), 2.99 (d, *J* = 17.2 Hz, 1H), 1.45 (s, 9H); ^13^C NMR (75 MHz, CDCl_3_, 298 K, δ/ppm) 198.1, 167.4, 152.3, 136.4, 133.3, 128.4, 126.5, 125.6, 84.6, 70.6, 38.6, 28.0; IR (neat, FT-ATR, 298 K, ν̃/cm^−1^): 2984, 2928, 2853, 2110, 1747, 1736, 1718, 1604, 1589, 1548, 1466, 1431, 1397, 1372, 1353, 1326, 1271, 1259, 1215, 1145, 1091, 1054, 1027, 961, 913, 871, 844, 834, 818, 804, 756, 729, 711, 688, 661, 623, 598, 561, 533, 459, 416; HRMS (ESI^+^-QqTOF, *m*/*z*): [M + NH_4_]^+^ calcd for C_14_H_19_N_4_O_3_, 291.1452; found, 291.1452 (major); TLC (silica gel K60, 200 µm, F254, heptanes/EtOAc = 7:3, 298 K, *R*_f_ 0.64; mp: 65.0–67.5 °C.

#### General α-nitration procedure

Sodium nitrite (15.2 mg, 220 µmol, 2.2 equiv) and TBAI (7.4 mg, 20 µmol, 20 mol %) were suspended in a stirred solution of the respective starting material (100 µmol, 1.00 equiv) in 1.0 mL of DCE at rt. Then, a solution of anhydrous dibenzoyl peroxide (29.1 mg, 120 µmol, 1.2 equiv) in 1.0 mL of DCE was added to the suspension and the mixture was stirred for 20 h. The reaction solution was then diluted with 8 mL dichloromethane and extracted with 5 mL of sat. aq NaHCO_3_. The aqueous phase was then extracted twice with 10 mL of DCM. The organic layer and the extracts were then filtered consecutively through a pad of anhydr. sodium sulfate and deactivated silica gel. The solvents were removed in vacuo. In most cases the products were already obtained in sufficient purity (>95%) after this work up. If necessary, further purification can be achieved by fast silica gel column chromatography (the products tend to decompose on silica gel).

**Analytical details for the parent compound 10a**: Obtained in 84% yield (23.3 mg, 94.0 µmol). white solid; ^1^H NMR (700 MHz, CDCl_3_, 298 K, δ/ppm) 7.86 (d, *J* = 7.7 Hz, 1H), 7.71 (t, *J* = 7.5 Hz, 1H), 7.53 (d, *J* = 7.7 Hz, 1H), 7.48 (t, *J* = 7.5 Hz, 1H), 4.11 (d, *J* = 17.9 Hz, 1H), 3.99 (d, *J* = 17.9 Hz, 1H), 1.49 (s, 9H); ^13^C NMR (126 MHz, CDCl_3_, 298 K, δ/ppm) 188.4, 162.0, 150.1, 137.0, 132.9, 129.1, 126.5, 126.2, 96.7, 86.1, 37.5, 27.8; IR (neat, FT-ATR, 298 K, ν̃/cm^−1^): 2984, 2930, 2878, 2854, 1748, 1719, 1656, 1604, 1589, 1548, 1465, 1431, 1396, 1371, 1353, 1325, 1272, 1260, 1215, 1145, 1091, 1056, 1026, 961, 912, 871, 844, 834, 818, 803, 755, 730, 711, 688, 661, 625, 598, 561, 533, 459, 414; HRMS (ESI^+^-QqTOF, *m*/*z*): [M + H]^+^ calcd for C_14_H_16_NO_5_, 278.1023; found, 278.1024; TLC (silica gel K60, 200 µm, F254, heptanes/EtOAc = 7:3, 298 K, *R*_f_ 0.47; mp: 75.9–78.4 °C.

## Supporting Information

File 1 Full experimental and analytical details and copies of NMR spectra.

## Data Availability

All data that supports the findings of this study is available in the published article and/or the supporting information to this article.
