# Supplementary material for: Tetrabutylammonium iodide-catalyzed oxidative α-azidation of β-ketocarbonyl compounds using sodium azide
Source: Beilstein J Org Chem. 2024 Jul 5;20:1510–7. doi: 10.3762/bjoc.20.135 (PMC11228824; doi:10.3762/bjoc.20.135)
Supplement: File 1 — Full experimental and analytical details and copies of NMR spectra. [file Beilstein_J_Org_Chem-20-1510-s001.pdf]

BEILSTEIN JOURNAL OF ORGANIC CHEMISTRY

Supporting Information

for

Tetrabutylammonium iodide-catalyzed oxidative α-azidation of β-ketocarbonyl compounds using sodium azide

Christopher Mairhofer, David Naderer and Mario Waser

Beilstein J. Org. Chem. **2024,** *20,* 1510–1517. doi:10.3762/bjoc.20.135

Full experimental and analytical details and copies of NMR spectra

License and Terms: This is a supporting information file under the terms of the Creative Commons Attribution License (https://creativecommons.org/licenses/by/4.0). Please note that the reuse, redistribution and reproduction in particular requires that the author(s) and source are credited and that individual graphics may be subject to special legal provisions.
The license is subject to the *Beilstein Journal of Organic Chemistry* terms and conditions: (https://www.beilstein-journals.org/bjoc/terms)

Table of Contents

1. General information S2

2. Handling of azido compounds S3

3. Synthesis of substrates S4

4. Oxidative α-azidation reactions S5

General procedure S5

Characterization of the α-azidation products S5

5. Oxidative α-nitration reactions S16

General procedure S16

Characterization of the α-nitration products S16

6. NMR spectra S18

7. Infrared spectra S43

8. High-resolution mass spectra S55

9. References S67

1. General Information

Nuclear magnetic resonance (NMR) spectra were recorded on a Bruker Avance III 300 MHz spectrometer with a broad band observe probe and a sample changer for 16 samples, on a Bruker Avance DRX 500 MHz spectrometer, and on a Bruker Avance III 700 MHz spectrometer with an Ascend magnet and TCI cryoprobe, which are all property of the Austro Czech NMR Research Center "RERI uasb". All NMR spectra were referenced on the solvent residual peak ($CDCl_3$: δ = 7.26 ppm for ^{1}H NMR, δ = 77.16 ppm for ^{13}C NMR, ^{19}F NMR is unreferenced). ^{1}H NMR and ^{19}F NMR spectra are reported as follows: chemical shift (δ/ppm) (multiplicity, coupling constants, number of protons). Peak multiplicities are denoted as: s = singlet, d = doublet, t = triplet, q = quartet, m = multiplet, br = broad, dd = doublet of doublet, etc. Infrared (IR) spectra were recorded on a Bruker Alpha II FTIR spectrometer with diamond ATR-module using OPUS software package and are reported in $\bar{\nu}$/cm^{-1}. High-resolution mass spectra (HRMS) were recorded on an Agilent QTOF 6520 with an ESI source, owned and operated by the Institute of Analytical and General Chemistry, JKU. Melting points (MP) are recorded using a Büchi M-560 apparatus and are reported uncorrected in ϑ_m/°C. Thin layer chromatography (TLC) was performed on Macherey-Nagel pre-coated TLC plates (silica gel, 60 F_{254}, 0.20 mm, ALUGRAM® Xtra SIL). TLC plates were visualized by irradiation with UV light at λ_{max} = 254 nm or using permanent staining with permanganate. Preparative column chromatography was carried out using Davisil LC 60A 70–200 MICRON silica gel. The term in vacuo refers to removal of solvents by rotary evaporation (40 °C, 950–20 mbar) followed by drying at high vacuum. All chemicals were purchased from commercial suppliers and used without further purification unless otherwise stated.

Anhydrous dibenzoyl peroxide (DBPO) was prepared from the commercially available 70–75% hydrate:[1] To a solution of wet dibenzoyl peroxide (10 g, 75%, remainder water, Sigma-Aldrich) in 100 mL of CH_2Cl_2 was added anhydrous Na_2SO_4 (10 g) at 0 °C. The suspension was stirred for 30 min and then filtered through a sintered glass funnel. The solvent was removed in vacuo (water bath temperature <40 °C) and dried under high vacuum to obtain a free-flowing white solid which was stored at 4 °C under argon.

Deactivated silica gel was prepared from the commercially available Davisil LC 60A 70–200 MICRON silica gel: To a suspension of silica gel (300 g) in 400 mL of Et_2O, triethylamine (10.0 mL) was added. Then, the solvent was removed in vacuo and dried under high vacuum to obtain a free-flowing white solid which was stored on the bench.

2. Handling of azido compounds[2]

Sodium azide (NaN_3, CAS: 26628-22-8) is acutely toxic ($LD_{50,\ mouse}$ = 27 mg kg^{-1}) and should be handled only under adequate safety precautions. Sodium azide reacts with acids to form hydrazoic acid (HN_3) which is both toxic ($LD_{50,\ mouse}$ = 22 mg kg^{-1}) and a spontaneously explosive gas. Excess NaN_3 adhered on any surface (flasks, paper, etc.) should be destroyed in a fume hood by soaking with acidified sodium nitrite or by oxidation with cerium(IV) ammonium nitrate.[2g] Organic azides are potentially explosive chemicals (PECs) that decompose with introduction of external energy (heat, light, shock and pressure). Azides with a (C + O) / N ratio of <1 are potentially explosive and should never be isolated. Azides with a (C + O) / N ratio between 1 and 3 can be isolated but should be stored as solutions (≤1 M) below room temperature with less than 5 grams of material. Azides with a (C + O) / N ratio of ≥3 are normally stable and can be isolated and stored in pure form. Any azide synthesized should be stored below room temperature and in the dark.

3. Synthesis of Substrates

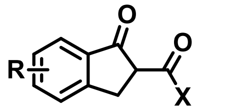

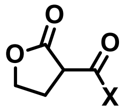

Indanone based ketoesters:
X = OtBu, R = H: **1a**
X = OMe, R = H: **1b**
X = Oallyl, R = H: **1c**
X = OBn, R = H: **1d**
X = OAd, R = H: **1e**
X = OCum, R = H: **1f**

Indanone based ketoesters:
X = OtBu, R = 4-F: **1g**
X = OtBu, R = 4-Me: **1h**
X = OtBu, R = 5-F: **1i**
X = OtBu, R = 5-Cl: **1j**
X = OtBu, R = 5-Br: **1k**
X = OtBu, R = 5-OMe: **1l**
X = OtBu, R = 6-OMe: **1m**
X = OtBu, R = 6-Me: **1n**

Indanone based diketones:
X = Me, R = H: **1p**
X = Ph, R = H: **1q**

Indanone based ketoamides:
X = Nmorph, R = H: **1r**

Lactone based ketoesters:
X = OMe: **11a**
X = Oallyl: **11b**
X = Oallyl: **11c**

All substrates are known compounds and prepared by following the literature procedures:

The indanone based *tert*-butyl β-ketoesters **1a**[3,4], **1g–n**[3,4] were prepared starting from 1-indanone, CAS 83-33-0 or the respective commercially available 1-indanone derivatives using Boc-pyrrole, CAS 5176-27-2 as transfer-reagent. The other indanone based β-ketoesters **1b**[5], **1c**[6], were prepared starting from 1-indanone and their respective commercially available dialkyl carbonates. The indanone based β-ketoesters **1d–f**[7] as well as the β-ketoamide **1r**[8] were prepared starting from methyl 1-indanone-2-carboxylate (**1b**). The indanone based 1,3-diketones **1p-q**[3,4] were prepared according to a very similar procedure, but using imidazole based transfer reagents. These 1-acylimidazoles[4,9] which are prepared from imidazole, CAS 288-32-4 and the respective commercially available acyl chlorides for diketones or chloroformates for β-ketoesters. Since these 1-acylimidazole reagents are more reactive then Boc-pyrrole, the reaction can be carried out at room temperature and is also a powerful alternative to prepare the β-ketoesters **1a–c**. The lactone based β-ketoesters **11a–c**[10,11,12] were prepared from γ-butyrolactone, CAS 96-48-0 and the respective commercially available dialkyl carbonates or acyl chlorides, whereas also in this case the use of the previously described 1-acylimidazole transfer reagents turned out to be beneficial in terms of yield and selectivity.

4. Oxidative α-azidation reactions

General procedure

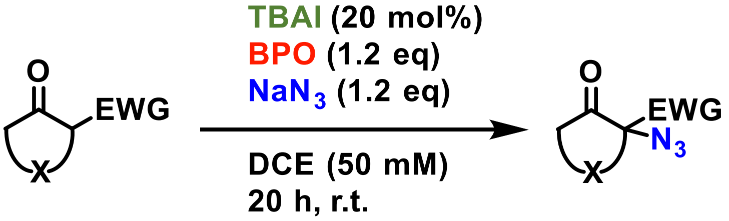

Sodium azide (7.8 mg, 120 µmol, 1.2 equiv) and TBAI (7.4 mg, 20 µmol, 20 mol %) were suspended in a stirred solution (900–1000 rpm) of the respective substrate (100 µmol, 1.00 equiv) in 1.0 mL of DCE at rt. Then, a solution of anhydrous benzoyl peroxide (29.1 mg, 120 µmol, 1.2 equiv) in 1.0 mL of DCE was added to the suspension and stirred for 20 h.

The reaction solution was then diluted with 8 mL dichloromethane and extracted with 5 mL of sat. aq $NaHCO_3$. The aqueous phase was then extracted twice with 10 mL of DCM. The organic layer and the extracts were then filtered consecutively through a pad of anhydr. sodium sulfate and deactivated silica gel. The solvents were removed in vacuo. In most cases the products were already obtained in high purity (>95 %) after this work up. If necessary, further purification can be achieved by silica gel column chromatography.

Characterization of the α-azidation products

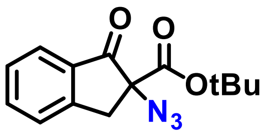

***tert*-Butyl 2-azido-1-oxo-2,3-dihydro-1*H*-indene-2-carboxylate (2a):**[13,14,15,16,17] Obtained in 94% yield (25.7 mg, 94.0 µmol). *cf.*: 1.0 mmol scale, 94% yield (256.9 mg, 940.0 µmol). This compound was purified by column chromatography on silica gel (eluent: heptanes/EtOAc = 19:1). Yellowish-white solid; **^{1}H-NMR** (300 MHz, $CDCl_3$, 298 K, δ / ppm): 7.82 (d, J = 7.7 Hz, 1H), 7.66 (t, J = 7.5 Hz, 1H), 7.48 - 7.39 (m, 2H), 3.64 (d, J = 17.2 Hz, 1H), 2.99 (d, J = 17.2 Hz, 1H), 1.45 (s, 9H); **^{13}C-NMR** (75 MHz, $CDCl_3$, 298 K, δ / ppm): 198.1, 167.4, 152.3, 136.4, 133.3, 128.4, 126.5, 125.6, 84.6, 70.6, 38.6, 28.0; **IR** (neat, FT-ATR, 298 K, $\tilde{\nu}$ / cm^{-1}): 2984, 2928, 2853, 2110, 1747, 1736, 1718, 1604, 1589, 1548, 1466, 1431, 1397, 1372, 1353, 1326, 1271, 1259, 1215, 1145, 1091, 1054, 1027, 961, 913, 871, 844, 834, 818, 804, 756, 729, 711, 688, 661, 623, 598, 561, 533, 459, 416; **HRMS** (ESI$^+$-QqTOF, *m*/*z*): calculated for $C_{14}H_{16}N_3O_3$ $[M+H]^+$: 274.1186, found: 274.1186; calculated for $C_{14}H_{19}N_4O_3$ $[M+NH_4]^+$: 291.1452, found: 291.1452 (major); calculated for $C_{14}H_{15}N_3O_3Na$ $[M+Na]^+$: 296.1006, found: 296.1006; calculated for $C_{14}H_{15}N_3O_3K$ $[M+K]^+$: 312.0745, found: 312.0745, **TLC** (silica gel K60, 200 µm, F254, heptanes/EtOAc = 7:3, 298 K, R_f / 1): 0.64; **MP** (uncorrected, ϑ_m / °C): 65.0 - 67.5.

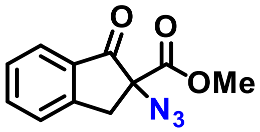

Methyl 2-azido-1-oxo-2,3-dihydro-1*H*-indene-2-carboxylate (2b):[14,16] Obtained in 96% yield (22.2 mg, 96.0 µmol); white solid; **^{1}H-NMR** (500 MHz, $CDCl_3$, 298 K, δ / ppm): 7.83 (d, J = 7.7 Hz, 1H), 7.69 (t, J = 7.5 Hz, 1H), 7.55 - 7.41 (m, 2H), 3.81 (s, 3H), 3.68 (d, J = 17.4 Hz, 1H), 3.04 (d, J = 17.3 Hz, 1H); **^{13}C-NMR** (126 MHz, $CDCl_3$, 298 K, δ / ppm): 197.5, 169.1, 152.2, 136.6, 133.1, 128.6, 126.6, 125.8, 70.3, 53.7, 38.6; **IR** (neat, FT-ATR, 298 K, $\bar{\nu}$ / cm^{-1}): 3475, 3409, 3187, 3082, 3040, 3010, 2956, 2922, 2851, 2449, 2428, 2326, 2278, 2178, 2135, 2099, 2081, 1744 ,1710, 1601, 1587, 1479, 1467, 1440, 1427, 1344, 1326, 1305, 1278, 1218, 1186, 1161, 1122, 1098, 1050, 998, 965, 951, 903, 826, 806, 777, 739, 696, 626, 599, 550, 469, 437, 406; **HRMS** (ESI^{+}-QqTOF, *m*/*z*): calculated for $C_{11}H_{10}N_3O_3$ $[M+H]^{+}$: 232.0717, found: 232.0717; calculated for $C_{11}H_{13}N_4O_3$ $[M+NH_4]^{+}$: 249.0982, found: 249.0982 (major), calculated for $C_{11}H_9N_3O_3Na$ $[M+Na]^{+}$: 254.0536, found: 254.0539; calculated for $C_{11}H_9N_3O_3K$ $[M+K]^{+}$: 270.0275, found: 270.0275; **TLC** (silica gel K60, 200 µm, F254, heptanes/EtOAc = 7:3, 298 K, R_f / 1): 0.50; **MP** (uncorrected, ϑ_m / °C): 54.7 - 56.5.

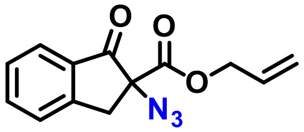

Allyl 2-azido-1-oxo-2,3-dihydro-1*H*-indene-2-carboxylate (2c): Obtained in 98% yield (25.2 mg, 98.0 µmol); yellow solid; **^{1}H-NMR** (500 MHz, $CDCl_3$, 298 K, δ / ppm): 7.84 (d, J = 7.7 Hz, 1H), 7.69 (t, J = 7.5 Hz, 1H), 7.47 (d, J = 7.8 Hz, 1H), 7.46 (t, J = 7.5 Hz, 1H), 5.85 (ddt, J = 17.2, 10.5, 5.6 Hz, 1H), 5.29 - 5.19 (m, 2H), 4.70 (tt, J = 5.8, 1.4 Hz, 2H), 3.69 (d, J = 17.3 Hz, 1H), 3.05 (d, J = 17.3 Hz, 1H); **^{13}C-NMR** (126 MHz, $CDCl_3$, 298 K, δ / ppm): 197.4, 168.3, 152.2, 136.6, 133.2, 130.9, 128.6, 126.6, 125.8, 119.3, 70.4, 67.2, 38.7; **IR** (neat, FT-ATR, 298 K, $\bar{\nu}$ / cm^{-1}): 3077, 2954, 2923, 2852, 2109, 1744, 1713, 1649, 1605, 1588, 1464, 1426, 1377, 1362, 1328, 1265, 1227, 1213, 1179, 1124, 1089, 1046, 994, 938, 908, 865, 826, 806, 749, 697, 617, 599, 550, 468, 423; **HRMS** (ESI^{+}-QqTOF, *m*/*z*): calculated for $C_{13}H_{12}N_3O_3$ $[M+H]^{+}$: 258.0873, found: 258.0874; calculated for $C_{13}H_{15}N_4O_3Na$ $[M+NH_4]^{+}$: 275.1139, found: 275.1139 (major), calculated for $C_{13}H_{11}N_3O_3K$ $[M+K]^{+}$: 296.0432, found: 296.0432; **TLC** (silica gel K60, 200 µm, F254, heptanes/EtOAc = 7:3, 298 K, R_f / 1): 0.55; **MP** (uncorrected, ϑ_m / °C): 80.1 - 84.7.

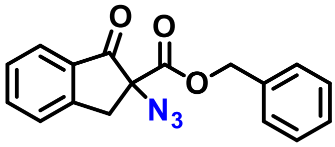

Benzyl 2-azido-1-oxo-2,3-dihydro-1*H*-indene-2-carboxylate (2d): Obtained in 87% yield (26.7 mg, 87.0 µmol); yellow oil; **^{1}H-NMR** (500 MHz, $CDCl_3$, 298 K, δ / ppm): 7.84 (d, J = 7.9 Hz, 1H), 7.68 (t, J = 7.4 Hz, 1H), 7.49 - 7.43 (m, 2H), 7.36 - 7.31 (m, 3H), 7.28 - 7.24 (m, 2H), 5.30 (d, J = 12.3 Hz, 1H), 5.20 (d, J = 12.3 Hz, 1H), 3.66 (d, J = 17.3 Hz, 1H), 3.04 (d, J = 17.3 Hz, 1H); **^{13}C-NMR** (126 MHz, $CDCl_3$, 298 K, δ / ppm): 197.4, 168.5, 152.1, 136.6, 134.8, 133.2, 128.8, 128.7, 128.6, 128.2, 126.6, 125.8, 70.4, 68.4, 38.6; **IR** (neat, FT-ATR, 298 K, $\bar{\nu}$ / cm^{-1}): 3474, 3407, 3343, 3194, 3096, 3066, 3032, 2981, 2930, 2848, 2681, 2638, 2607, 2485, 2442, 2359, 2317, 2110, 1954, 1920, 1742, 1710, 1607, 1583, 1491, 1465, 1437, 1392, 1370, 1339, 1292, 1260, 1243, 1212, 1168, 1151, 1132, 1099, 1050, 1026, 961, 924, 904, 892, 866, 839, 798, 749, 730, 700, 653, 617, 601, 561, 548, 497, 468, 441, 416; **HRMS** (ESI$^+$-QqTOF, *m/z*): calculated for $C_{17}H_{14}N_3O_3$ $[M+H]^+$: 308.1030, found: 308.1032; calculated for $C_{17}H_{17}N_4O_3$ $[M+NH_4]^+$: 325.1295, found: 325.1295 (major); calculated for $C_{17}H_{13}N_3O_3Na$ $[M+Na]^+$: 330.0849, found: 330.0849; calculated for $C_{17}H_{13}N_3O_3K$ $[M+K]^+$: 346.0588, found: 346.0588; **TLC** (silica gel K60, 200 µm, F254, heptanes/EtOAc = 7:3, 298 K, R_f / 1): 0.55; **MP** (uncorrected, ϑ_m / °C): < r.t.

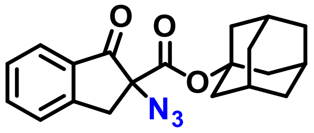

Adamantyl 2-azido-1-oxo-2,3-dihydro-1*H*-indene-2-carboxylate (2e):[13,16] Obtained in 77% yield (27.1 mg, 77.0 µmol); colourless oil; **^{1}H-NMR** (500 MHz, $CDCl_3$, 298 K, δ / ppm): 7.81 (d, J = 7.7 Hz, 1H), 7.66 (t, J = 7.6 Hz, 1H), 7.55 - 7.38 (m, 2H), 3.64 (d, J = 17.1 Hz, 1H), 2.98 (d, J = 17.1 Hz, 1H), 2.15 (s, 3H), 2.07 (d, J = 3.0 Hz, 6H), 1.63 (t, J = 2.8 Hz, 6H); **^{13}C-NMR** (126 MHz, $CDCl_3$, 298 K, δ / ppm): 198.2, 167.1, 152.4, 136.3, 133.4, 128.4, 126.4, 125.6, 84.7, 70.6, 41.2, 38.7, 36.1, 31.0; **IR** (neat, FT-ATR, 298 K, $\bar{\nu}$ / cm^{-1}): 2911, 2853, 2110, 1742, 1714, 1607, 1589, 1457, 1426, 1355, 1264, 1230, 1213, 1182, 1155, 1103, 1090, 1046, 963, 912, 862, 835, 806, 749, 722, 698, 636, 549, 469, 419; **HRMS** (ESI$^+$-QqTOF, *m/z*): calculated for $C_{20}H_{22}N_3O_3$ $[M+H]^+$: 352.1656, found: 352.1653; calculated for $C_{20}H_{26}N_4O_3$ $[M+NH_4]^+$: 369.1921, found: 369.1922 (major); calculated for $C_{20}H_{22}N_3O_3Na$ $[M+Na]^+$: 374.1475, found: 374.1478; calculated for $C_{20}H_{22}N_3O_3K$ $[M+K]^+$: 390.1214, found: 390.1213; **TLC** (silica gel K60, 200 µm, F254, heptanes/EtOAc = 7:3, 298 K, R_f / 1): 0.65; **MP** (uncorrected, ϑ_m / °C): < r.t.

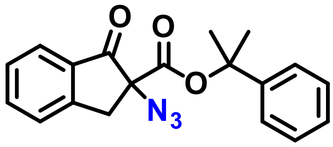

2-Phenylpropan-2-yl 2-azido-1-oxo-2,3-dihydro-1*H*-indene-2-carboxylate (2f):[13] Obtained in 61% yield (20.5 mg, 61.0 µmol); yellow oil; **^{1}H-NMR** (500 MHz, $CDCl_3$, 298 K, δ / ppm): 7.85 (d, J = 7.7 Hz, 1H), 7.68 (t, J = 7.6 Hz, 1H), 7.52 - 7.43 (m, 1H), 7.33 - 7.27 (m, 1H), 7.27 - 7.20 (m, 2H), 3.67 (d, J = 17.2 Hz, 1H), 3.03 (d, J = 17.2 Hz, 1H), 1.81 (s, 3H), 1.77 (s, 3H); **^{13}C-NMR** (126 MHz, $CDCl_3$, 298 K, δ / ppm): 197.9, 166.7, 152.2, 144.5, 136.4, 133.4, 128.5, 128.5, 127.6, 126.5, 125.6, 124.3, 85.5, 70.6, 38.6, 28.7, 28.1; **IR** (neat, FT-ATR, 298 K, $\bar{\nu}$ / cm^{-1}): 2983, 2933, 2111, 1746, 1715, 1606, 1588, 1496, 1465, 1449, 1428, 1385, 1368, 1264, 1231, 1214, 1133, 1101, 1044, 1030, 939, 909, 835, 809, 751, 697, 628, 592, 551, 468, 420; **HRMS** (ESI^+-QqTOF, *m/z*): calculated for $C_{19}H_{21}N_4O_3$ $[M+NH_4]^+$: 353.1608, found: 353.1608 (major); calculated for $C_{19}H_{17}N_3O_3Na$ $[M+Na]^+$: 358.1162, found: 358.1163; calculated for $C_{19}H_{17}N_3O_3K$ $[M+K]^+$: 374.0901, found: 374.0901; **TLC** (silica gel K60, 200 µm, F254, heptanes/EtOAc = 7:3, 298 K, R_f / 1): 0.57; **MP** (uncorrected, ϑ_m / °C): < r.t.

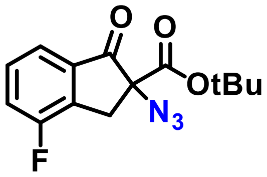

***tert*-Butyl 2-azido-4-fluoro-1-oxo-2,3-dihydro-1*H*-indene-2-carboxylate (2g):** Obtained in 89% yield (25.9 mg, 89.0 µmol); yellow oil; **^{1}H-NMR** (500 MHz, $CDCl_3$, 298 K, δ / ppm): 7.63 (d, J = 7.5 Hz, 1H), 7.45 (td, J = 7.8, 4.5 Hz, 1H), 7.35 (t, J = 8.3 Hz, 1H), 3.64 (d, J = 17.5 Hz, 1H), 2.98 (d, J = 17.5 Hz, 1H), 1.46 (s, 9H); **^{13}C-NMR** (126 MHz, $CDCl_3$, 298 K, δ / ppm): 197.1 (d, J = 2.8 Hz), 167.0, 159.7 (d, J = 251.6 Hz), 138.2 (d, J = 19.5 Hz), 136.0 (d, J = 5.0 Hz), 130.4 (d, J = 6.4 Hz), 122.5 (d, J = 20.0 Hz), 121.3 (d, J = 4.0 Hz), 85.0, 70.3, 34.6, 28.0; **^{19}F-NMR** (471 MHz, $CDCl_3$, 298 K, δ / ppm): -118.29 (dd, J = 8.6, 4.5 Hz, 1F); **IR** (neat, FT-ATR, 298 K, $\bar{\nu}$ / cm^{-1}): 2981, 2933, 2116, 1746, 1722, 1619, 1594, 1482, 1458, 1426, 1395, 1371, 1339, 1264, 1244, 1186, 1147, 1048, 1035, 992,868, 833, 813, 774, 746, 717, 686, 580, 551, 500, 469, 463; **HRMS** (ESI^+-QqTOF, *m/z*): calculated for $C_{14}H_{18}FN_4O_3$ $[M+NH_4]^+$: 309.1357, found: 309.1357 (major); calculated for $C_{14}H_{14}FN_3O_3Na$ $[M+Na]^+$: 314.0911, found: 314.0911; calculated for $C_{14}H_{14}FN_3O_3K$ $[M+K]^+$: 330.0651, found: 330.0651; **TLC** (silica gel K60, 200 µm, F254, heptanes/EtOAc = 7:3, 298 K, R_f / 1): 0.67; **MP** (uncorrected, ϑ_m / °C): < r.t.

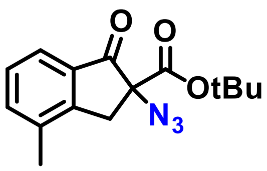

***tert*-Butyl 2-azido-4-methyl-1-oxo-2,3-dihydro-1*H*-indene-2-carboxylate (2h):** Obtained in 91% yield (26.1 mg, 91.0 µmol); yellowish oil; **^{1}H-NMR** (500 MHz, $CDCl_3$, 298 K, *δ* / ppm): 7.65 (d, *J* = 7.6 Hz, 1H), 7.47 (d, *J* = 7.3 Hz, 1H), 7.35 (t, *J* = 7.5 Hz, 1H), 3.52 (d, *J* = 17.2 Hz, 1H), 2.86 (d, *J* = 17.2 Hz, 1H), 2.33 (s, 3H), 1.47 (s, 9H); **^{13}C-NMR** (126 MHz, $CDCl_3$, 298 K, *δ* / ppm): 198.3, 167.6, 151.3, 136.9, 135.8, 133.1, 128.6, 123.0, 84.6, 70.5, 37.6, 28.0, 17.9; **IR** (neat, FT-ATR, 298 K, $\bar{\nu}$ / cm^{-1}): 3079, 2980, 2932, 2870, 2444, 2361, 2297, 2112, 1742, 1715, 1606, 1592, 1479, 1458, 1421, 1395, 1370, 1336, 1264, 1250, 1201, 1148, 1050, 1027, 953, 895, 859, 839, 830, 813, 769, 746, 721, 691, 661, 626, 574, 550, 509, 461; **HRMS** (ESI^+-QqTOF, *m*/*z*): calculated for $C_{15}H_{18}N_3O_3$ $[M+H]^+$: 288.1343, found: 288.1343; calculated for $C_{15}H_{21}N_4O_3$ $[M+NH_4]^+$: 305.1608, found: 305.1608 (major); calculated for $C_{15}H_{17}N_3O_3Na$ $[M+Na]^+$: 310.1162, found: 310.1162; calculated for $C_{15}H_{17}N_3O_3K$ $[M+K]^+$: 326.0901, found: 326.0901; **TLC** (silica gel K60, 200 µm, F254, heptanes/EtOAc = 7:3, 298 K, R_f / 1): 0.67; **MP** (uncorrected, ϑ_m / °C): 65.0 - 67.5.

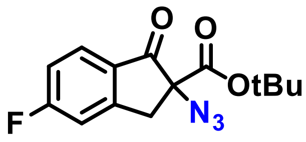

***tert*-Butyl 2-azido-5-fluoro-1-oxo-2,3-dihydro-1*H*-indene-2-carboxylate (2i):**[13] Obtained in 93% yield (27.1 mg, 93.0 µmol); yellowish solid; **^{1}H-NMR** (500 MHz, $CDCl_3$, 298 K, *δ* / ppm): 7.82 (dd, *J* = 8.3, 5.2 Hz, 1H), 7.19 - 7.04 (m, 2H), 3.62 (d, *J* = 17.4 Hz, 1H), 2.96 (d, *J* = 17.4 Hz, 1H), 1.45 (s, 9H); **^{13}C-NMR** (126 MHz, $CDCl_3$, 298 K, *δ* / ppm): 196.2, 168.1 (d, *J* = 259.3 Hz), 167.1, 155.4 (d, *J* = 10.5 Hz), 129.7 (d, *J* = 1.8 Hz), 128.0 (d, *J* = 10.7 Hz), 116.9 (d, *J* = 23.8 Hz), 113.3 (d, *J* = 22.8 Hz), 84.8, 70.7, 38.5 (d, *J* = 2.2 Hz), 28.0; **^{19}F-NMR** (471 MHz, $CDCl_3$, 298 K, *δ* / ppm): -99.4 (dt, *J* = 8.5, 5.1 Hz, 1F).; **IR** (neat, FT-ATR, 298 K, $\bar{\nu}$ / cm^{-1}): 3432, 3102, 3073, 2988, 2925, 2853, 2675, 2426, 2289, 2242, 2107, 1920, 1796, 1734, 1720, 1614, 1593, 1483, 1455, 1432, 1398, 1371, 1335, 1298, 1253, 1192, 1148, 1086, 1046, 942, 906, 900, 867, 830, 802, 787, 759, 738, 691, 655, 629, 620, 551, 503, 476, 438; **HRMS** (ESI^+-QqTOF, *m*/*z*): calculated for $C_{14}H_{15}FN_3O_3$ $[M+H]^+$: 309.1357, found: 309.1357; calculated for $C_{14}H_{18}FN_4O_3$ $[M+NH_4]^+$: 309.1357, found: 309.1357 (major); calculated for $C_{14}H_{14}FN_3O_3Na$ $[M+Na]^+$: 314.0911, found: 314.0911; calculated for $C_{14}H_{14}FN_3O_3K$ $[M+K]^+$: 330.0651, found: 330.0651; **TLC** (silica gel K60, 200 µm, F254, heptanes/EtOAc = 7:3, 298 K, R_f / 1): 0.67; **MP** (uncorrected, ϑ_m / °C): 62.0 - 65.0

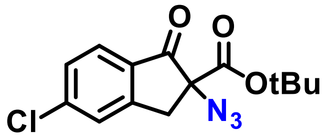

***tert*-Butyl 2-azido-5-chloro-1-oxo-2,3-dihydro-1*H*-indene-2-carboxylate (2j):** Obtained in 78% yield (24.0 mg, 78.0 µmol); yellow solid; **^{1}H-NMR** (500 MHz, $CDCl_3$, 298 K, δ / ppm): 7.74 (d, J = 8.2 Hz, 1H), 7.46 (s, 1H), 7.42 (d, J = 8.2 Hz, 1H), 3.61 (d, J = 17.4 Hz, 1H), 2.96 (d, J = 17.4 Hz, 1H), 1.46 (s, 9H); **^{13}C-NMR** (126 MHz, $CDCl_3$, 298 K, δ / ppm): 196.8, 167.1, 153.7, 143.1, 131.8, 129.3, 126.7, 126.6, 84.9, 70.6, 38.3, 28.0; **IR** (neat, FT-ATR, 298 K, $\bar{\nu}$ / cm^{-1}): 3093, 3065, 2989, 2928, 2854, 2359, 2326, 2293, 2164, 2116, 1918, 1742, 1716, 1599, 1578, 1456, 1423, 1396, 1370, 1323, 1297, 1279, 1261, 1245, 1210, 1146, 1069, 1048, 914, 868, 834, 797, 787, 755, 735, 722, 688, 612, 550, 524, 480, 451, 435; **HRMS** (ESI$^+$-QqTOF, *m*/*z*): calculated for $C_{14}H_{15}{}^{35}ClN_3O_3$ $[M+H]^+$: 308.0796, found: 308.0799; calculated for $C_{14}H_{18}{}^{35}ClN_4O_3$ $[M+NH_4]^+$: 325.1062, found: 325.1061 (major); calculated for $C_{14}H_{18}{}^{37}ClN_4O_3$ $[M+NH_4]^+$: 327.1037, found: 327.1038; calculated for $C_{14}H_{14}{}^{35}ClN_3O_3Na$ $[M+Na]^+$: 330.0616, found: 330.0616; calculated for $C_{14}H_{14}{}^{35}ClN_3O_3K$ $[M+K]^+$: 346.0355, found: 346.0355; calculated for $C_{14}H_{14}{}^{37}ClN_3O_3K$ $[M+K]^+$: 348.0331, found: 348.0331; **TLC** (silica gel K60, 200 µm, F254, heptanes/EtOAc = 7:3, 298 K, R_f / 1): 0.67; **MP** (uncorrected, ϑ_m / °C): 76.0 - 78.9.

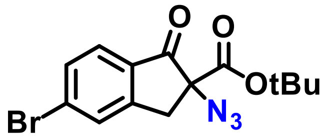

***tert*-Butyl 2-azido-5-bromo-1-oxo-2,3-dihydro-1*H*-indene-2-carboxylate (2k):**[13,15] Obtained in 96% yield (33.8 mg, 96.0 µmol); white solid; **^{1}H-NMR** (500 MHz, $CDCl_3$, 298 K, δ / ppm): 7.67 (d, J = 8.2 Hz, 1H), 7.64 (s, 1H), 7.58 (d, J = 8.2 Hz, 1H), 3.61 (d, J = 17.4 Hz, 1H), 2.96 (d, J = 17.4 Hz, 1H), 1.46 (s, 9H); **^{13}C-NMR** (126 MHz, $CDCl_3$, 298 K, δ / ppm): 197.0, 167.0, 153.8, 132.2, 132.1, 132.0, 129.8, 126.7, 84.9, 70.5, 38.2, 28.0; **IR** (neat, FT-ATR, 298 K, $\bar{\nu}$ / cm^{-1}): 3089, 3002, 2981, 2926, 2852, 2493, 2308, 2162, 2111, 1816, 1736, 1718, 1592, 1468, 1455, 1418, 1396, 1371, 1322, 1299, 1280, 1244, 1212, 1196, 1177, 1143, 1212, 1196, 1177, 1143, 1131, 1101, 1058, 1046, 1036, 913, 868, 847, 834, 792, 749, 730, 713, 687, 615, 596, 550, 517, 477, 460, 439, 424, 406; **HRMS** (ESI$^+$-QqTOF, *m*/*z*): calculated for $C_{14}H_{15}{}^{79}BrN_3O_3$ $[M+H]^+$: 352.0291, found: 352.0291; calculated for $C_{14}H_{15}{}^{81}BrN_3O_3$ $[M+H]^+$: 354.0272, found: 354.0272; calculated for $C_{14}H_{18}{}^{79}BrN_4O_3$ $[M+NH_4]^+$: 369.0557, found: 369.0557; calculated for $C_{14}H_{18}{}^{81}BrN_4O_3$ $[M+NH_4]^+$: 371.0538, found: 369.0538 (major); calculated for $C_{14}H_{14}{}^{79}BrN_3O_3K$ $[M+K]^+$: 389.9850, found: 389.9850; calculated for $C_{14}H_{14}{}^{81}BrN_3O_3K$ $[M+K]^+$: 391.9831, found: 391.9831; **TLC** (silica gel K60, 200 µm, F254, heptanes/EtOAc = 7:3, 298 K, R_f / 1): 0.68; **MP** (uncorrected, ϑ_m / °C): 91.3 - 93.6.

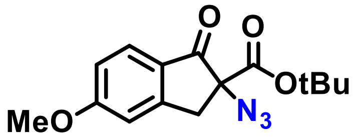

***tert*-Butyl 2-azido-5-methoxy-1-oxo-2,3-dihydro-1*H*-indene-2-carboxylate (2l):**[15] Obtained in 90% yield (27.3 mg, 90.0 µmol); yellow oil; **^{1}H-NMR** (500 MHz, $CDCl_3$, 298 K, δ / ppm): 7.73 (d, *J* = 8.5 Hz, 1H), 6.94 (d, *J* = 8.9 Hz, 1H), 6.86 (s, 1H), 3.89 (s, 3H), 3.57 (d, *J* = 17.2 Hz, 1H), 2.91 (d, *J* = 17.1 Hz, 1H), 1.45 (s, 9H); **^{13}C-NMR** (126 MHz, $CDCl_3$, 298 K, δ / ppm): 195.9, 167.7, 166.6, 155.5, 127.3, 126.3, 116.5, 109.6, 84.4, 70.9, 55.9, 38.6, 28.0; **IR** (neat, FT-ATR, 298 K, $\tilde{\nu}$ / cm^{-1}): 3476, 3406, 3191, 3096, 3066, 3031, 2981, 2931, 2845, 2680, 2633, 2606, 2485, 2441, 2361, 2316, 2185, 2111, 2059, 1920, 1742, 1710, 1607, 1582, 1491, 1465, 1437, 1392, 1370, 1339, 1293, 1259, 1244, 1208, 1144, 1132, 1098, 1052, 1025, 925, 904, 891, 866, 839, 797, 756, 729, 703, 653, 617, 601, 561, 547, 478, 462, 441; **HRMS** (ESI$^+$-QqTOF, *m/z*): calculated for $C_{15}H_{18}N_3O_4$ [M+H]$^+$: 304.1292, found: 304.1292; calculated for $C_{15}H_{21}N_4O_4$ [M+NH$_4$]$^+$: 321.1557, found: 321.1557 (major); calculated for $C_{15}H_{17}N_3O_4Na$ [M+Na]$^+$: 326.1111, found: 326.1111; calculated for $C_{15}H_{17}N_3O_4K$ [M+K]$^+$: 342.0851, found: 342.0852; **TLC** (silica gel K60, 200 µm, F254, heptanes/EtOAc = 7:3, 298 K, R_f / 1): 0.50; **MP** (uncorrected, ϑ_m / °C): < r.t.

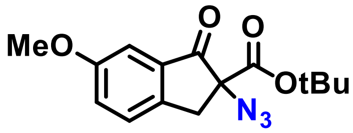

***tert*-Butyl 2-azido-6-methoxy-1-oxo-2,3-dihydro-1*H*-indene-2-carboxylate (2m):**[13,15] Obtained in 96% yield (23.7 mg, 78.0 µmol); yellow oil; **^{1}H-NMR** (500 MHz, $CDCl_3$, 298 K, δ / ppm): 7.33 (d, *J* = 8.4 Hz, 1H), 7.24 (dd, *J* = 8.4, 2.6 Hz, 1H), 7.21 (d, *J* = 2.6 Hz, 1H), 3.84 (s, 3H), 3.54 (d, *J* = 16.9 Hz, 1H), 2.90 (d, *J* = 16.8 Hz, 1H), 1.44 (s, 9H); **^{13}C-NMR** (126 MHz, $CDCl_3$, 298 K, δ / ppm): 198.0, 167.4, 160.0, 145.3, 134.5, 127.1, 125.8, 106.4, 84.5, 71.3, 55.8, 38.0, 27.9; **IR** (neat, FT-ATR, 298 K, $\tilde{\nu}$ / cm^{-1}): 3403, 3299, 3067, 2993, 2972, 2852, 2727, 2680, 2622, 2576, 2445, 2422, 2359, 2272, 2110, 2022, 1912, 1735, 1711, 1613, 1585, 1547, 1492, 1460, 1432, 1395, 1370, 1341, 1304, 1277, 1234, 1211, 1190, 1157, 1128, 1092, 1056, 1033, 1022, 959, 895, 876, 841, 824, 801, 787, 772, 739, 690, 634, 589, 555, 539, 524, 480, 463, 450, 435, 423; **HRMS** (ESI$^+$-QqTOF, *m/z*): calculated for $C_{15}H_{18}N_3O_4$ [M+H]$^+$: 304.1292, found: 304.1291; calculated for $C_{15}H_{21}N_4O_4$ [M+NH$_4$]$^+$: 321.1557, found: 321.1557 (major); calculated for $C_{15}H_{17}N_3O_4Na$ [M+Na]$^+$: 326.1111, found: 326.1111; calculated for $C_{15}H_{17}N_3O_4K$ [M+K]$^+$: 342.0851, found: 342.0851; **TLC** (silica gel K60, 200 µm, F254, heptanes/EtOAc = 7:3, 298 K, R_f / 1): 0.60; **MP** (uncorrected, ϑ_m / °C): < r.t.

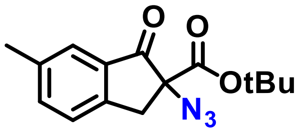

***tert*-Butyl 2-azido-6-methyl-1-oxo-2,3-dihydro-1*H*-indene-2-carboxylate (2n):**[13] Obtained in 78% yield (27.6 mg, 96.0 µmol); yellow oil; **^{1}H-NMR** (500 MHz, $CDCl_3$, 298 K, δ / ppm): 7.59 (s, 1H), 7.47 (d, J = 7.8 Hz, 1H), 7.33 (d, J = 7.8 Hz, 1H), 3.57 (d, J = 17.1 Hz, 1H), 2.93 (d, J = 17.1 Hz, 1H), 2.41 (s, 3H), 1.44 (s, 9H); **^{13}C-NMR** (126 MHz, $CDCl_3$, 298 K, δ / ppm): 198.1, 167.5, 149.8, 138.5, 137.6, 133.4, 126.1, 125.4, 84.5, 70.9, 38.3, 27.9, 21.2; **IR** (neat, FT-ATR, 298 K, $\bar{\nu}$ / cm^{-1}): 2980, 2932, 2870, 2358, 2325, 2286, 2110, 1742, 1715, 1617, 1584, 1494, 1457, 1424, 1395, 1276, 1241, 1202, 1147, 1102, 1047, 1102, 1047, 1102, 1046, 1036, 950, 905, 889, 839, 825, 798, 789, 748, 731, 695, 626, 584, 551, 503, 476, 453, 439, 417; **HRMS** (ESI$^+$-QqTOF, *m*/*z*): calculated for $C_{15}H_{18}N_3O_3$ $[M+H]^+$: 288.1343, found: 288.1343; calculated for $C_{15}H_{21}N_4O_3$ $[M+NH_4]^+$: 305.1608, found: 305.1608 (major); calculated for $C_{15}H_{17}N_3O_3Na$ $[M+Na]^+$: 310.1162, found: 310.1163; calculated for $C_{15}H_{17}N_3O_3K$ $[M+K]^+$: 326.0901, found: 326.0907; **TLC** (silica gel K60, 200 µm, F254, heptanes/EtOAc = 7:3, 298 K, R_f / 1): 0.67; **MP** (uncorrected, ϑ_m / °C): < r.t.

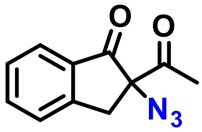

2-Acetyl-2-azido-2,3-dihydro-1*H*-inden-1-one (5a): Obtained in 61% yield (13.1 mg, 61.0 µmol); yellow oil; **^{1}H-NMR** (700 MHz, $CDCl_3$, 298 K, δ / ppm): 7.82 (d, J = 7.7 Hz, 1H), 7.70 (t, J = 7.5 Hz, 1H), 7.52 (d, J = 7.9 Hz, 1H), 7.47 (t, J = 7.5 Hz, 1H), 3.71 (d, J = 17.4 Hz, 1H), 3.08 (d, J = 17.4 Hz, 1H), 2.29 (s, 3H); **^{13}C-NMR** (176 MHz, $CDCl_3$, 298 K, δ / ppm): 200.8, 197.8, 152.2, 136.8, 133.5, 128.8, 126.7, 125.7, 77.8, 37.1, 26.1; **IR** (neat, FT-ATR, 298 K, $\bar{\nu}$ / cm^{-1}): 3412, 3077, 2954, 2926, 2854, 2362, 2342, 2324, 2100, 1726, 1708, 1606, 1588, 1465, 1424, 1356, 1334, 1301, 1256, 1211, 1188, 1154, 1090, 1053, 1025, 982, 959, 901, 877, 813, 778, 739, 704, 671, 644, 583, 564, 549, 524, 502, 468, 426; **HRMS** (ESI-QqTOF, *m*/*z*): calculated for $C_{11}H_9N_3O_2$ $[M+H]^+$: 216.0768, found: 216.0770; calculated for $C_{11}H_{13}N_4O_2$ $[M+NH_4]^+$: 233.1033, found: 233.1033 (major); calculated for $C_{12}H_{10}N_3O_4$ $[M+HCOO]^-$: 260.0677, found: 260.0684; **TLC** (silica gel K60, 200 µm, F254, heptanes/EtOAc = 7:3, 298 K, R_f / 1): 0.43; **MP** (uncorrected, ϑ_m / °C): < r.t.

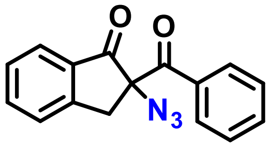

2-Azido-2-benzoyl-2,3-dihydro-1*H*-inden-1-one (5b): Obtained in 83% yield (23.0 mg, 83.0 µmol); yellow oil; **^{1}H-NMR** (700 MHz, $CDCl_3$, 298 K, δ / ppm): 7.92 (d, J = 7.7 Hz, 1H), 7.85 (d, J = 8.2 Hz, 2H), 7.72 (d, J = 7.4 Hz, 1H), 7.57 (t, J = 7.4 Hz, 1H), 7.52 (t, J = 7.6 Hz, 1H), 7.49 (d, J = 7.6 Hz, 1H), 7.41 (t, J = 7.8 Hz, 2H), 3.77 (d, J = 17.5 Hz, 1H), 3.25 (d, J = 17.5 Hz, 1H); **^{13}C-NMR** (176 MHz, $CDCl_3$, 298 K, δ / ppm): 197.9, 194.5, 151.2, 136.6, 134.0, 133.9, 133.5, 129.2, 128.9, 128.9, 126.9, 126.0, 76.3, 39.2; **IR** (neat, FT-ATR, 298 K, $\bar{\nu}$ / cm^{-1}): 3068, 2925, 2853, 2449, 2280, 2099, 1786, 1763, 1716, 1681, 1598, 1465, 1448, 1429, 1303, 1257, 1234, 1213, 1155, 1089, 1032, 994, 961, 946, 899, 871, 846, 799, 777, 741, 701, 688, 671, 610, 550, 468, 443, 419; **HRMS** (ESI-QqTOF, *m*/*z*): calculated for $C_{16}H_{12}N_3O_2$ $[M+H]^+$: 278.0924, found: 278.0924; calculated for $C_{16}H_{15}N_4O_2$ $[M+NH_4]^+$: 295.1190, found: 295.1190 (major); calculated for $C_{16}H_{11}N_3O_2Na$ $[M+Na]^+$: 300.0743, found: 300.0743; calculated for $C_{16}H_{11}N_3O_2K$ $[M+K]^+$: 316.0483, found: 316.0483; calculated for $C_{17}H_{12}N_3O_4$ $[M+HCOO]^-$: 322.0833, found: 322.0833; **TLC** (silica gel K60, 200 µm, F254, heptanes/EtOAc = 7:3, 298 K, R_f / 1): 0.48; **MP** (uncorrected, ϑ_m / °C): < r.t.

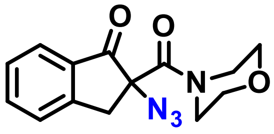

2-Azido-2-(morpholin-4-ylcarbonyl)-2,3-dihydro-1*H*-inden-1-one (6): Obtained in 78% yield (22.3 mg, 78.0 µmol); yellow solid; **^{1}H-NMR** (700 MHz, $CDCl_3$, 298 K, δ / ppm): 7.84 (d, J = 7.4 Hz, 1H), 7.67 (t, J = 7.5 Hz, 1H), 7.48 - 7.43 (m, 2H), 3.81 - 3.54 (m, 9H), 3.17 (d, J = 16.9 Hz, 1H); **^{13}C-NMR** (176 MHz, $CDCl_3$, 298 K, δ / ppm): 197.5, 165.8, 150.3, 136.5, 133.6, 129.2, 128.8, 126.6, 125.7, 72.4, 66.9, 38.6; **IR** (neat, FT-ATR, 298 K, $\bar{\nu}$ / cm^{-1}): 2964, 2922, 2856, 2360, 2249, 2099, 1718, 1638, 1607, 1558, 1457, 1425, 1361, 1330, 1301, 1270, 1255, 1233, 1213, 1184, 1155, 1113, 1090, 1065, 1010, 942, 914, 873, 852, 836, 808, 795, 759, 732, 703, 674, 646, 626, 582, 550, 534, 478, 454, 418; **HRMS** (ESI^+-QqTOF, *m*/*z*): calculated for $C_{14}H_{15}N_4O_3$ $[M+H]^+$: 287.1139, found: 287.1139 (major); calculated for $C_{14}H_{14}N_4O_3Na$ $[M+Na]^+$: 309.0958, found: 309.0958; calculated for $C_{14}H_{14}N_4O_3K$ $[M+K]^+$: 325.0697, found: 325.0694; **TLC** (silica gel K60, 200 µm, F254, heptanes/IPA = 7:3, 298 K, R_f / 1): 0.36; **MP** (uncorrected, ϑ_m / °C): 125.0 - 127.0.

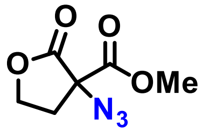

Methyl 3-azido-2-oxotetrahydrofuran-3-carboxylate (7a): Obtained in 55% yield (10.2 mg, 55.0 µmol); yellowish oil; ^{1}**H-NMR** (700 MHz, $CDCl_3$, 298 K, δ / ppm): 4.45 (2 × ddd, J = 8.9, 7.7, 6.1 Hz, 2H), 3.91 (s, 3H), 2.77 (ddd, J = 13.3, 7.6, 5.5 Hz, 1H), 2.29 (ddd, J = 13.6, 7.9, 6.6 Hz, 1H); ^{13}**C-NMR** (176 MHz, $CDCl_3$, 298 K, δ / ppm): 170.7, 167.4, 66.6, 66.5, 54.2, 33.6; **IR** (neat, FT-ATR, 298 K, $\bar{\nu}$ / cm^{-1}): 2960, 2925, 2852, 2360, 2114, 1775, 1748, 1669, 1600, 1559, 1483, 1451, 1437, 1380, 1318, 1245, 1216, 1171, 1118, 1019, 996, 955, 918, 841, 803, 791, 745, 705, 687, 634, 615, 601, 552, 536, 469, 455, 434; **HRMS** (ESI$^+$-QqTOF, m/z): calculated for $C_6H_8N_3O_4$ $[M+H]^+$: 186.0509, found: 186.0508; calculated for $C_6H_8N_3O_4$ $[M+NH_4]^+$: 203.0775, found: 203.0776 (major); **TLC** (silica gel K60, 200 µm, F254, heptanes/IPA = 7:3, 298 K, R_f / 1): 0.34; **MP** (uncorrected, ϑ_m / °C): < r.t.

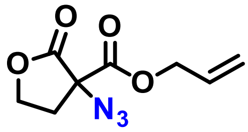

Allyl 3-azido-2-oxotetrahydrofuran-3-carboxylate (7b): Obtained in 74% yield (15.6 mg, 74.0 µmol); yellowish oil; ^{1}**H-NMR** (700 MHz, $CDCl_3$, 298 K, δ / ppm): 5.93 (ddt, J = 16.6, 10.3, 5.8 Hz, 1H), 5.39 (dd, J = 17.1, 1.4 Hz, 1H), 5.33 (dd, J = 10.4, 1.3 Hz, 1H), 4.85 - 4.74 (m, 2H), 4.53 - 4.40 (m, 2H), 2.78 (ddd, J = 13.3, 7.6, 5.4 Hz, 1H), 2.30 (ddd, J = 13.5, 8.0, 6.6 Hz, 1H).; ^{13}**C-NMR** (176 MHz, $CDCl_3$, 298 K, δ / ppm): 170.7, 166.6, 130.5, 120.3, 67.9, 66.6, 66.5, 33.6; **IR** (neat, FT-ATR, 298 K, $\bar{\nu}$ / cm^{-1}): 3090, 2990, 2956, 2928, 2862, 2115, 1775, 1747, 1650, 1483, 1447, 1424, 1379, 1316, 1270, 1241, 1211, 1171, 1117, 1020, 975, 937, 843, 790, 740, 714, 688, 661, 640, 597, 551, 535; **HRMS** (ESI$^+$-QqTOF, m/z): calculated for $C_8H_9N_3O_4$ $[M+H]^+$: 212.0666, found: 212.0666; calculated for $C_8H_{13}N_4O_4$ $[M+NH_4]^+$: 229.0931, found: 229.0931 (major); calculated for $C_8H_9N_3O_4Na$ $[M+Na]^+$: 234.0485, found: 234.0488; calculated for $C_8H_9N_3O_4K$ $[M+K]^+$: 250.0225, found: 250.0223; **TLC** (silica gel K60, 200 µm, F254, heptanes/IPA = 7:3, 298 K, R_f / 1): 0.57; **MP** (uncorrected, ϑ_m / °C): < r.t.

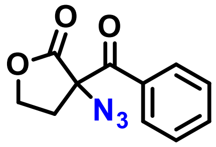

3-Azido-3-benzoyldihydrofuran-2(3*H*)-one (7c):[18] Obtained in 69% yield (16.0 mg, 69.0 µmol); yellowish oil; **^{1}H-NMR** (700 MHz, $CDCl_3$, 298 K, δ / ppm): 5.93 (ddt, J = 16.6, 10.3, 5.8 Hz, 1H), 5.39 (dd, J = 17.1, 1.4 Hz, 1H), 5.33 (dd, J = 10.4, 1.3 Hz, 1H), 4.85 - 4.74 (m, 2H), 4.53 - 4.40 (m, 2H), 2.78 (ddd, J = 13.3, 7.6, 5.4 Hz, 1H), 2.30 (ddd, J = 13.5, 8.0, 6.6 Hz, 1H).; **^{13}C-NMR** (176 MHz, $CDCl_3$, 298 K, δ / ppm): 170.7, 166.6, 130.5, 120.3, 67.9, 66.6, 66.5, 33.6; **IR** (neat, FT-ATR, 298 K, $\bar{\nu}$ / cm^{-1}): 3067, 2983, 2923, 2858, 2355, 2107, 1771, 1673, 1596, 1579, 1482, 1449, 1377, 1308, 1272, 1243, 1214, 1176, 1102, 1021, 1001, 953, 886, 844, 796, 760, 718, 686, 650, 616, 579, 540, 499, 418; **HRMS** (ESI^+-QqTOF, *m*/*z*): calculated for $C_{11}H_{10}N_3O_3$ $[M+H]^+$: 232.0717, found: 232.0717; calculated for $C_{11}H_{13}N_4O_3$ $[M+NH_4]^+$: 249.0982, found: 287.0982 (major); calculated for $C_{11}H_9N_3O_3Na$ $[M+Na]^+$: 254.0536, found: 254.0536; calculated for $C_{11}H_9N_3O_3K$ $[M+K]^+$: 270.0275, found: 270.0276; **TLC** (silica gel K60, 200 µm, F254, heptanes/IPA = 7:3, 298 K, R_f / 1): 0.51; **MP** (uncorrected, ϑ_m / °C): < r.t.

5. Oxidative α-nitration reactions

General procedure

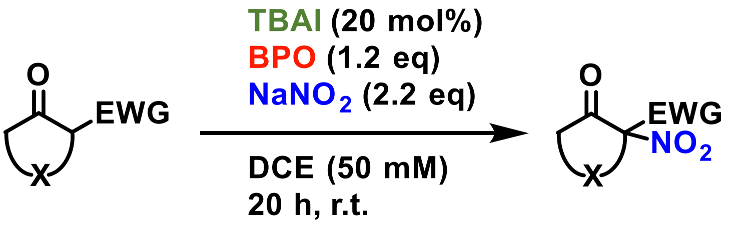

Sodium azide (7.9 mg, 120 µmol, 1.2 equiv) and TBAI (7.5 mg, 20 µmol, 20 mol %) were suspended in a stirred solution (900–1000 rpm) of the respective substrate (100 µmol, 1.00 equiv) in 1.0 mL of DCE at rt. Then, a solution of anhydrous benzoyl peroxide (29.1 mg, 120 µmol, 1.2 equiv) in 1.0 mL of DCE was added to the suspension and stirred for 20 h.

The reaction solution was then diluted with 8 mL dichloromethane and extracted with 5 mL of sat. aq $NaHCO_3$. The aqueous phase was then extracted twice with 10 mL of DCM. The organic layer and the extracts were then filtered consecutively through a pad of anhydr. sodium sulfate and deactivated silica gel. The solvents were removed in vacuo. In most cases the products were already obtained in high purity (>95 %) after this work up. If necessary, further purification can be achieved by silica gel column chromatography.

Characterization of the α-nitration products

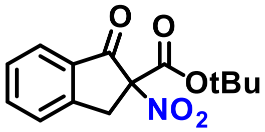

***tert*-Butyl 2-nitro-1-oxo-2,3-dihydro-1*H*-indene-2-carboxylate (10a):** Obtained in 84% yield (23.3 mg, 94.0 µmol); white solid; **^{1}H-NMR** (700 MHz, $CDCl_3$, 298 K, δ / ppm): 7.86 (d, J = 7.7 Hz, 1H), 7.71 (t, J = 7.5 Hz, 1H), 7.53 (d, J = 7.7 Hz, 1H), 7.48 (t, J = 7.5 Hz, 1H), 4.11 (d, J = 17.9 Hz, 1H), 3.99 (d, J = 17.9 Hz, 1H), 1.49 (s, 9H); **^{13}C-NMR** (126 MHz, $CDCl_3$, 298 K, δ / ppm): 188.4, 162.0, 150.1, 137.0, 132.9, 129.1, 126.5, 126.2, 96.7, 86.1, 37.5, 27.8.; **IR** (neat, FT-ATR, 298 K, $\bar{\nu}$ / cm^{-1}): 2984, 2930, 2878, 2854, 1748, 1719, 1656, 1604, 1589, 1548, 1465, 1431, 1396, 1371, 1353, 1325, 1272, 1260, 1215, 1145, 1091, 1056, 1026, 961, 912, 871, 844, 834, 818, 803, 755, 730, 711, 688, 661, 625, 598, 561, 533, 459, 414; **HRMS** (ESI$^+$-QqTOF, *m*/*z*): calculated for $C_{14}H_{16}NO_5$ $[M+H]^+$: 278.1023, found: 278.1024; calculated for $C_{14}H_{19}N_2O_5$ $[M+NH_4]^+$: 295.1288, found: 295.1288 (major); calculated for $C_{14}H_{15}NO_5Na$ $[M+Na]^+$: 300.0842, found: 300.0842; calculated for $C_{14}H_{15}NO_5K$ $[M+K]^+$: 316.0582, found: 316.0582; **TLC** (silica gel K60, 200 µm, F254, heptanes/EtOAc = 7:3, 298 K, R_f / 1): 0.47; **MP** (uncorrected, ϑ_m / °C): 75.9 – 78.4.

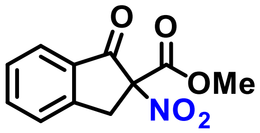

Methyl 2-nitro-1-oxo-2,3-dihydro-1*H*-indene-2-carboxylate (10b)[19]: Obtained in 86% yield (19.9 mg, 96.0 µmol); colourless oil; **^{1}H-NMR** (700 MHz, $CDCl_3$, 298 K, δ / ppm): 7.87 (d, J = 7.8 Hz, 1H), 7.73 (t, J = 7.5 Hz, 1H), 7.54 (d, J = 7.8 Hz, 1H), 7.50 (t, J = 7.6 Hz, 1H), 4.15 (d, J = 18.0 Hz, 1H), 4.05 (d, J = 18.0 Hz, 1H), 3.89 (s, 3H); **^{13}C-NMR** (126 MHz, $CDCl_3$, 298 K, δ / ppm): 188.0, 163.8, 150.0, 137.3, 132.7, 129.3, 126.5, 126.4, 96.1, 54.5, 37.5; **IR** (neat, FT-ATR, 298 K, $\tilde{\nu}$ / cm^{-1}): 2959, 1755, 1729, 1605, 1552, 1434, 1264, 1215, 1202, 1181, 1091, 1023, 949, 903, 894, 798, 761, 689, 663, 632, 467; **HRMS** (ESI^+-QqTOF, *m*/*z*): calculated for $C_{11}H_{10}NO_5$ $[M+H]^+$: 236.0553, found: 236.0553; calculated for $C_{11}H_{13}N_2O_5$ $[M+NH_4]^+$: 253.0820, found: 253.0820 (major); calculated for $C_{11}H_9NO_5Na$ $[M+Na]^+$: 258.0373, found: 258.0373; calculated for $C_{11}H_9NO_5K$ $[M+K]^+$: 274.0112, found: 274.0112; **TLC** (silica gel K60, 200 µm, F254, heptanes/EtOAc = 7:3, 298 K, R_f / 1): 0.29; **MP** (uncorrected, ϑ_m / °C): < r.t.

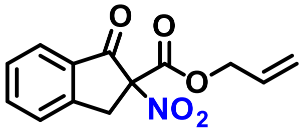

Allyl 2-nitro-1-oxo-2,3-dihydro-1*H*-indene-2-carboxylate (10c): Obtained in 75% yield (19.3 mg, 75.0 µmol); yellowish oil; **^{1}H-NMR** (700 MHz, $CDCl_3$, 298 K, δ / ppm): 7.88 (d, J = 7.7 Hz, 1H), 7.73 (t, J = 7.5 Hz, 1H), 7.54 (d, J = 7.7 Hz, 1H), 7.50 (t, J = 7.5 Hz, 1H), 5.89 (ddt, J = 16.5, 11.0, 5.7 Hz, 1H), 5.38 - 5.27 (m, 2H), 4.81 - 4.74 (m, 2H), 4.16 (d, J = 18.0 Hz, 1H), 4.05 (d, J = 18.0 Hz, 1H); **^{13}C-NMR** (126 MHz, $CDCl_3$, 298 K, δ / ppm): 188.0, 163.0, 150.0, 137.2, 132.7, 130.3, 129.3, 126.5, 126.5, 120.1, 96.1, 68.2, 37.5; **IR** (neat, FT-ATR, 298 K, $\tilde{\nu}$ / cm^{-1}): 3063, 2940, 2453, 2324, 1920, 1782, 1757, 1731, 1656, 1599, 1557, 1466, 1450, 1423, 1350, 1296, 1275, 1255, 1217, 1178, 1134, 1093, 1070, 1034, 1019, 995, 941, 926, 902, 840, 809, 794, 755, 692, 616, 601, 555, 500, 467, 437, 416; **HRMS** (ESI^+-QqTOF, *m*/*z*): calculated for $C_{13}H_{12}N_1O_5$ $[M+H]^+$: 262.0710, found: 262.0710; calculated for $C_{13}H_{15}N_2O_5$ $[M+NH_4]^+$: 279.0975, found: 279.0975 (major); calculated for $C_{13}H_{11}NO_5Na$ $[M+Na]^+$: 284.0529, found: 284.0529; calculated for $C_{13}H_{11}NO_5K$ $[M+K]^+$: 300.0269, found: 300.0272; **TLC** (silica gel K60, 200 µm, F254, heptanes/EtOAc = 7:3, 298 K, R_f / 1): 0.62; **MP** (uncorrected, ϑ_m / °C): < r.t.

6. NMR spectra

2a, ^{1}H-NMR (300 MHz, $CDCl_3$, 298 K, δ / ppm):

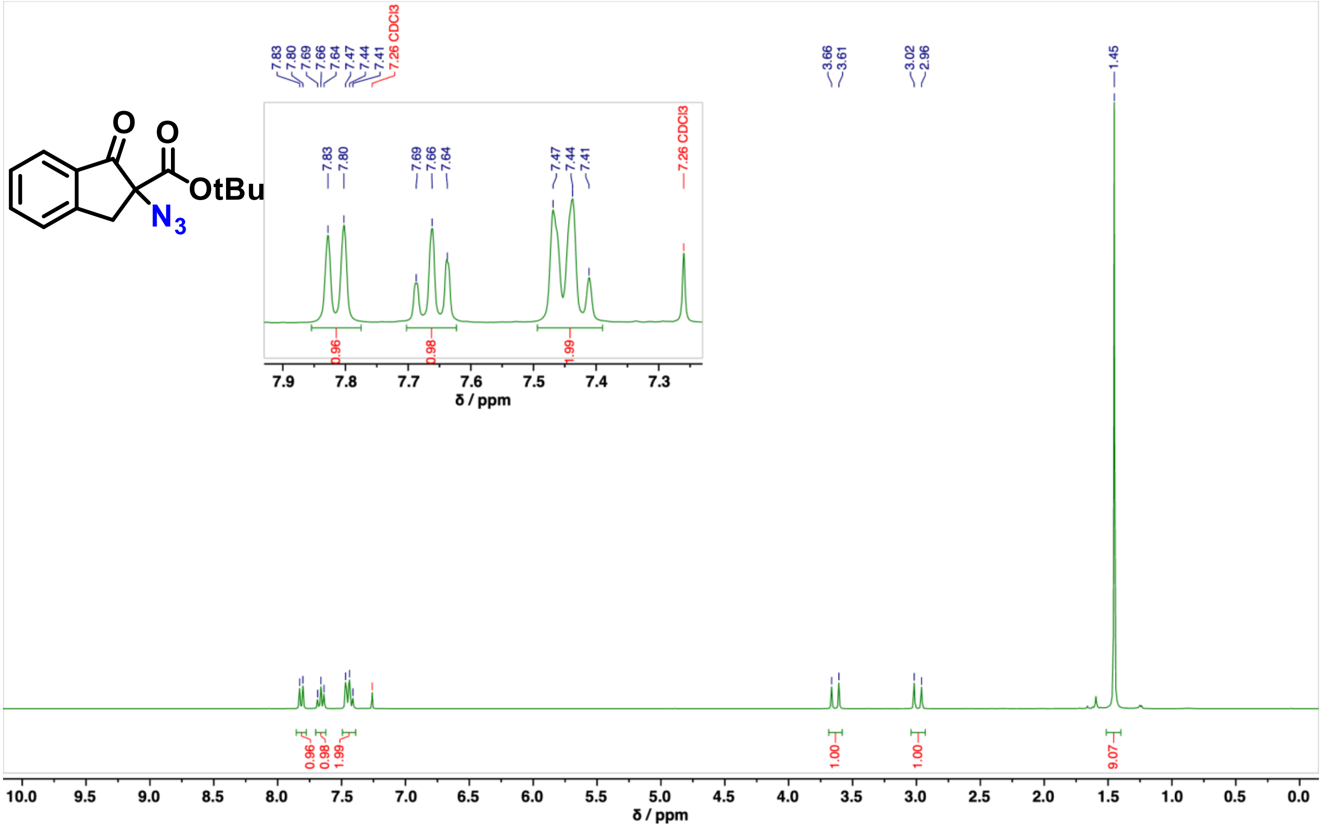

2a, ^{13}C-NMR (126 MHz, $CDCl_3$, 298 K, δ / ppm):

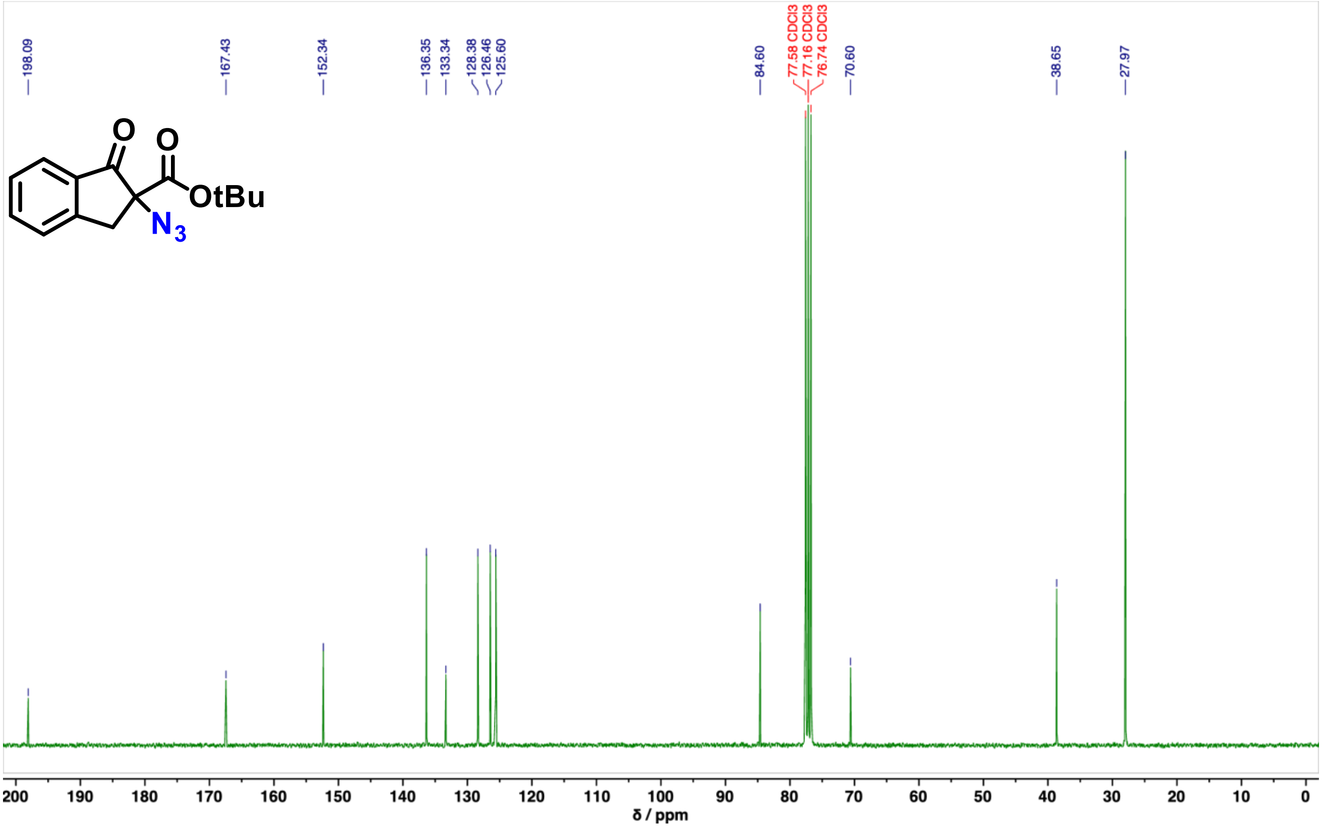

2b, ^{1}H-NMR (500 MHz, $CDCl_3$, 298 K, δ / ppm):

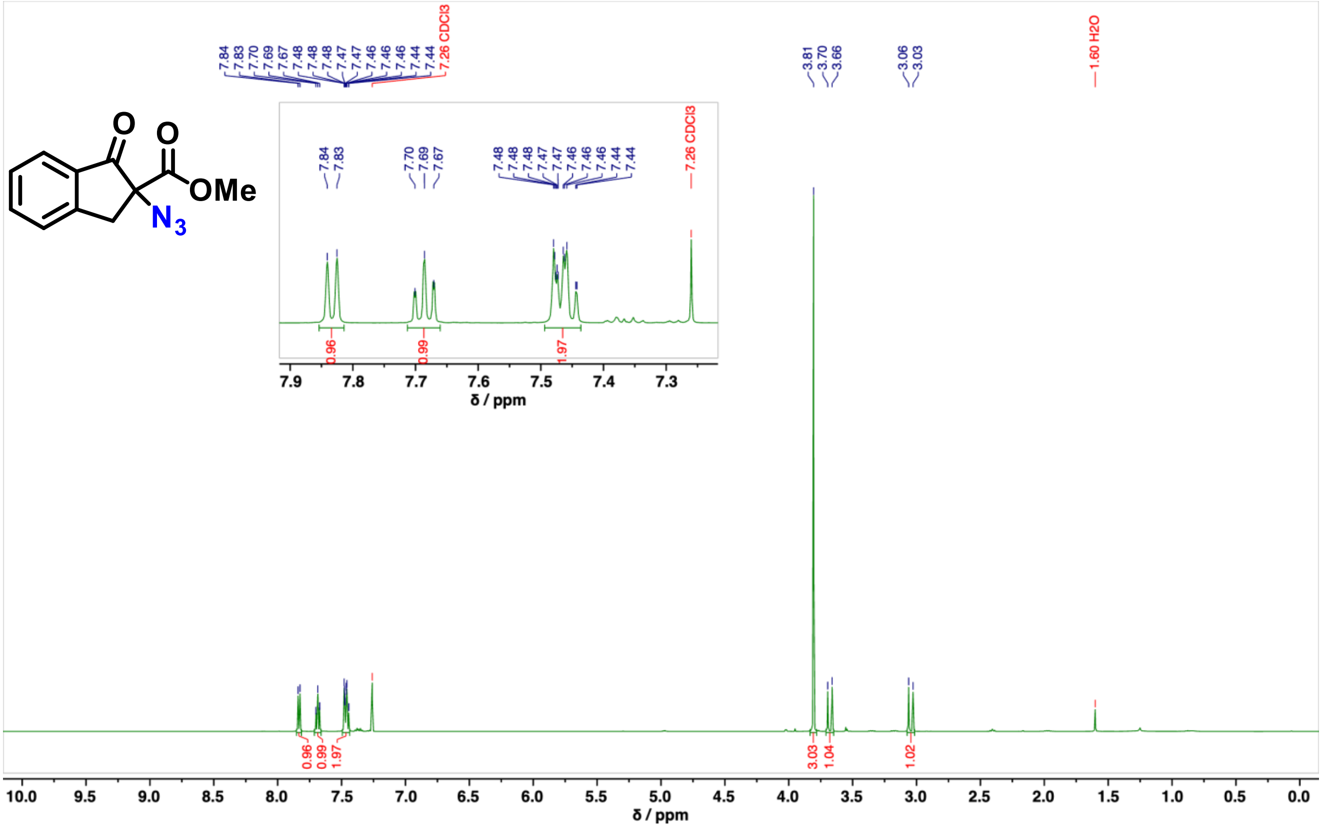

2b, ^{13}C-NMR (126 MHz, $CDCl_3$, 298 K, δ / ppm):

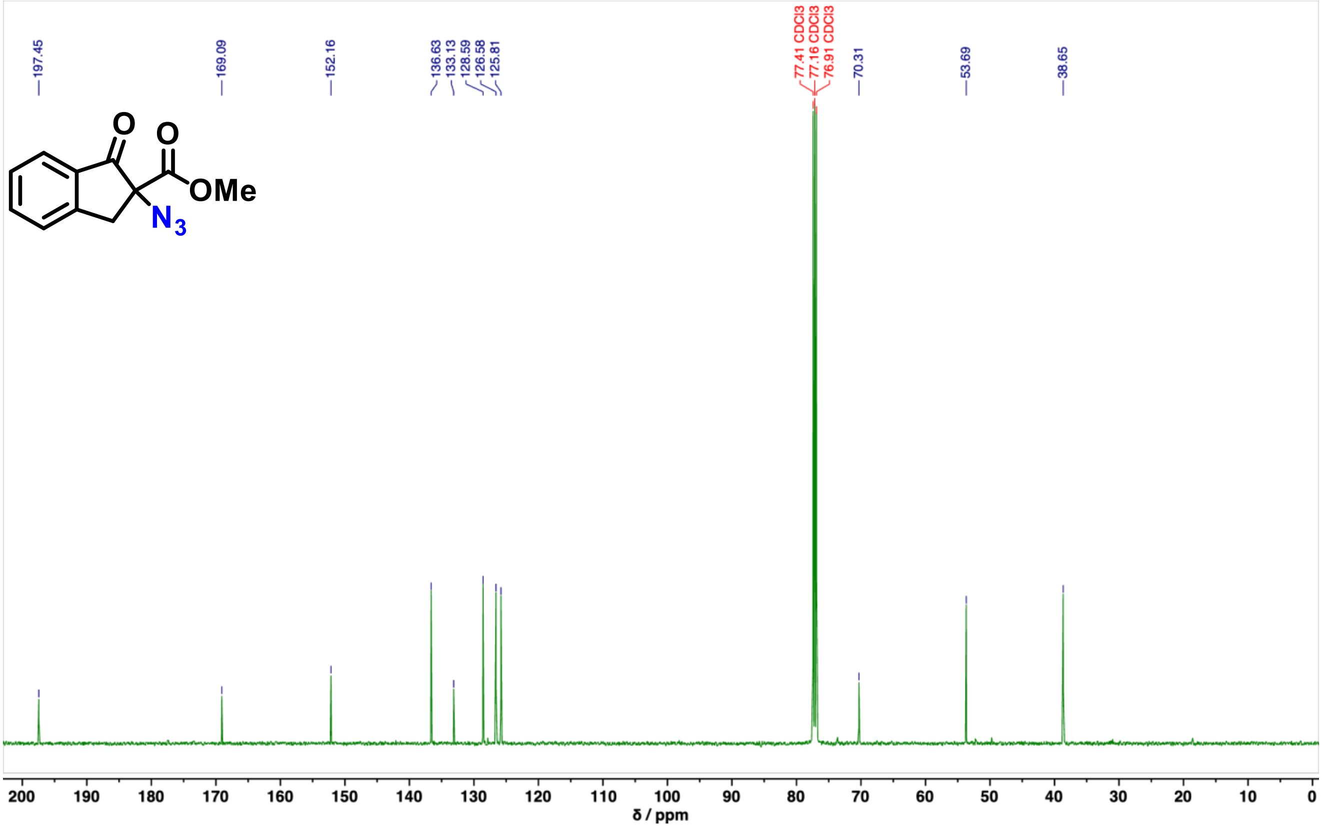

2c, ^{1}H-NMR (500 MHz, $CDCl_3$, 298 K, δ / ppm):

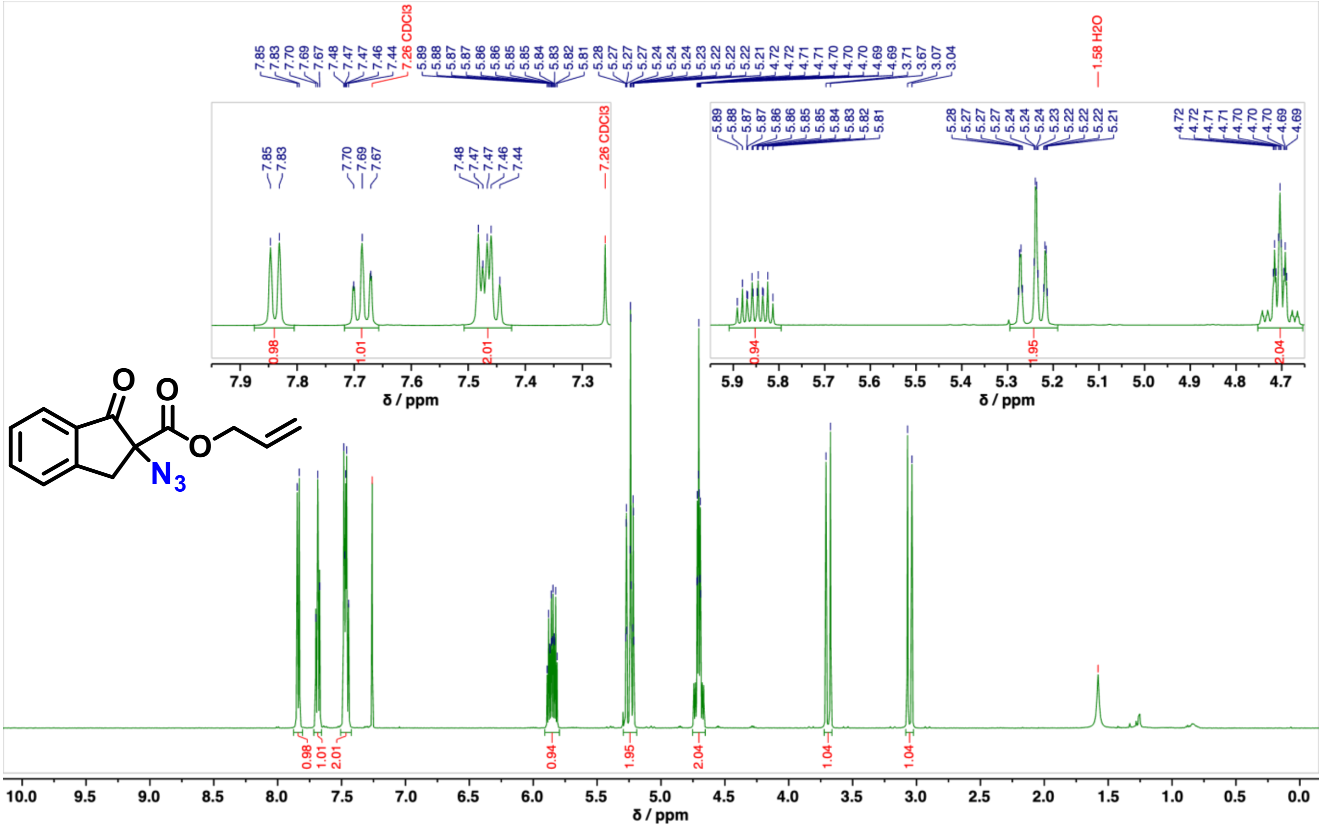

2c, ^{13}C-NMR (126 MHz, $CDCl_3$, 298 K, δ / ppm):

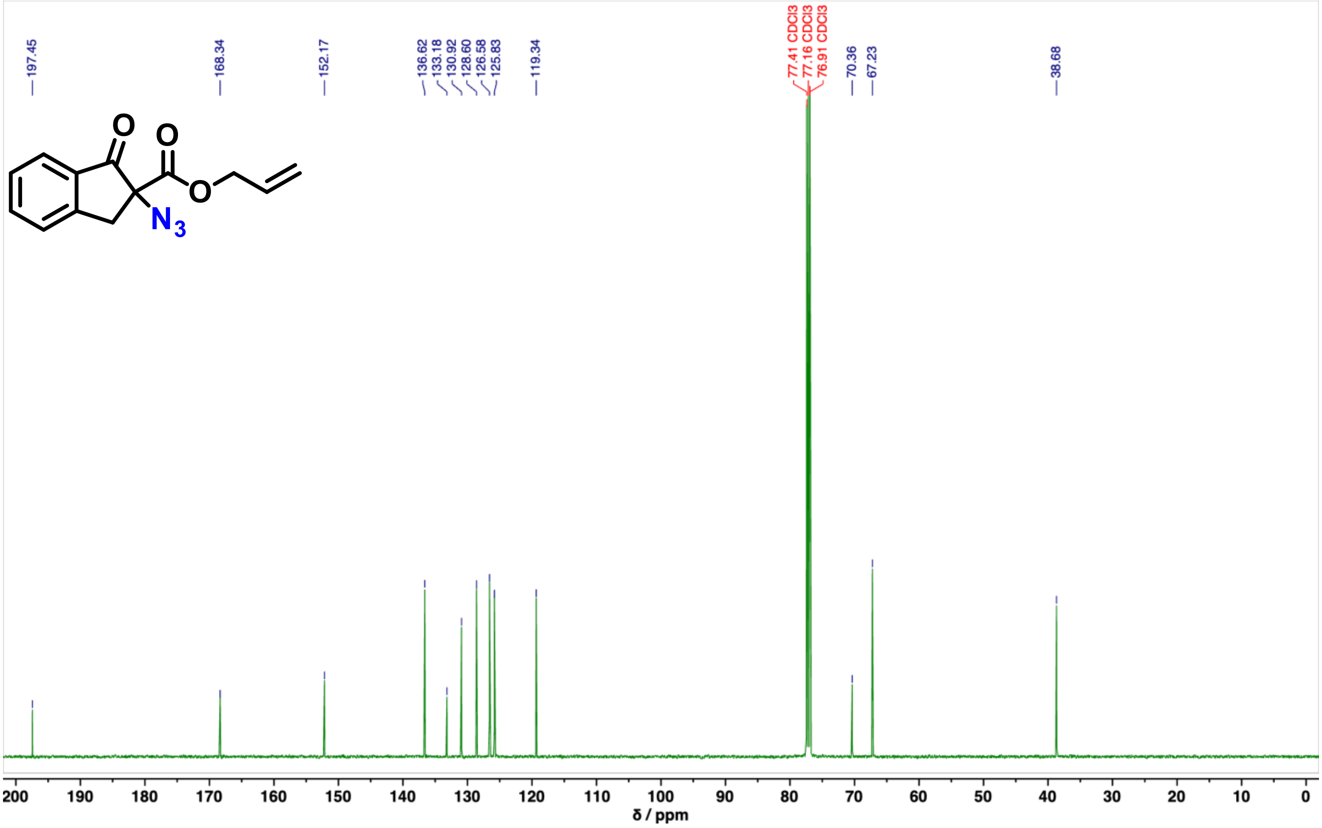

2d, ^{1}H-NMR (500 MHz, $CDCl_3$, 298 K, δ / ppm):

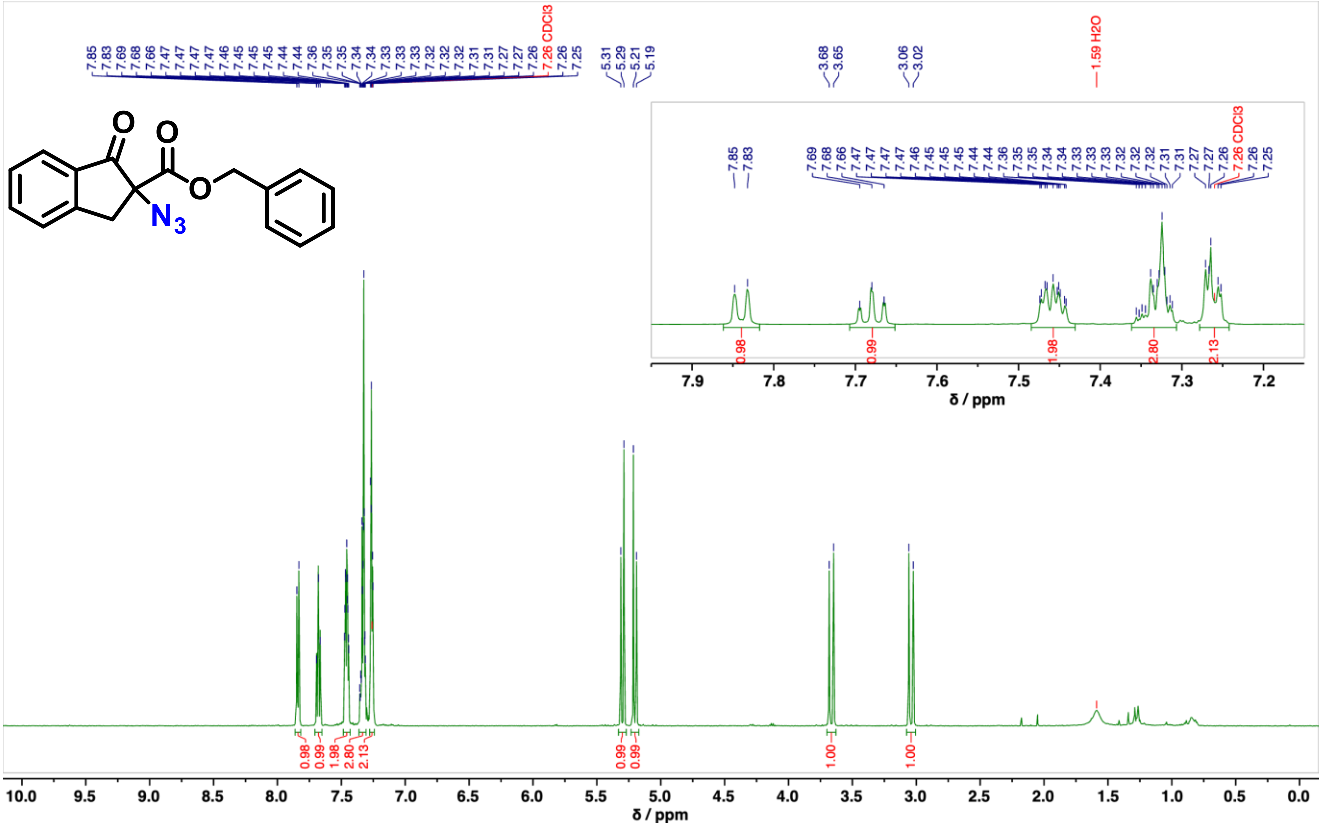

2d, ^{13}C-NMR (126 MHz, $CDCl_3$, 298 K, δ / ppm):

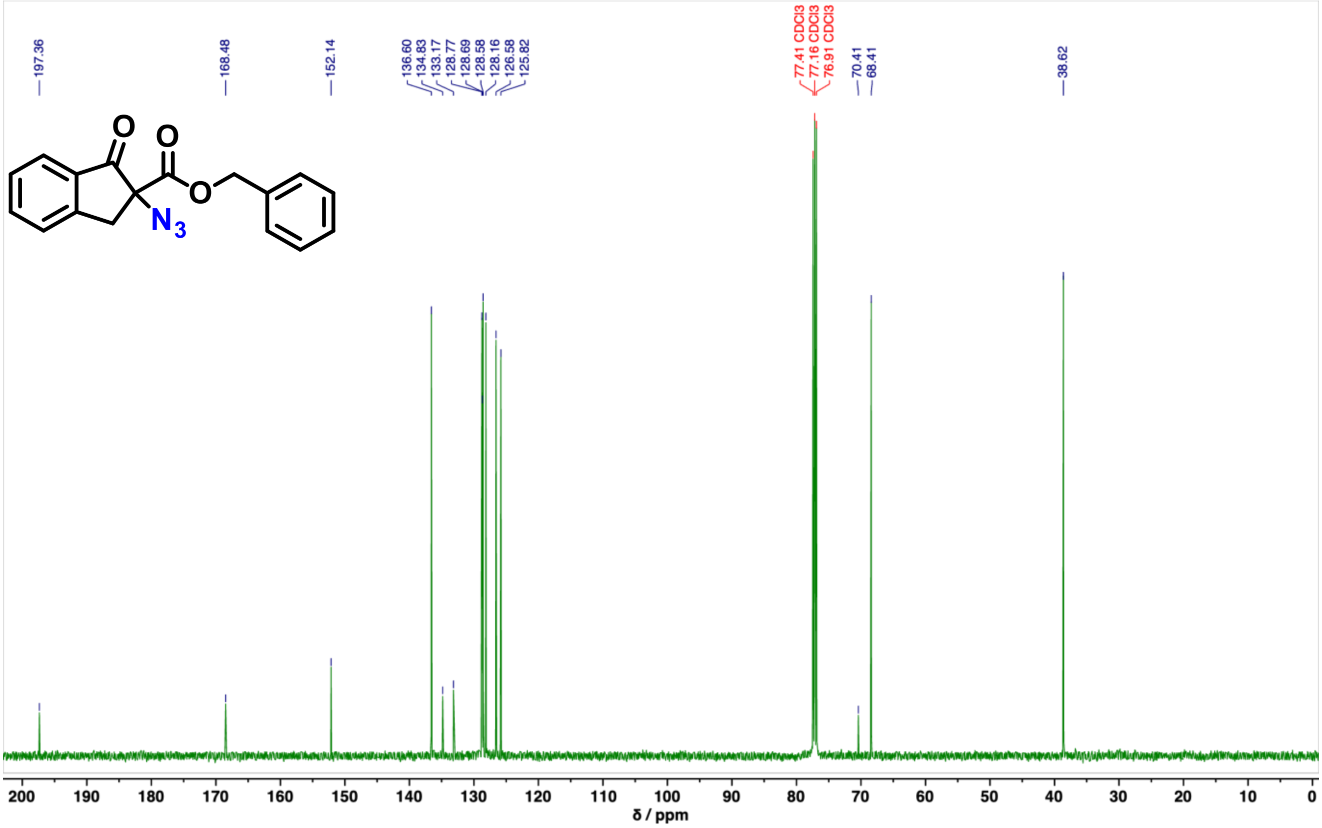

2e, ^{1}H-NMR (500 MHz, $CDCl_3$, 298 K, δ / ppm):

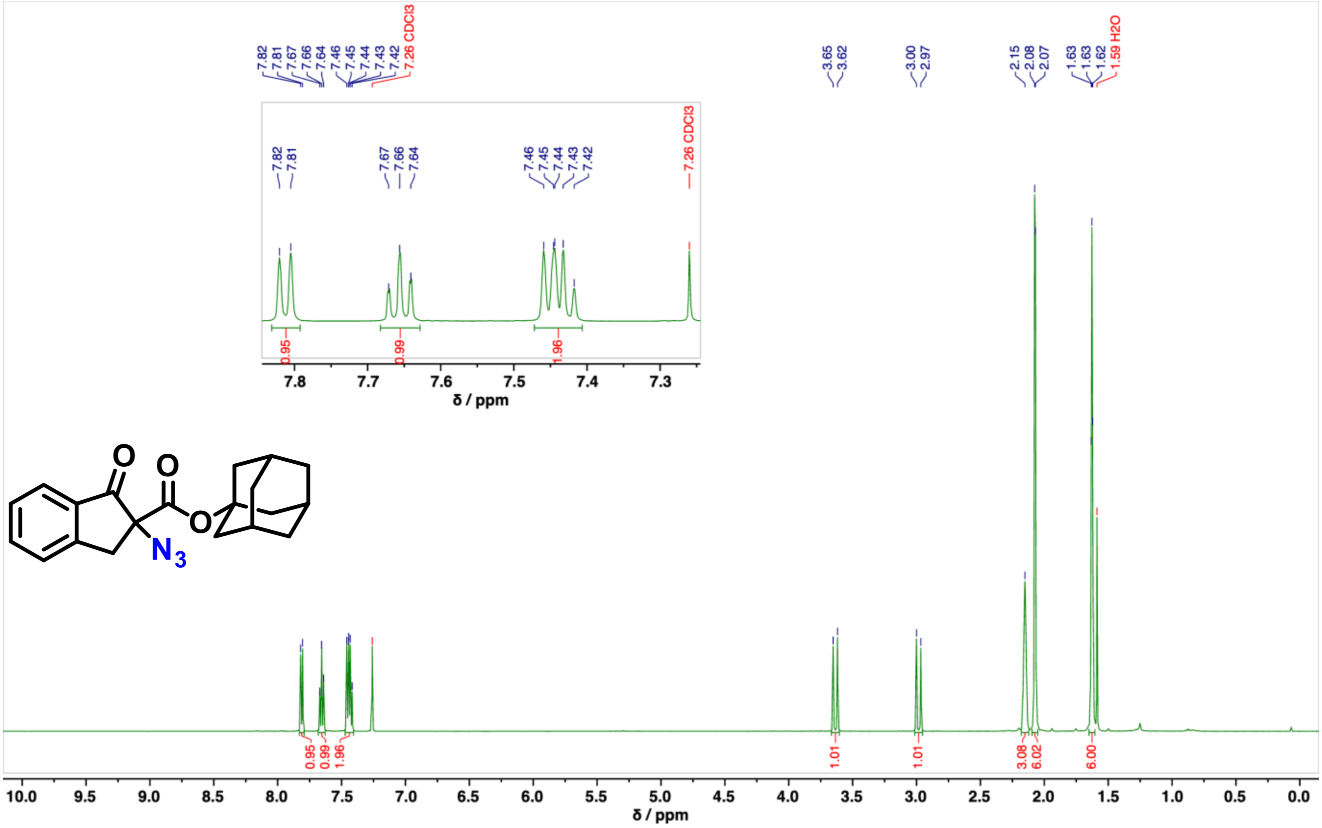

2e, ^{13}C-NMR (126 MHz, $CDCl_3$, 298 K, δ / ppm):

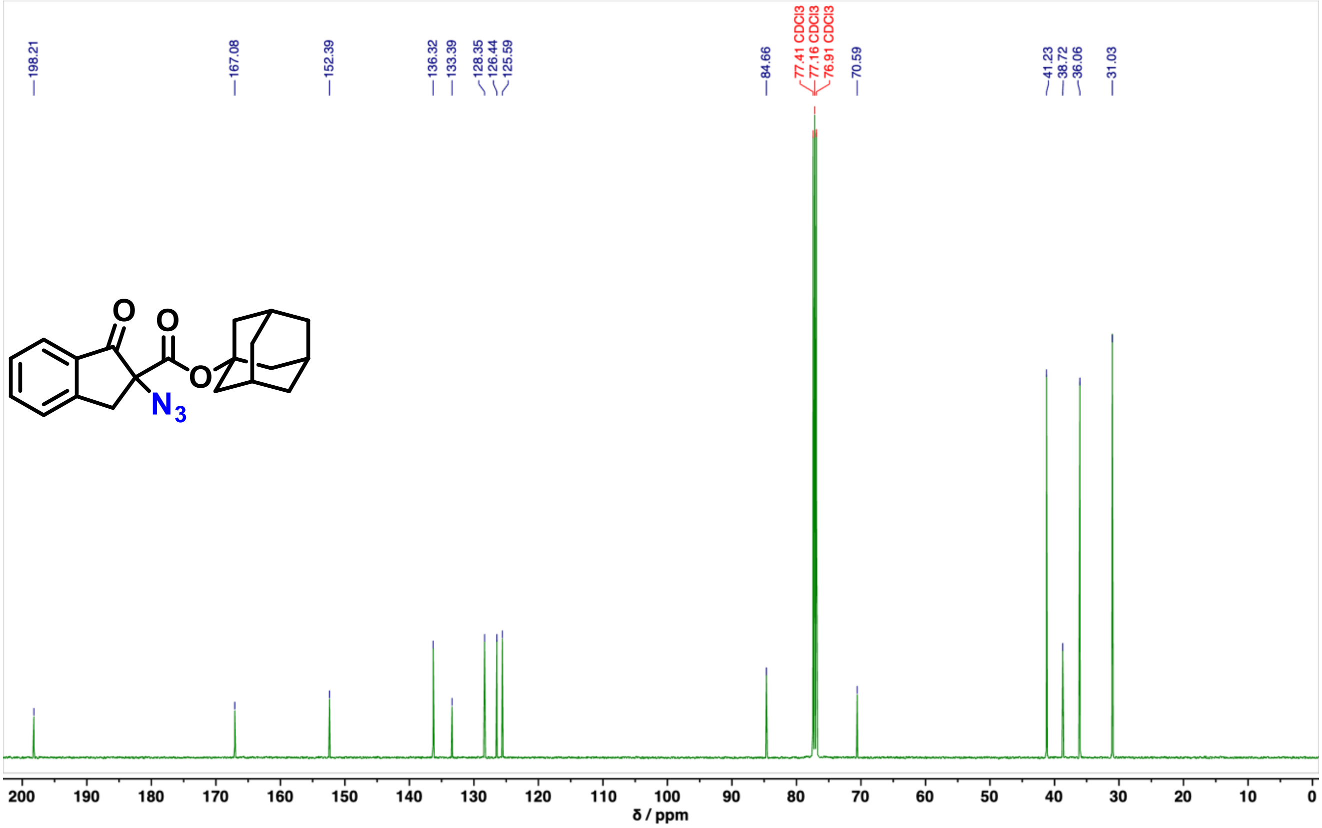

2f, ^{1}H-NMR (500 MHz, $CDCl_3$, 298 K, δ / ppm):

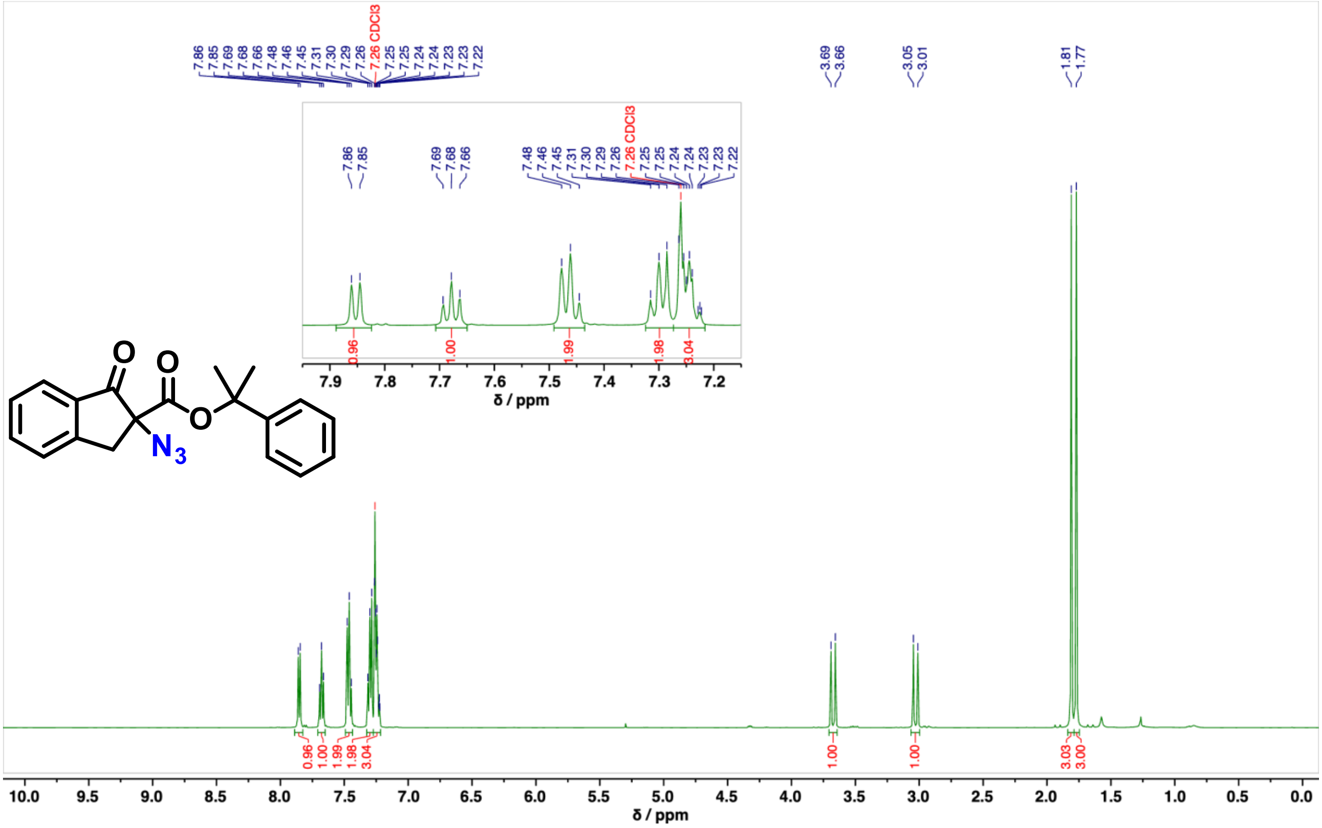

2f, ^{13}C-NMR (126 MHz, $CDCl_3$, 298 K, δ / ppm):

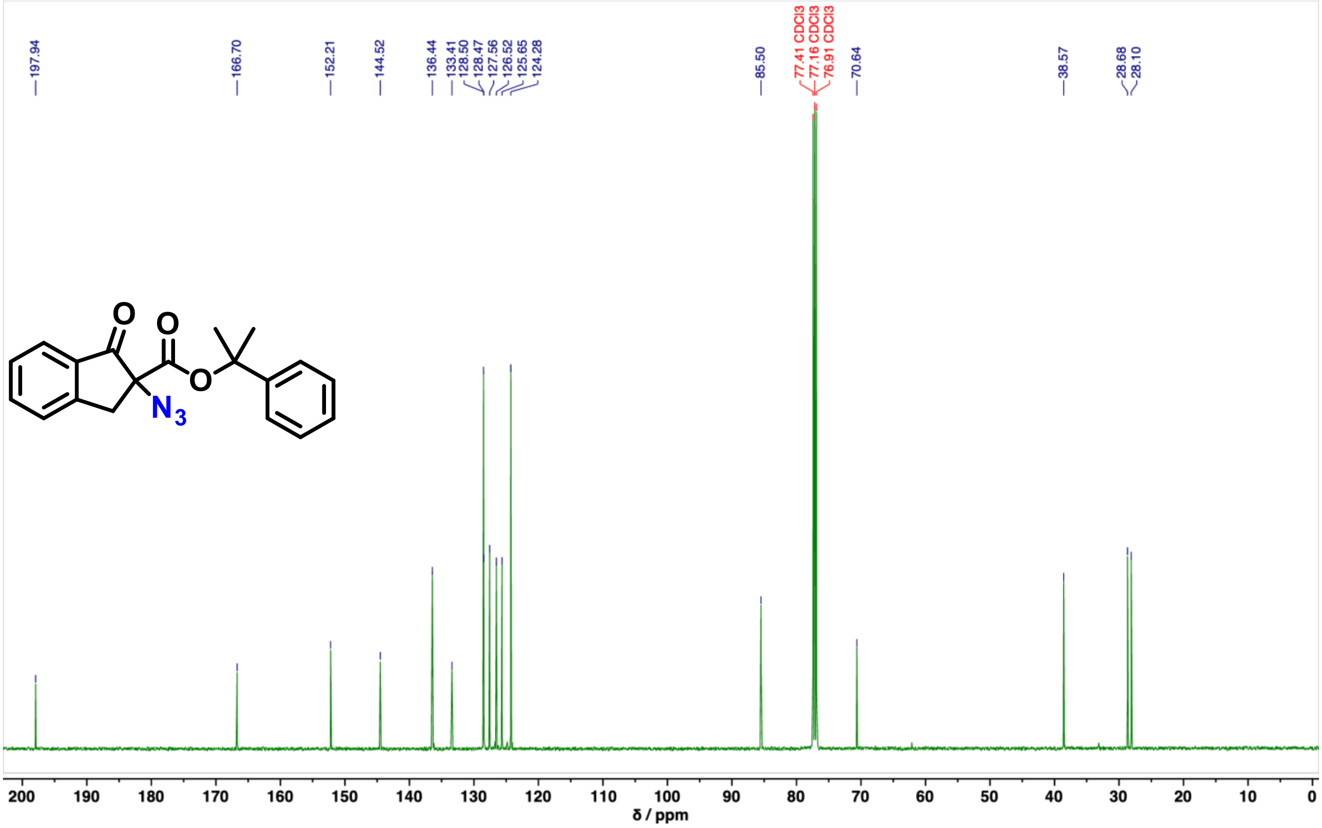

2g, ^{1}H-NMR (500 MHz, $CDCl_3$, 298 K, δ / ppm):

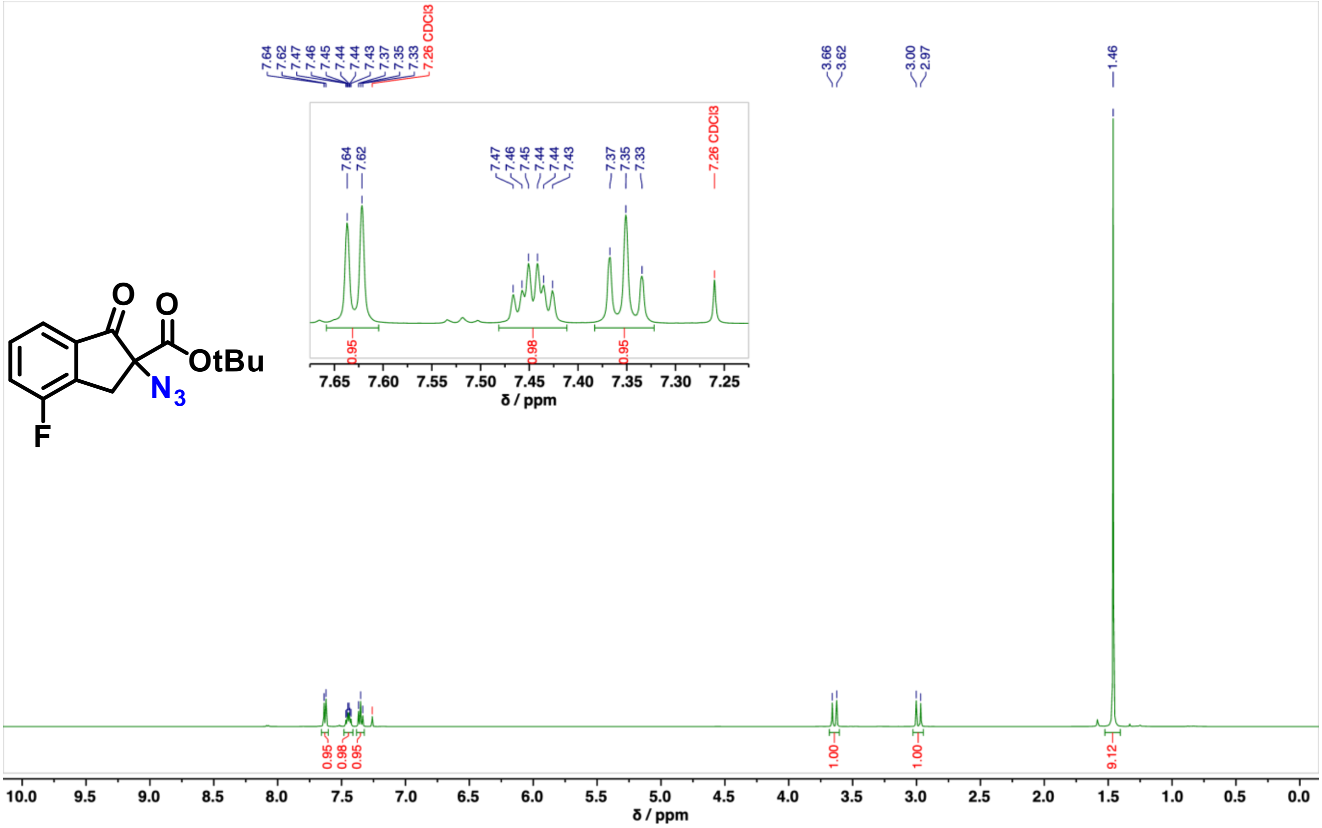

2g, ^{13}C-NMR (126 MHz, $CDCl_3$, 298 K, δ / ppm):

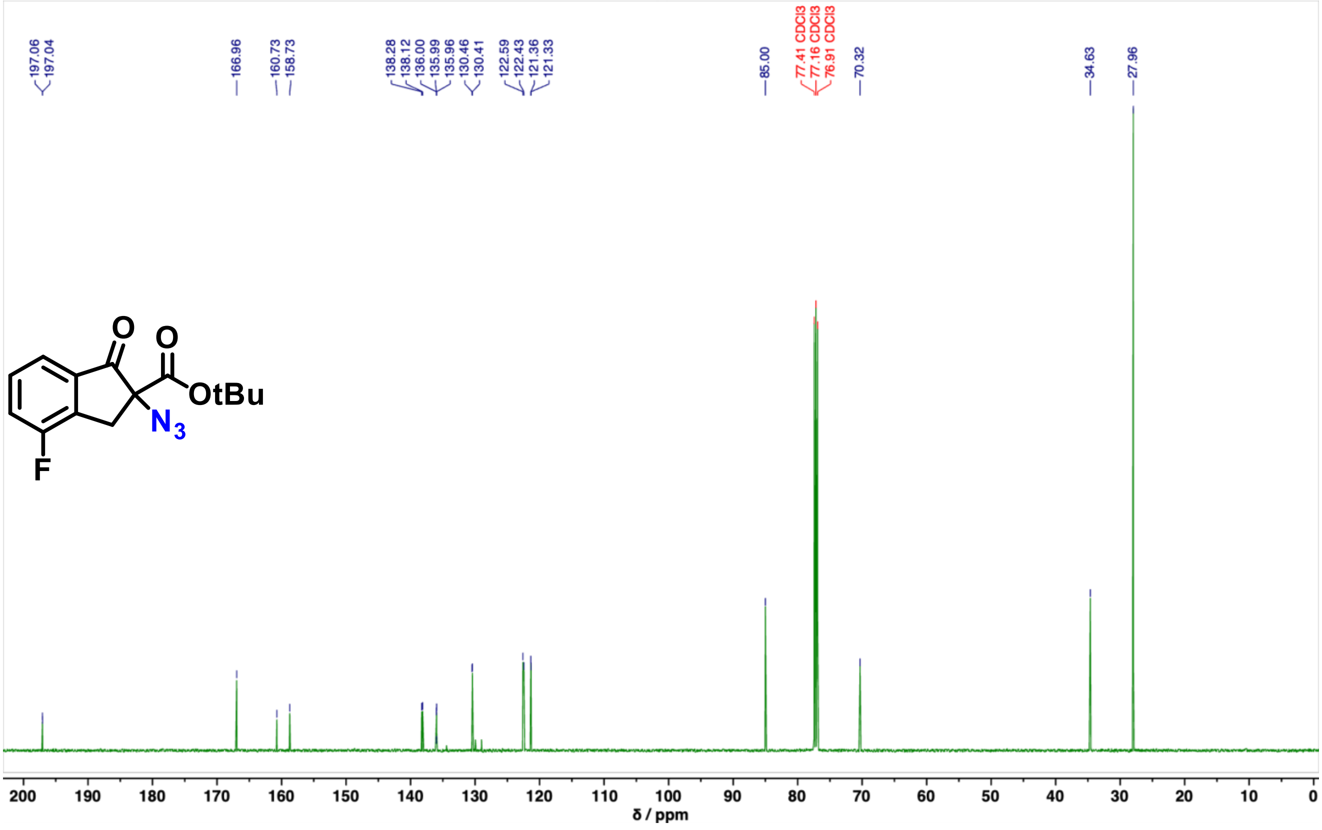

2g, ^{19}F-NMR (471 MHz, $CDCl_3$, 298 K, δ / ppm):

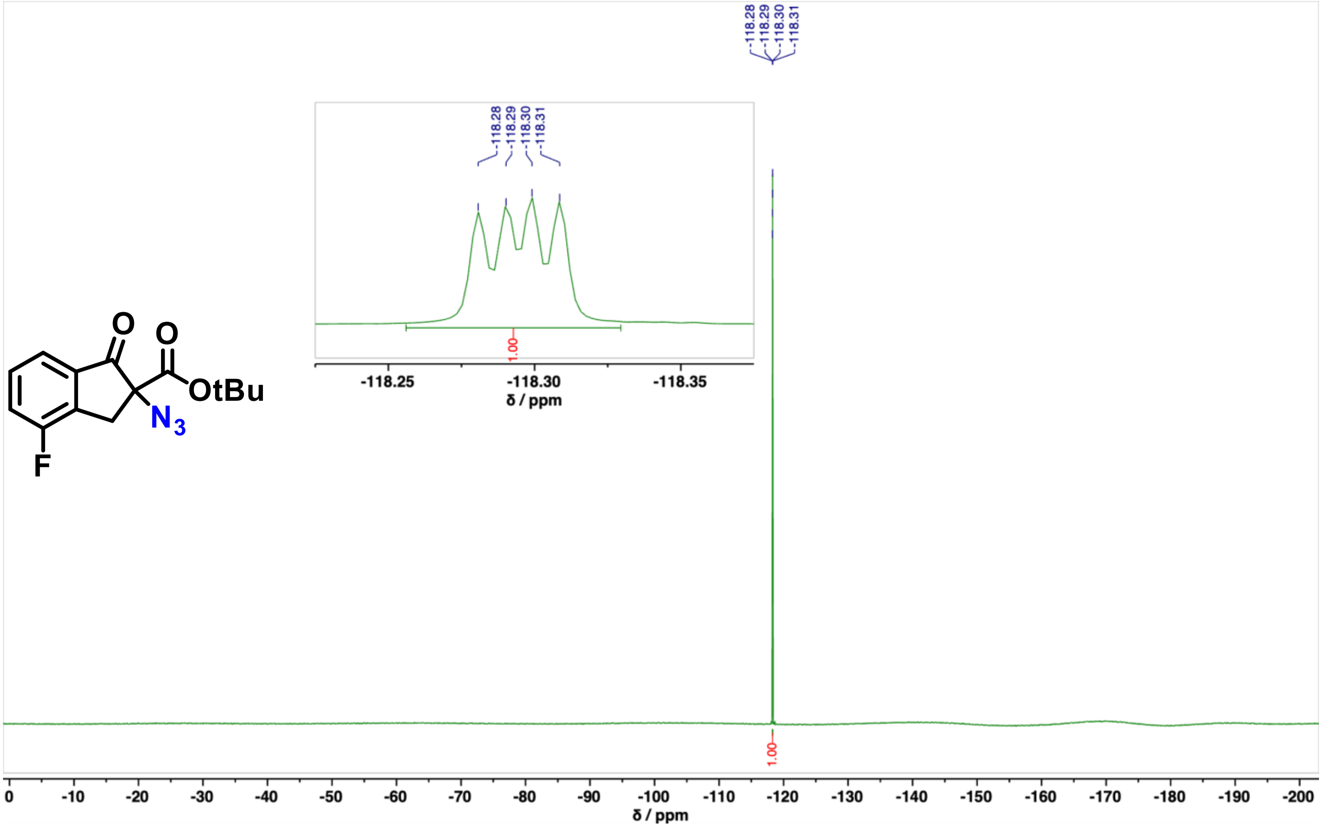

2h, ^{1}H-NMR (500 MHz, $CDCl_3$, 298 K, δ / ppm):

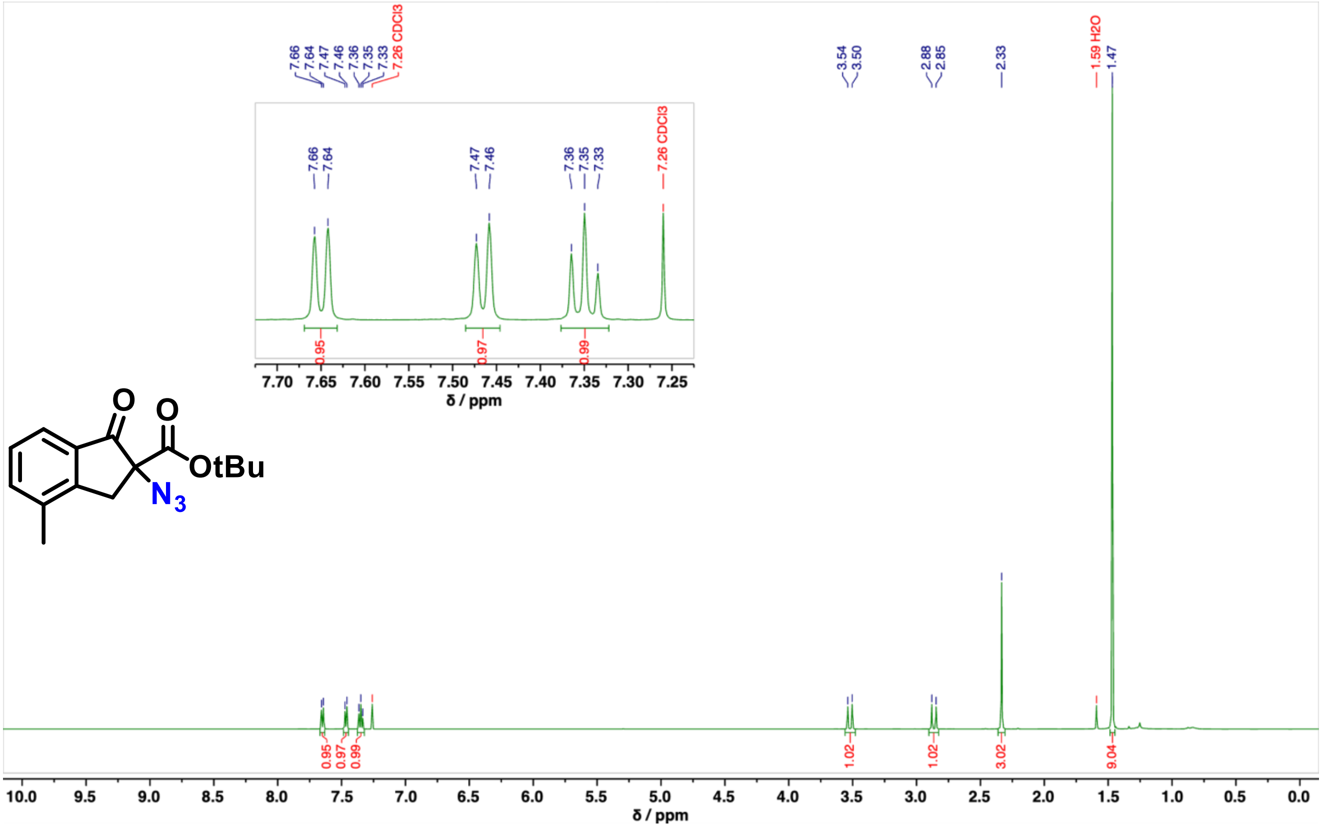

2h, ^{13}C-NMR (126 MHz, $CDCl_3$, 298 K, δ / ppm):

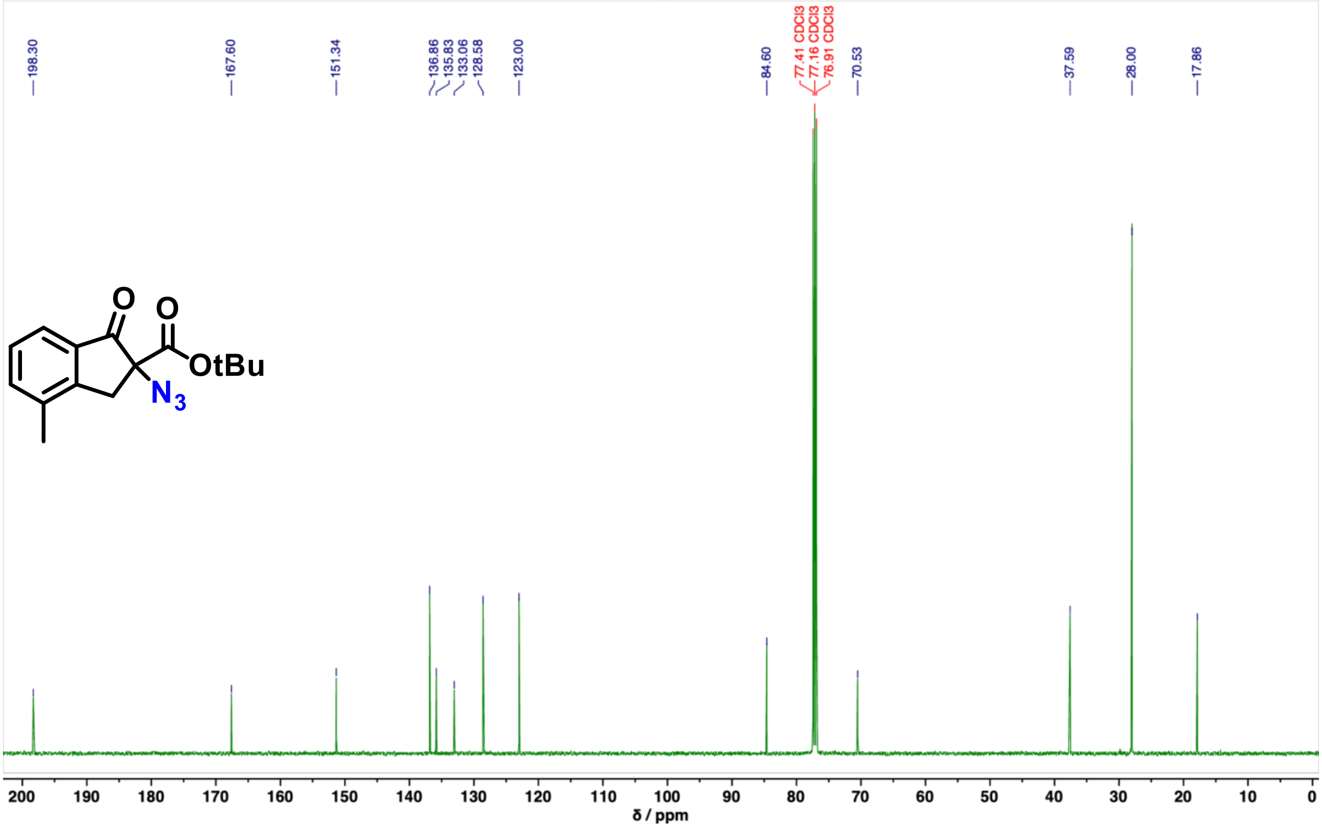

2i, ^{1}H-NMR (500 MHz, $CDCl_3$, 298 K, δ / ppm):

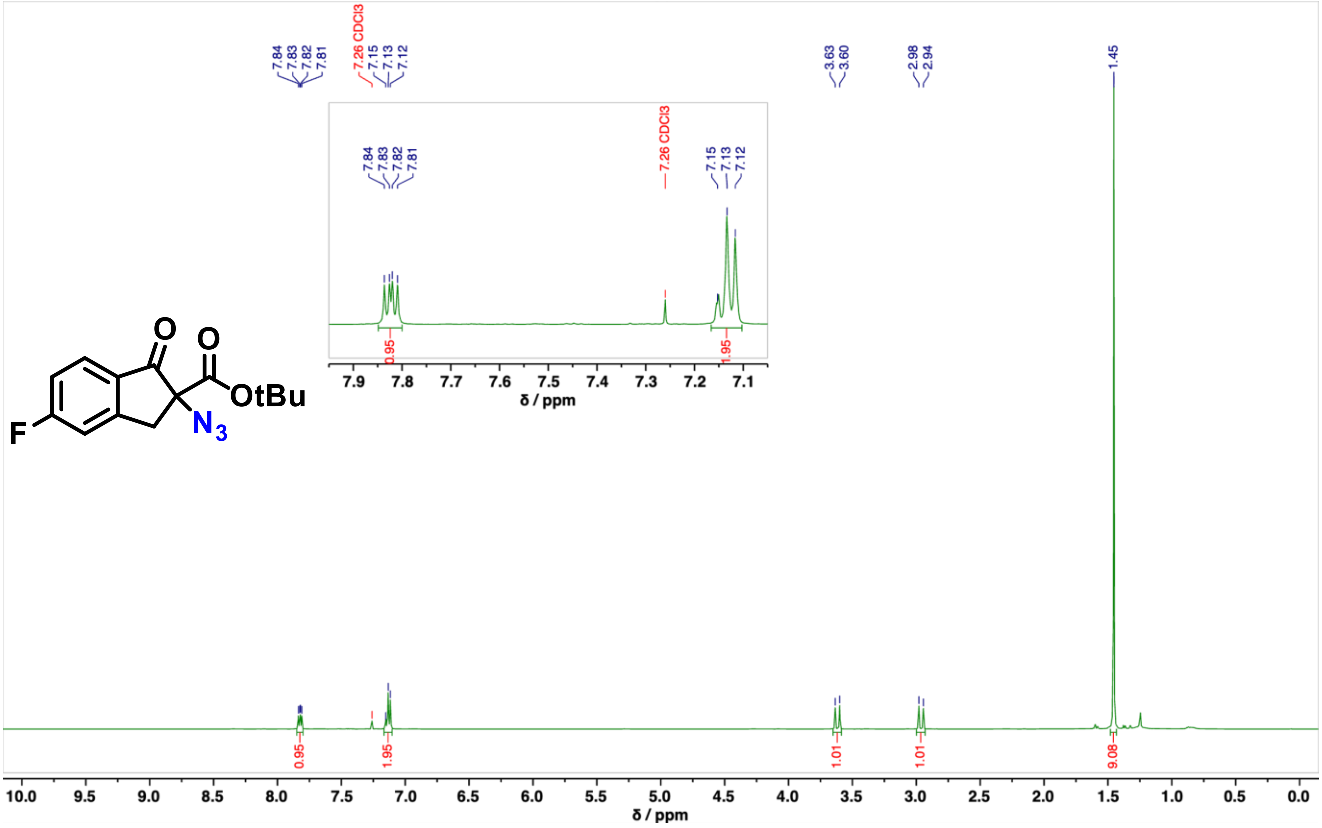

2i, ^{13}C-NMR (126 MHz, $CDCl_3$, 298 K, δ / ppm):

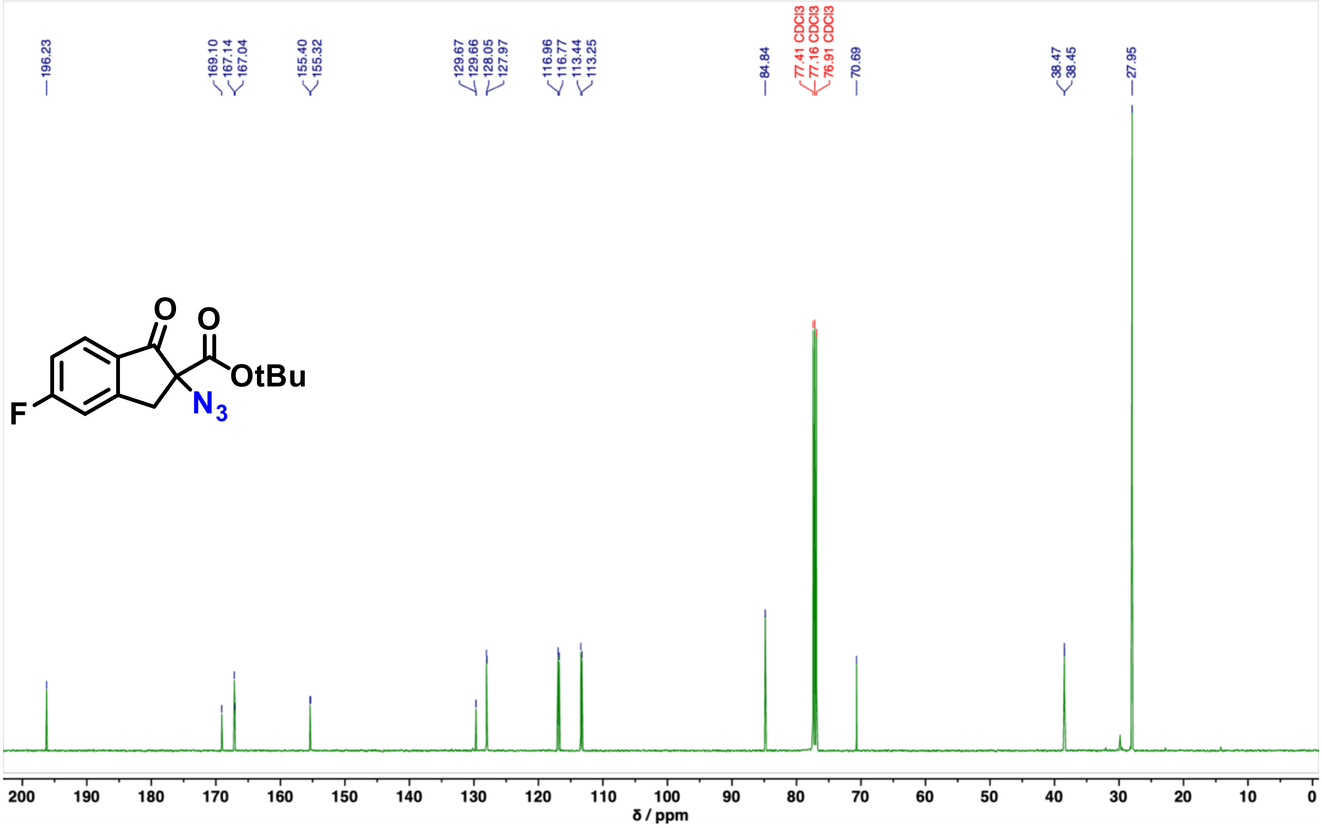

2i, ^{19}F-NMR (471 MHz, $CDCl_3$, 298 K, δ / ppm):

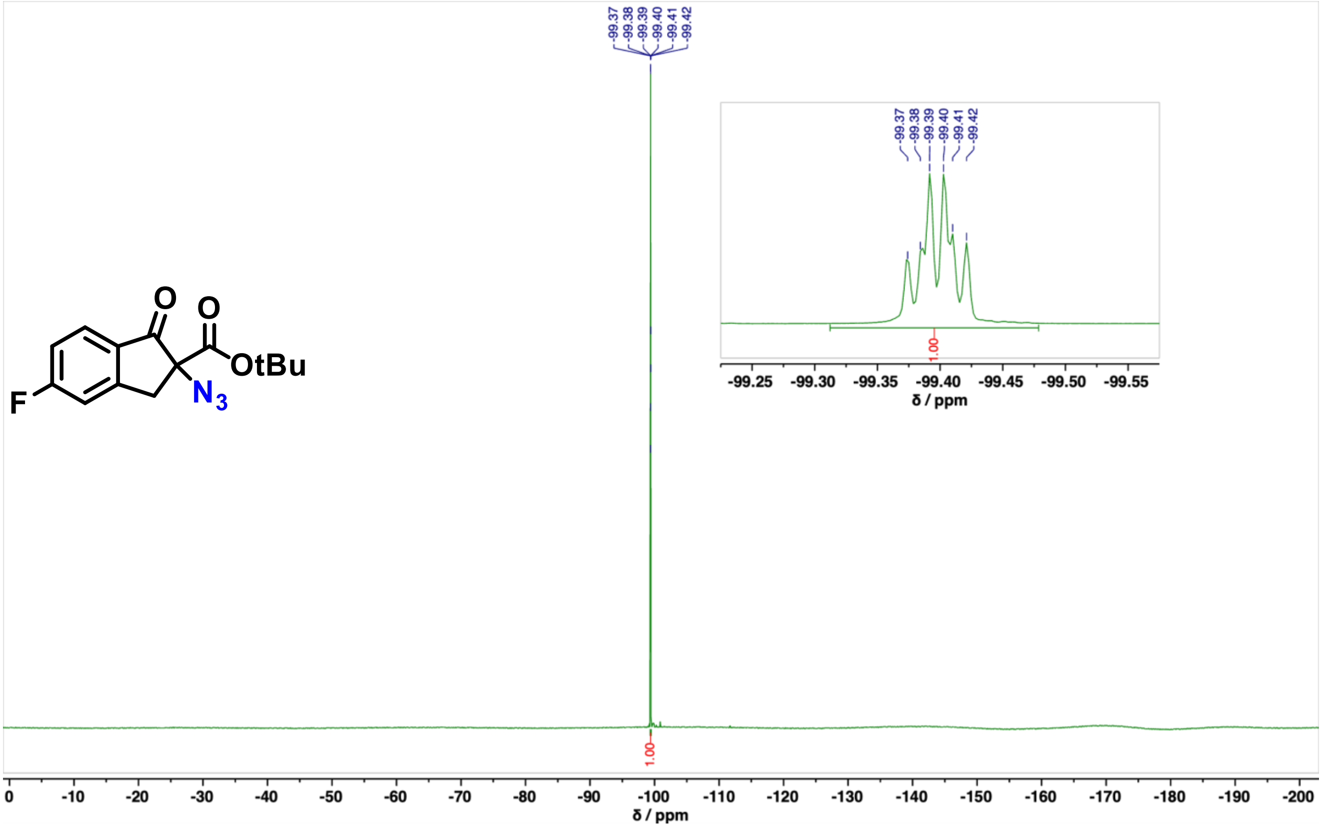

2j, ^{1}H-NMR (500 MHz, $CDCl_3$, 298 K, δ / ppm):

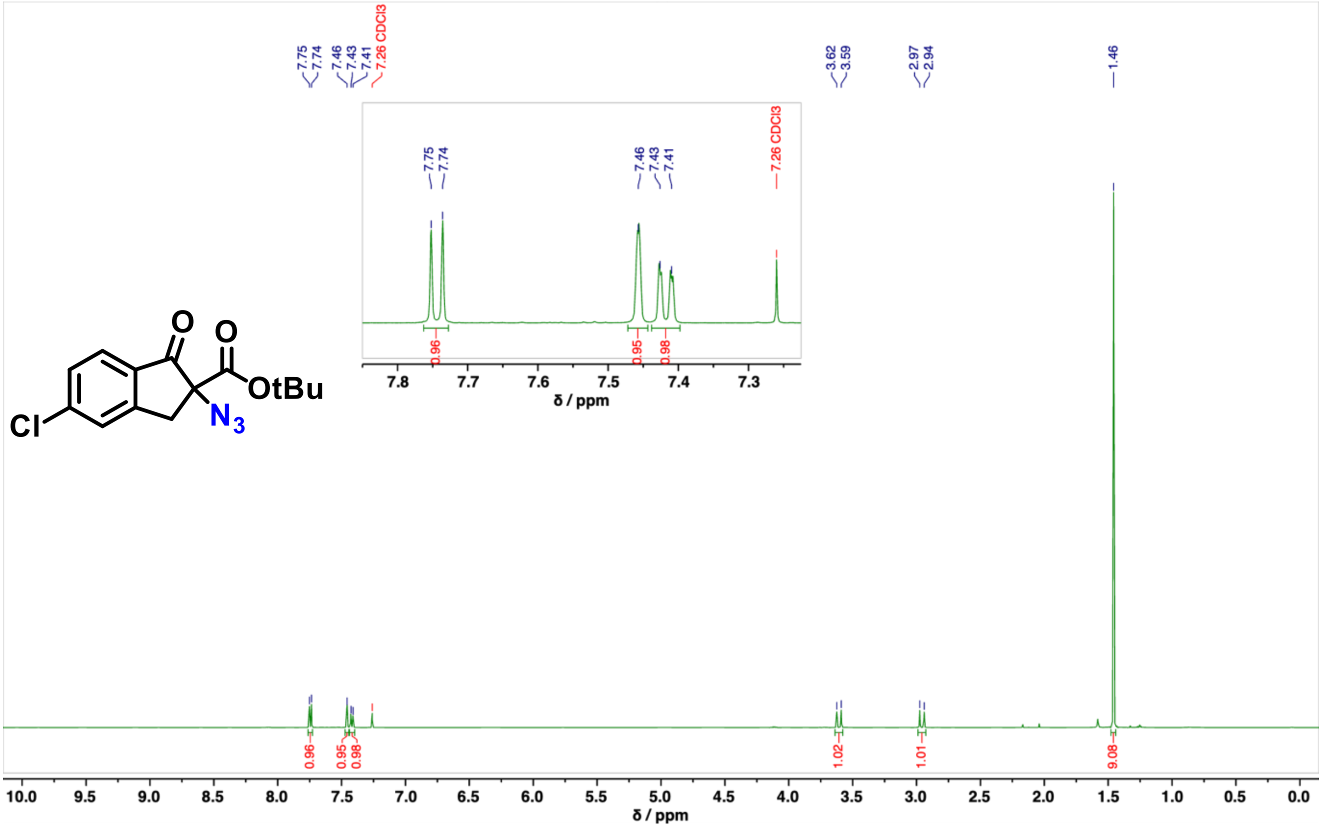

2j, ^{13}C-NMR (126 MHz, $CDCl_3$, 298 K, δ / ppm):

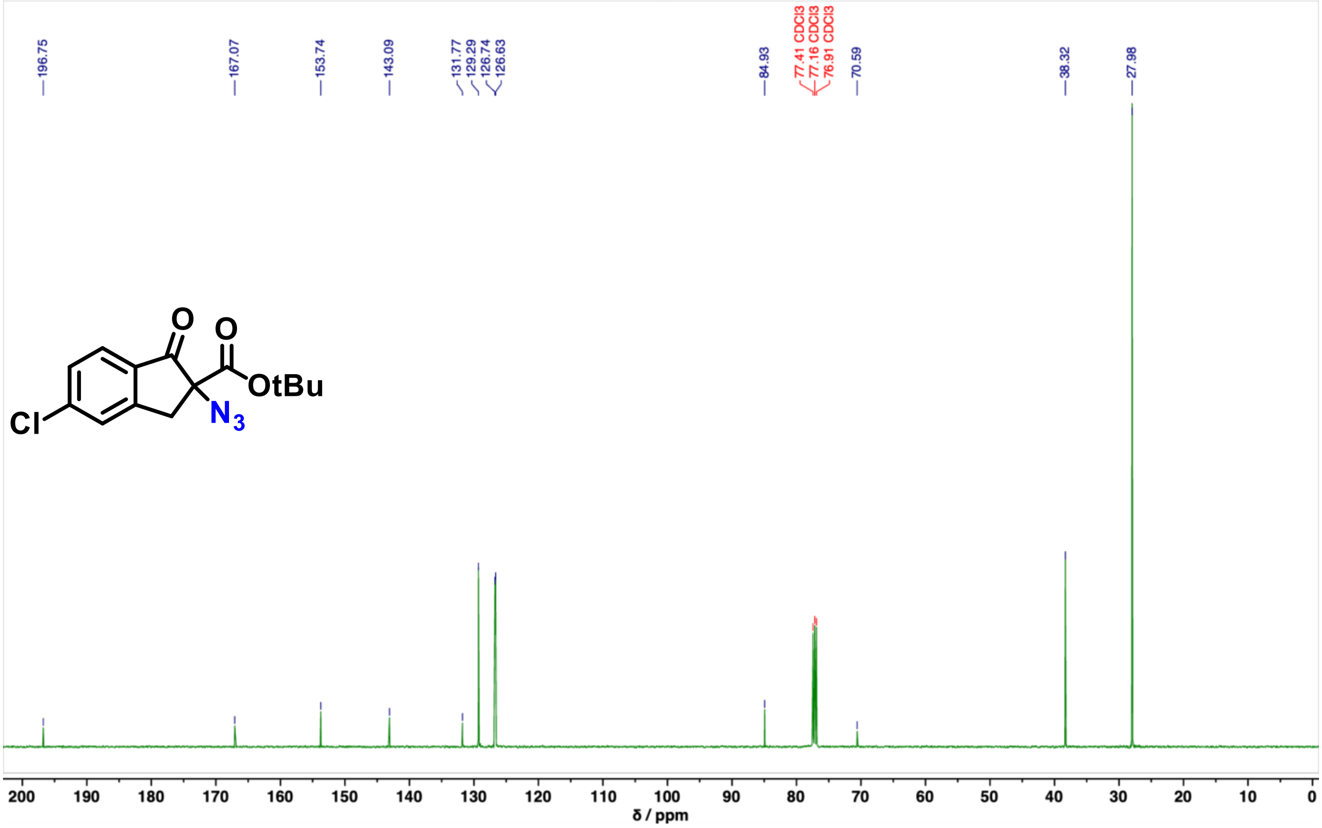

2k, ^{1}H-NMR (500 MHz, $CDCl_3$, 298 K, δ / ppm):

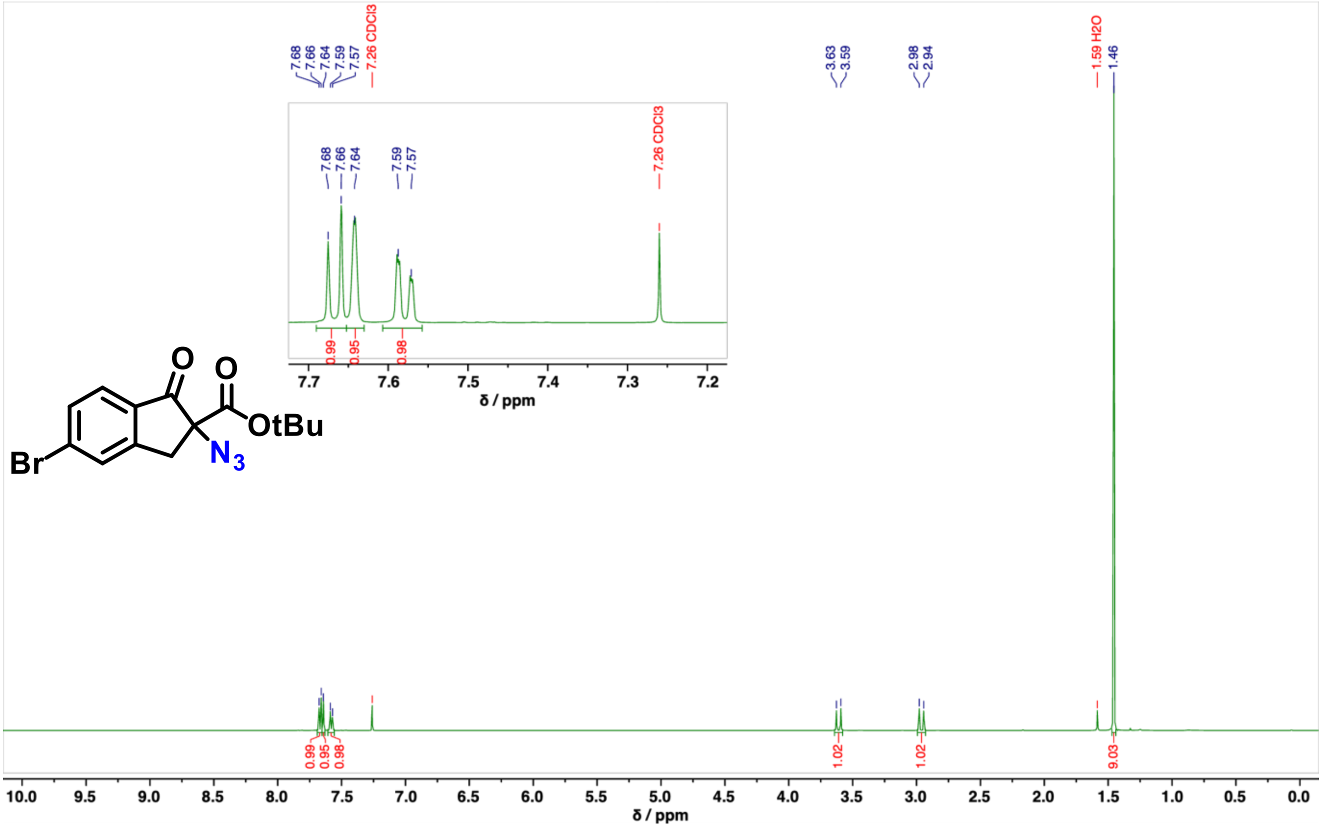

2k, ^{13}C-NMR (126 MHz, $CDCl_3$, 298 K, δ / ppm):

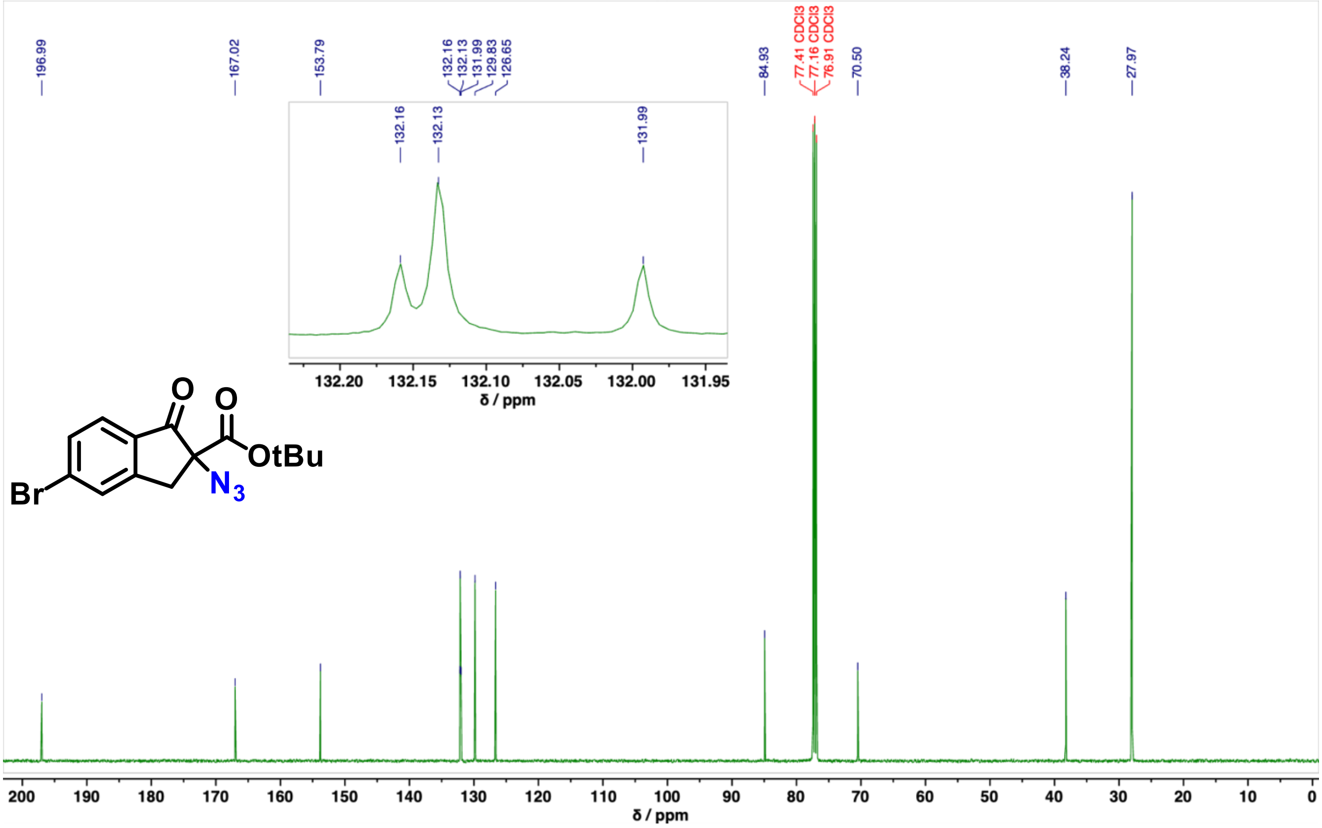

2l, ^{1}H-NMR (500 MHz, $CDCl_3$, 298 K, δ / ppm):

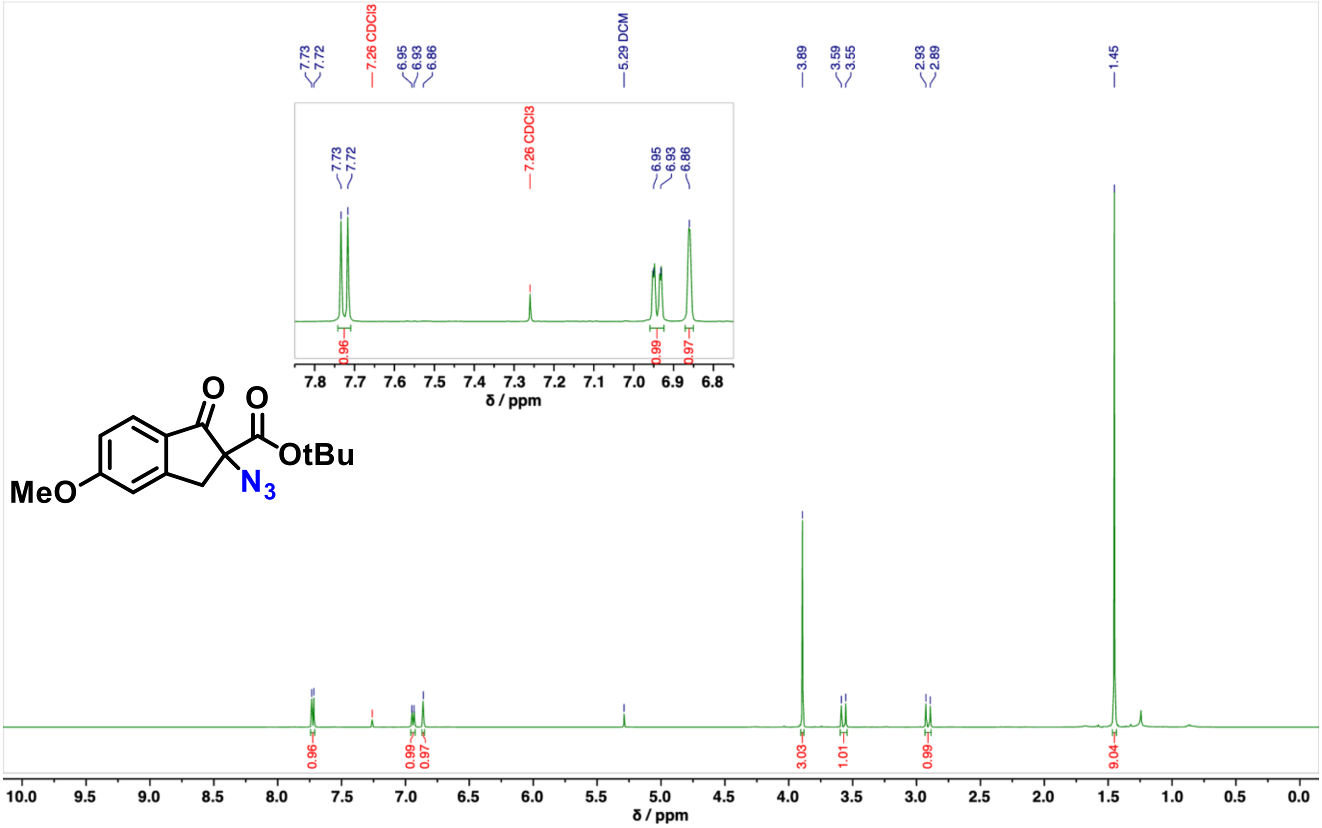

2l, ^{13}C-NMR (126 MHz, $CDCl_3$, 298 K, δ / ppm):

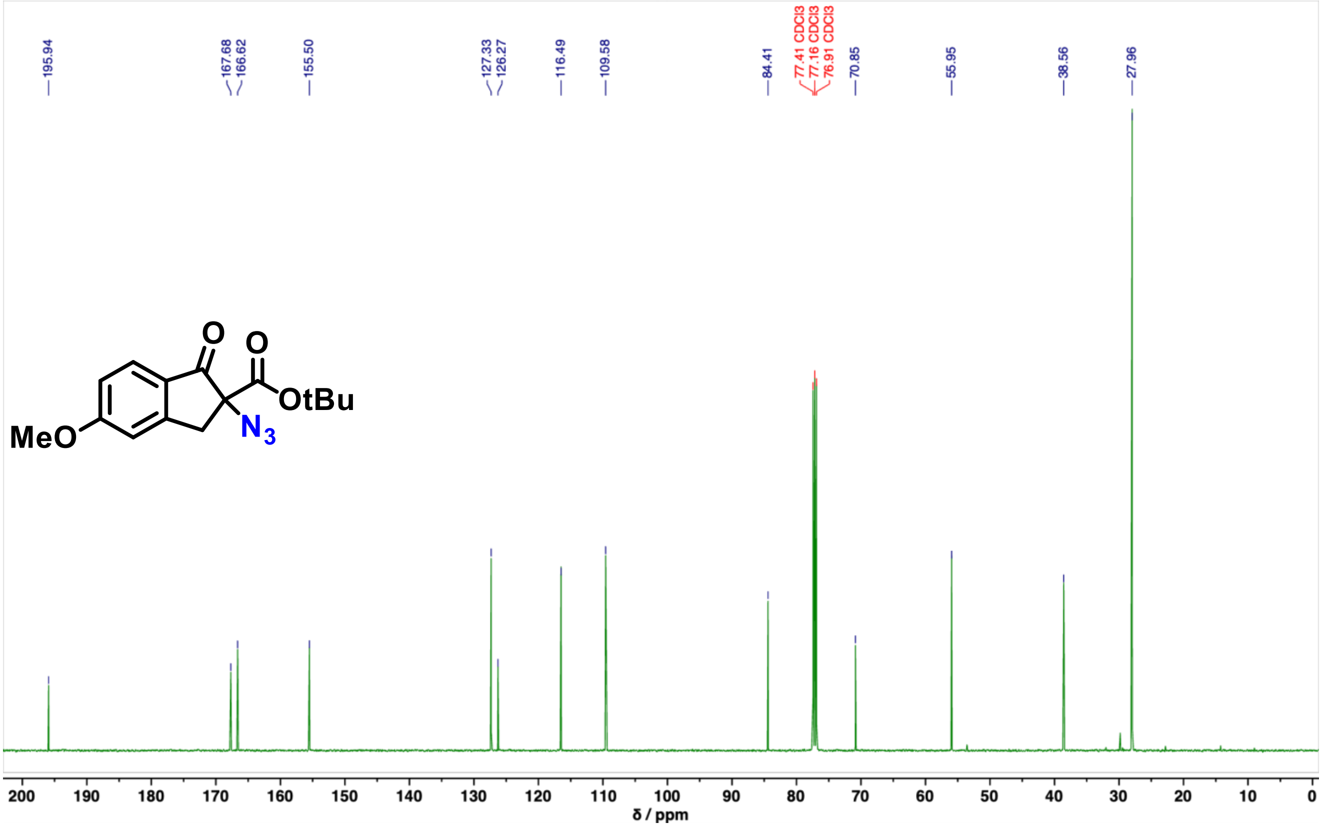

**2m, **

2m, ^{1}H-NMR (500 MHz, $CDCl_3$, 298 K, δ / ppm):

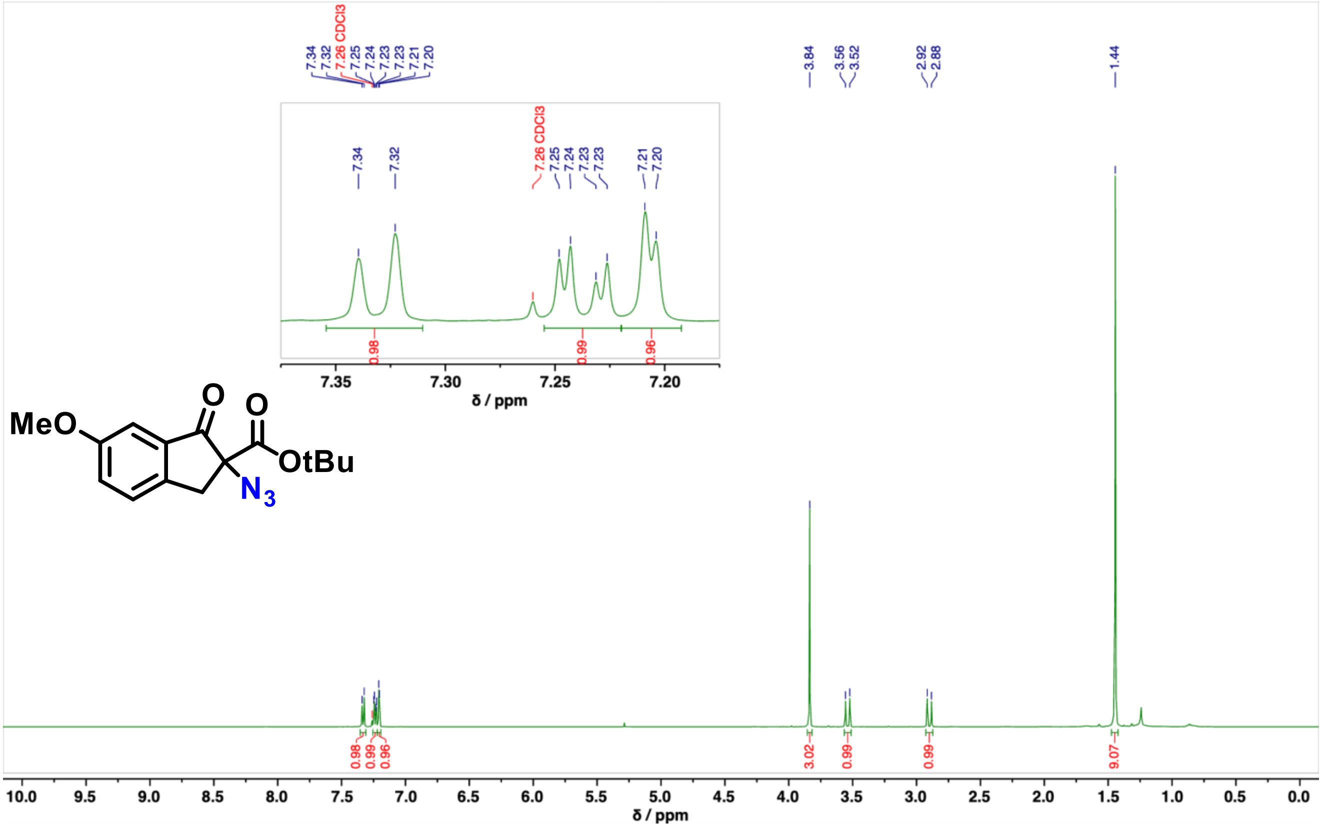

2m, ^{13}C-NMR (126 MHz, $CDCl_3$, 298 K, δ / ppm):

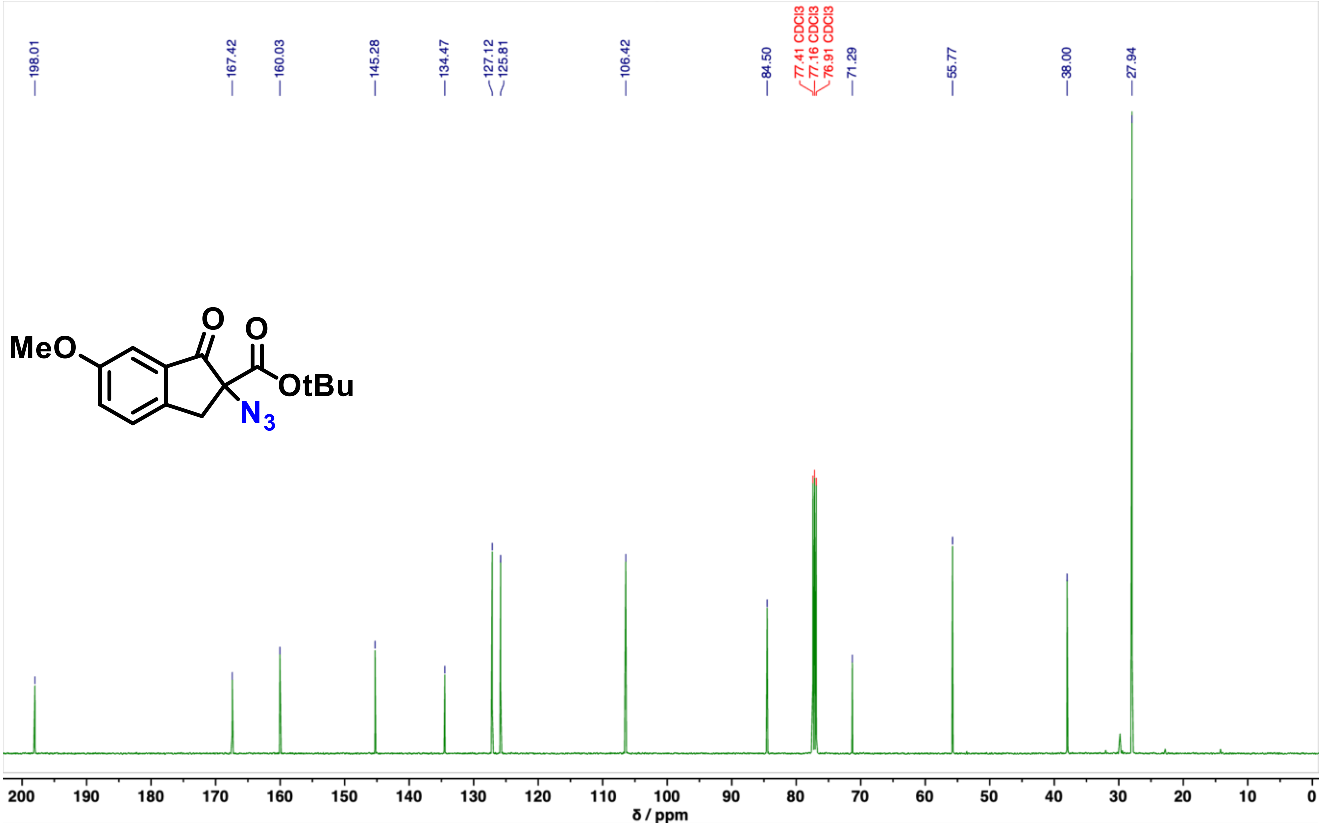

2n, ^{1}H-NMR (500 MHz, $CDCl_3$, 298 K, δ / ppm):

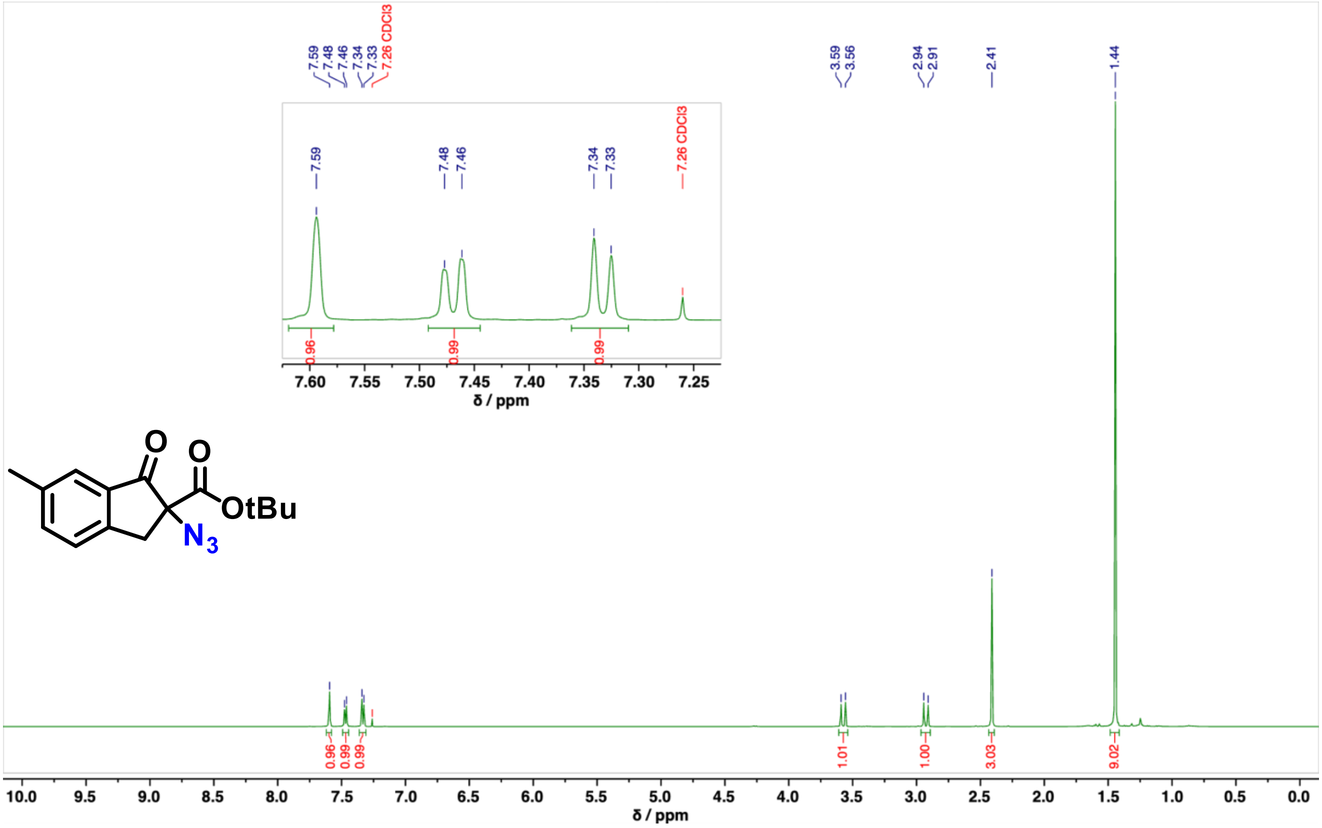

2n, ^{13}C-NMR (126 MHz, $CDCl_3$, 298 K, δ / ppm):

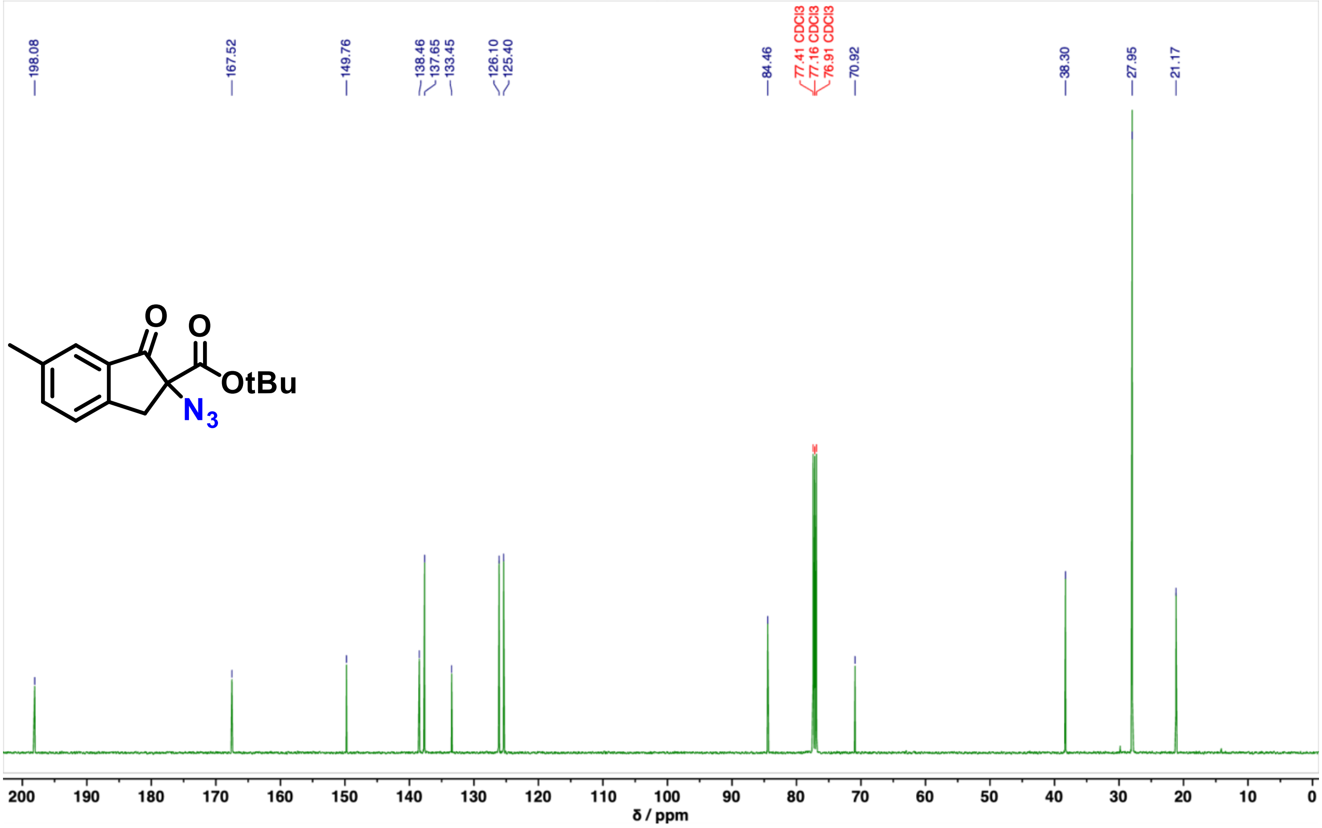

5a, ^{1}H-NMR (700 MHz, CDCl$_3$, 298 K, δ / ppm):

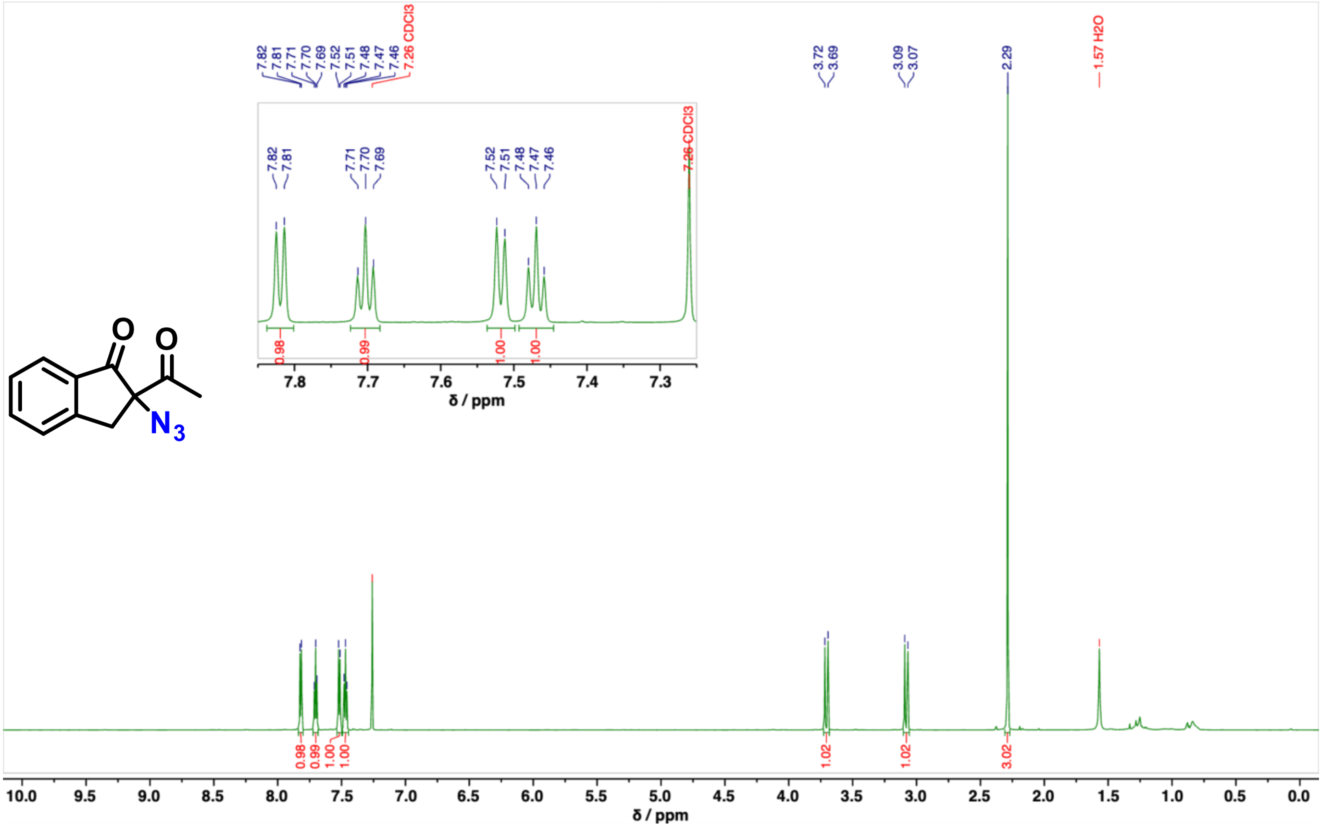

5a, ^{13}C-NMR (176 MHz, CDCl$_3$, 298 K, δ / ppm):

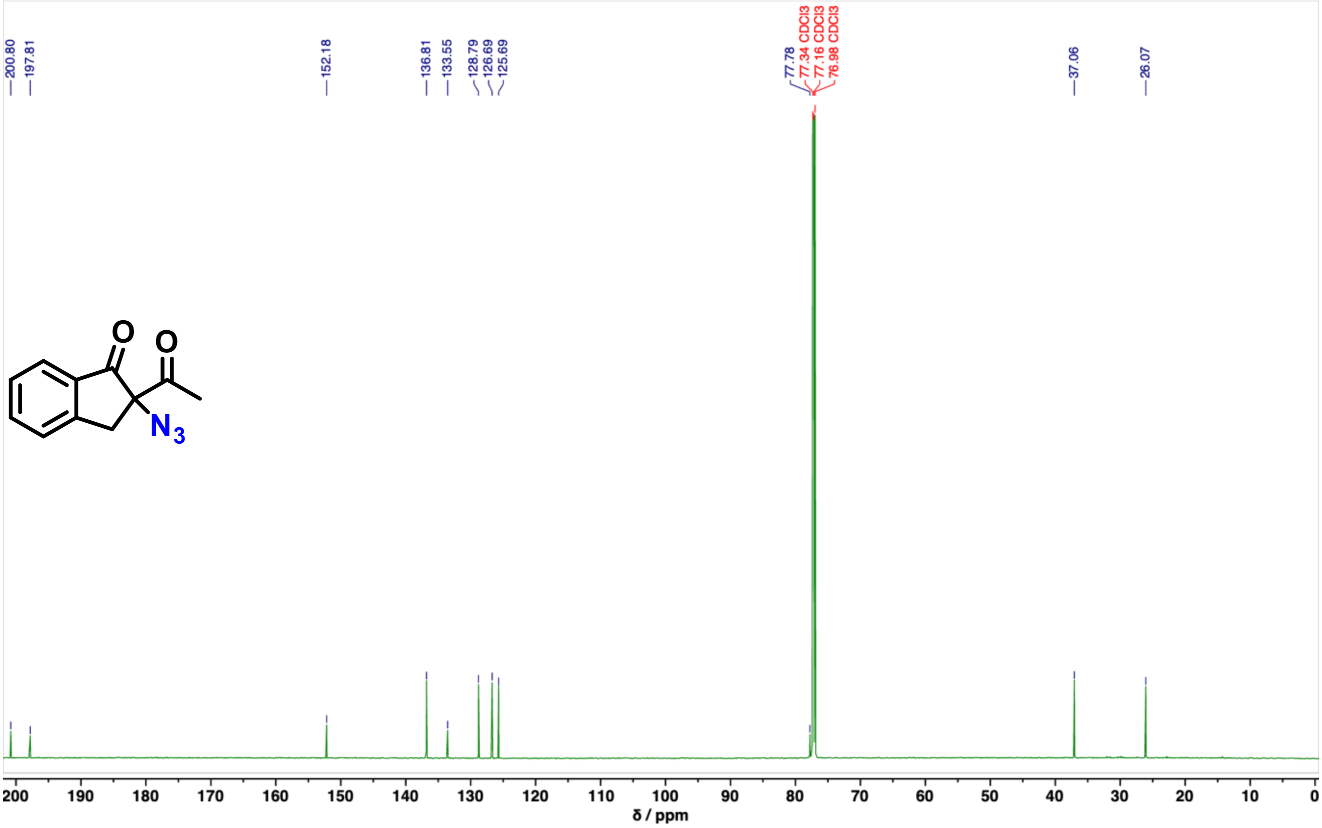

5b, ^{1}H-NMR (700 MHz, $CDCl_3$, 298 K, δ / ppm):

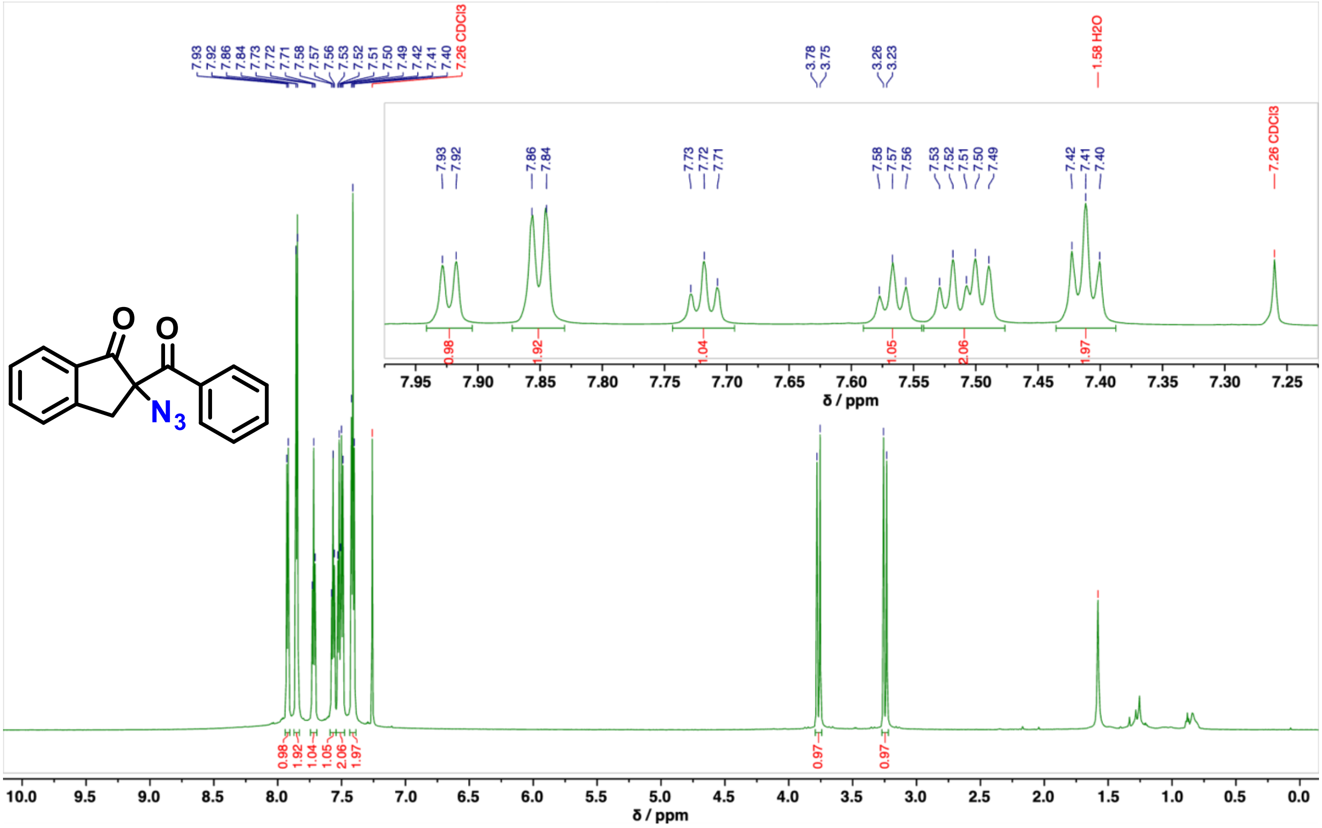

5b, ^{13}C-NMR (176 MHz, $CDCl_3$, 298 K, δ / ppm):

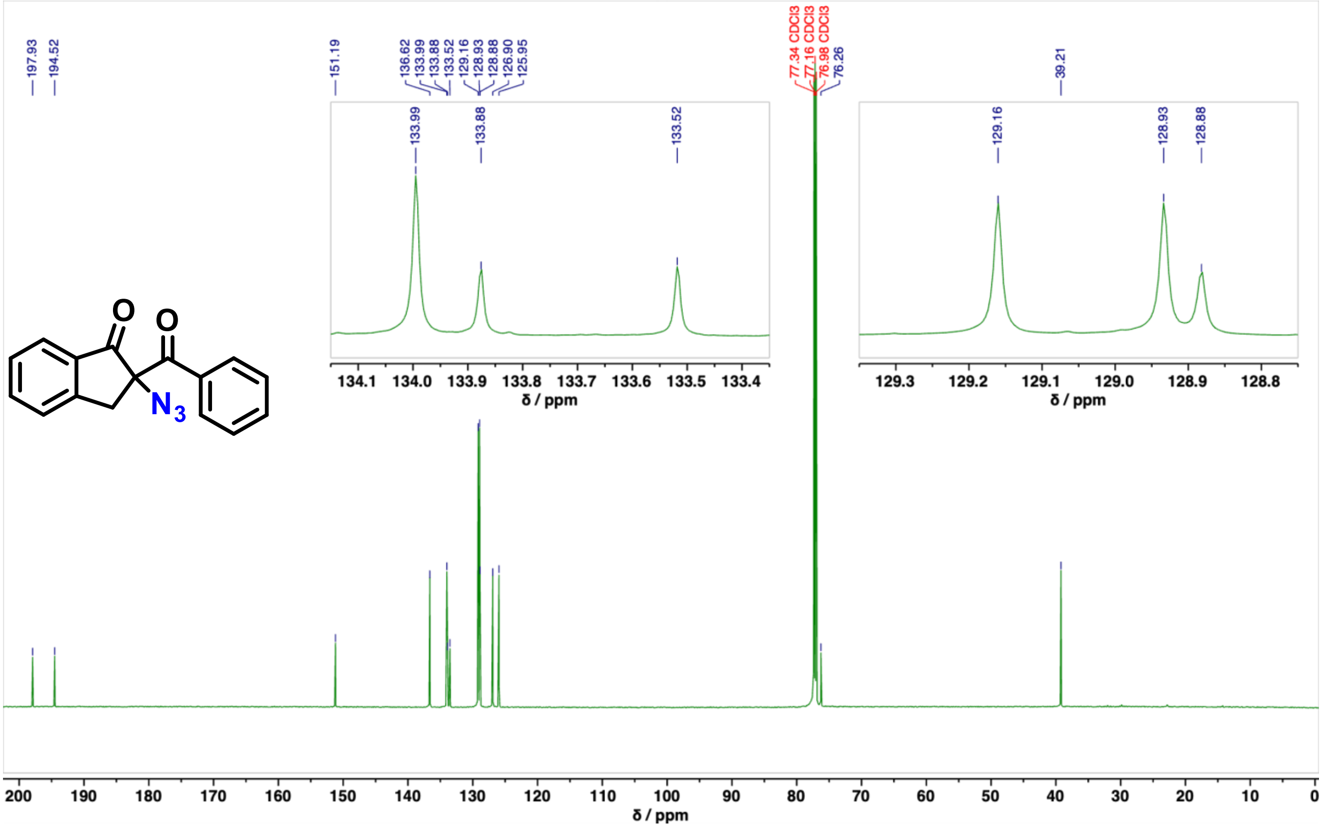

6, ^{1}H-NMR (700 MHz, $CDCl_3$, 298 K, δ / ppm):

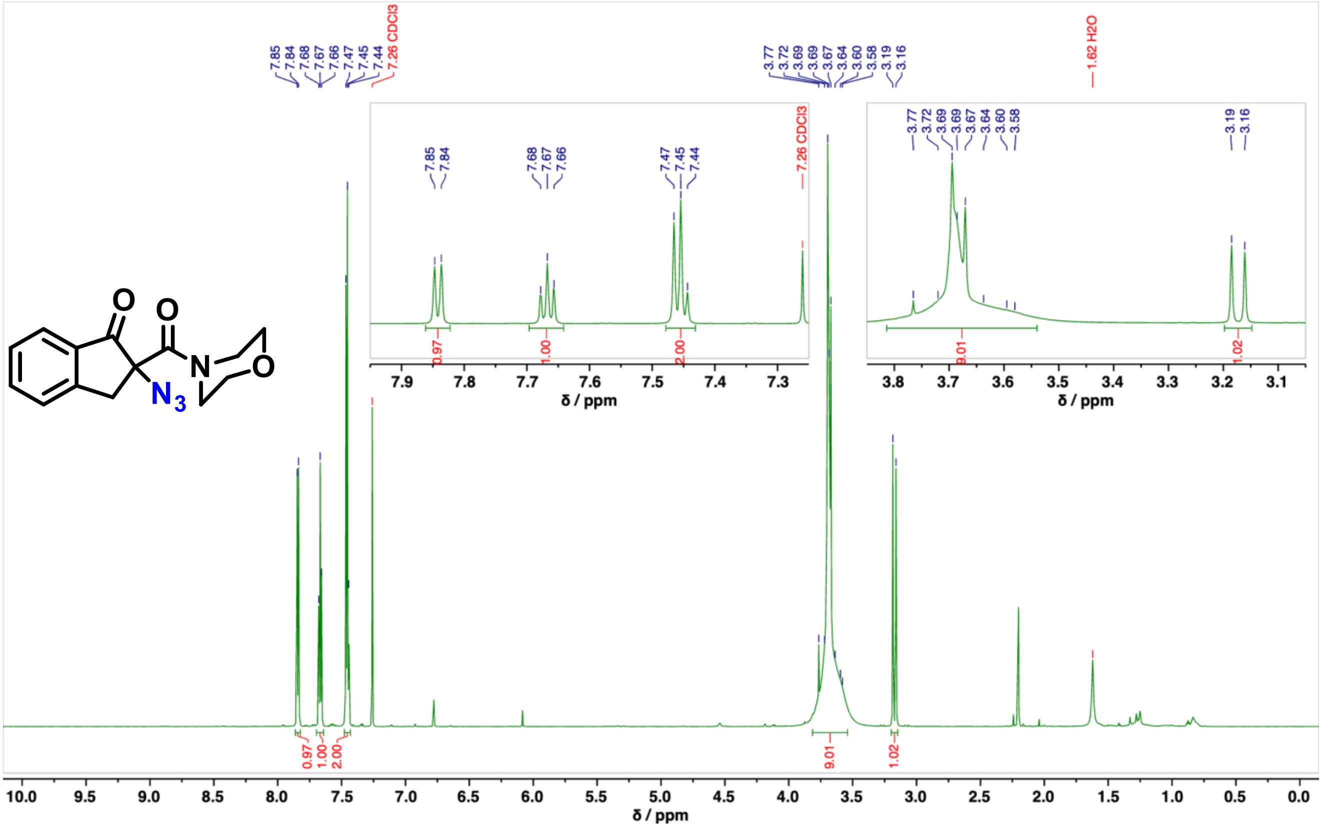

6, ^{13}C-NMR (176 MHz, $CDCl_3$, 298 K, δ / ppm):

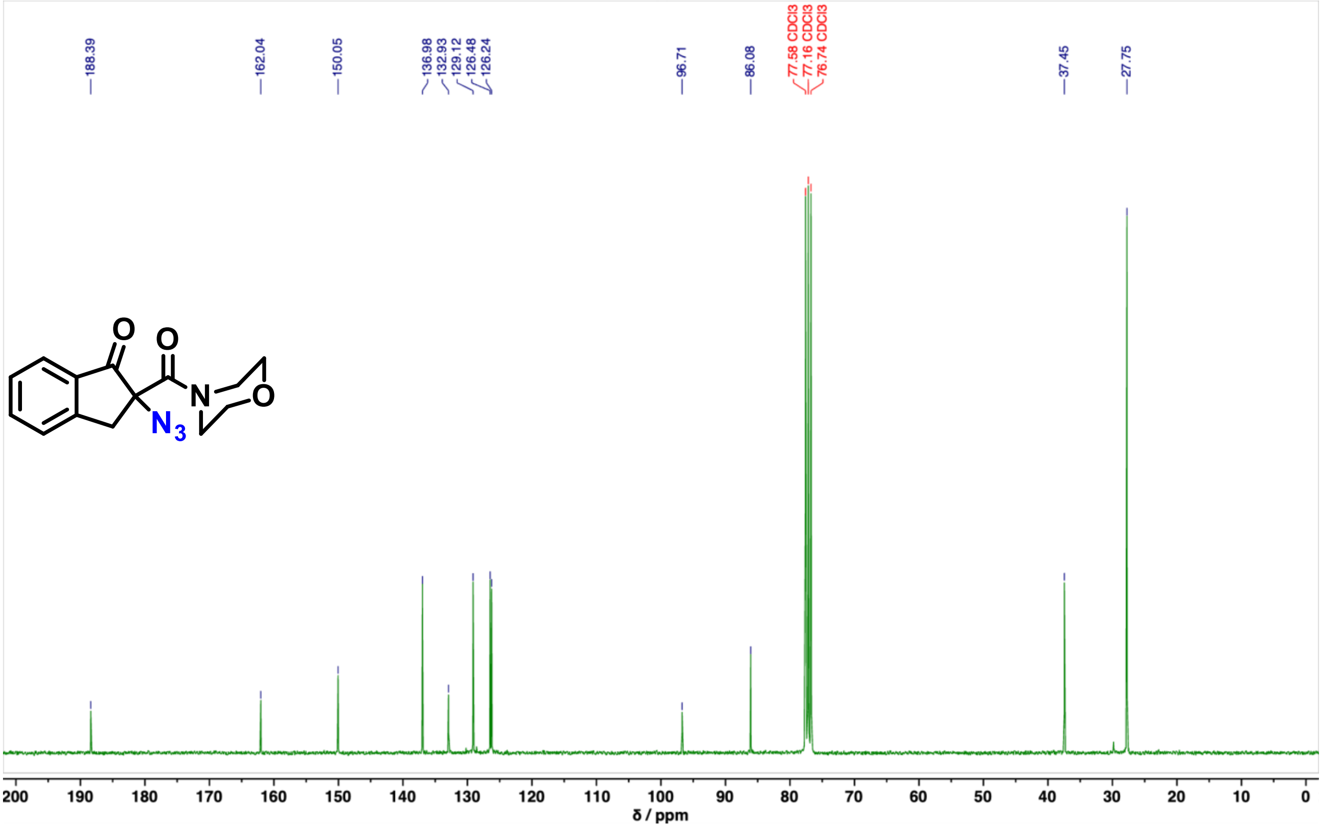

7a, ^{1}H-NMR (700 MHz, CDCl3, 298 K, δ / ppm):

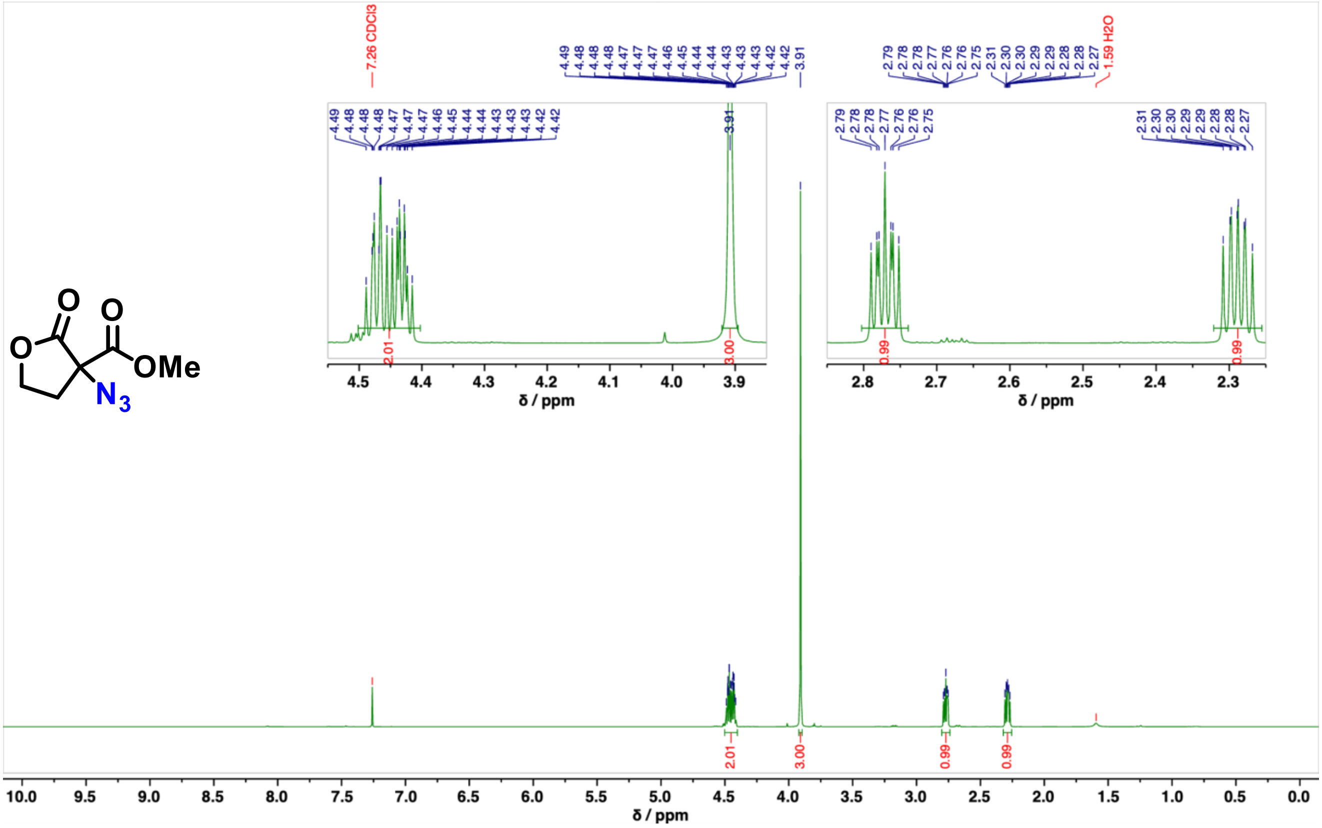

7a, ^{13}C-NMR (176 MHz, CDCl3, 298 K, δ / ppm):

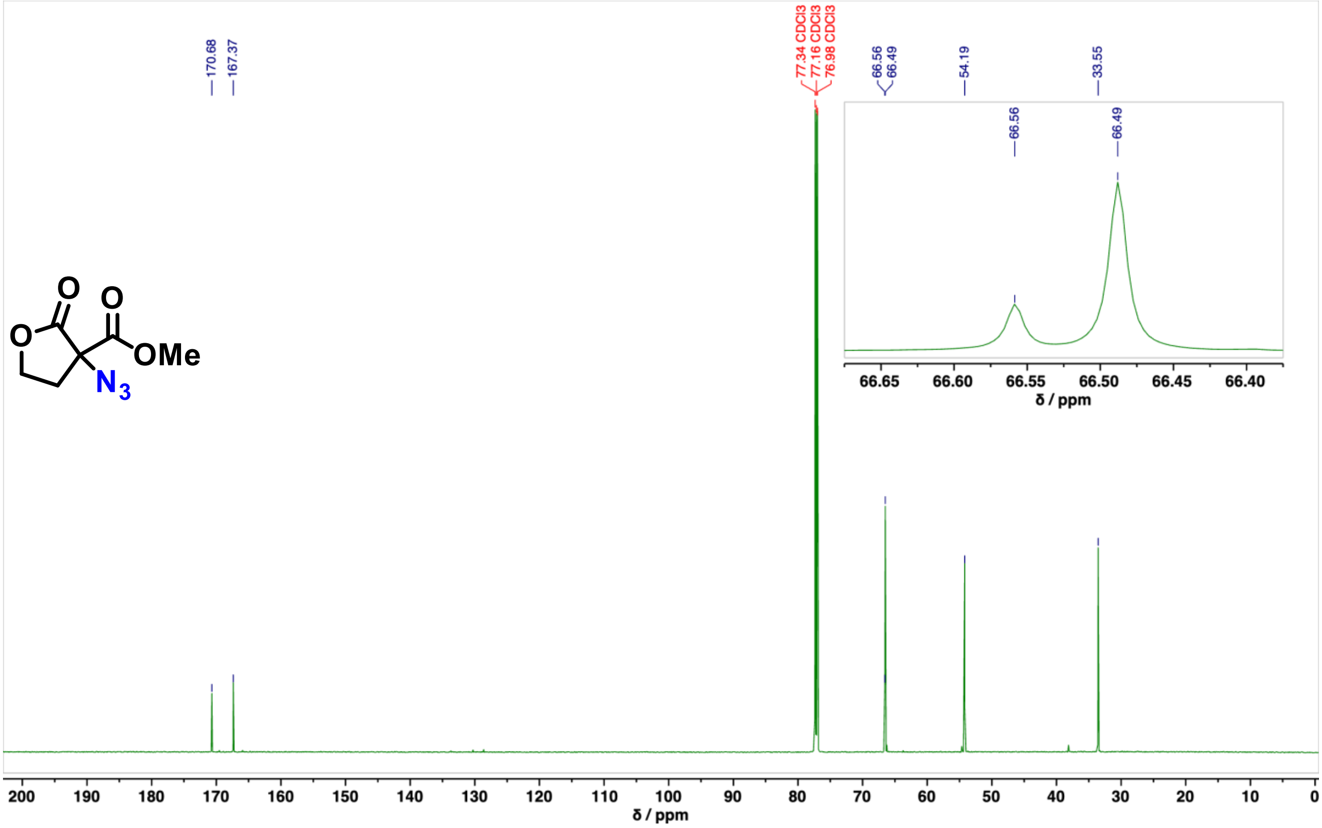

7b, ^{1}H-NMR (700 MHz, $CDCl_3$, 298 K, δ / ppm):

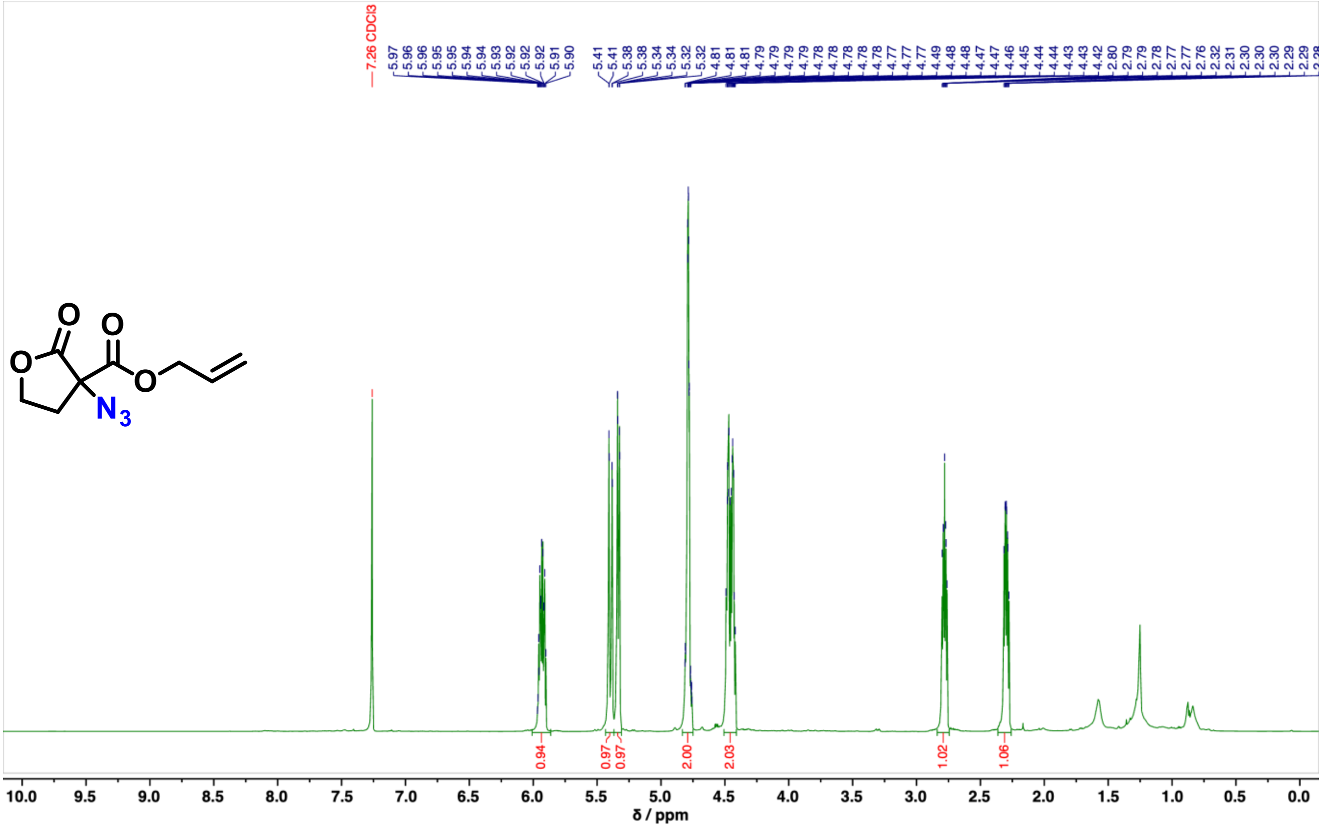

7b, ^{13}C-NMR (176 MHz, $CDCl_3$, 298 K, δ / ppm):

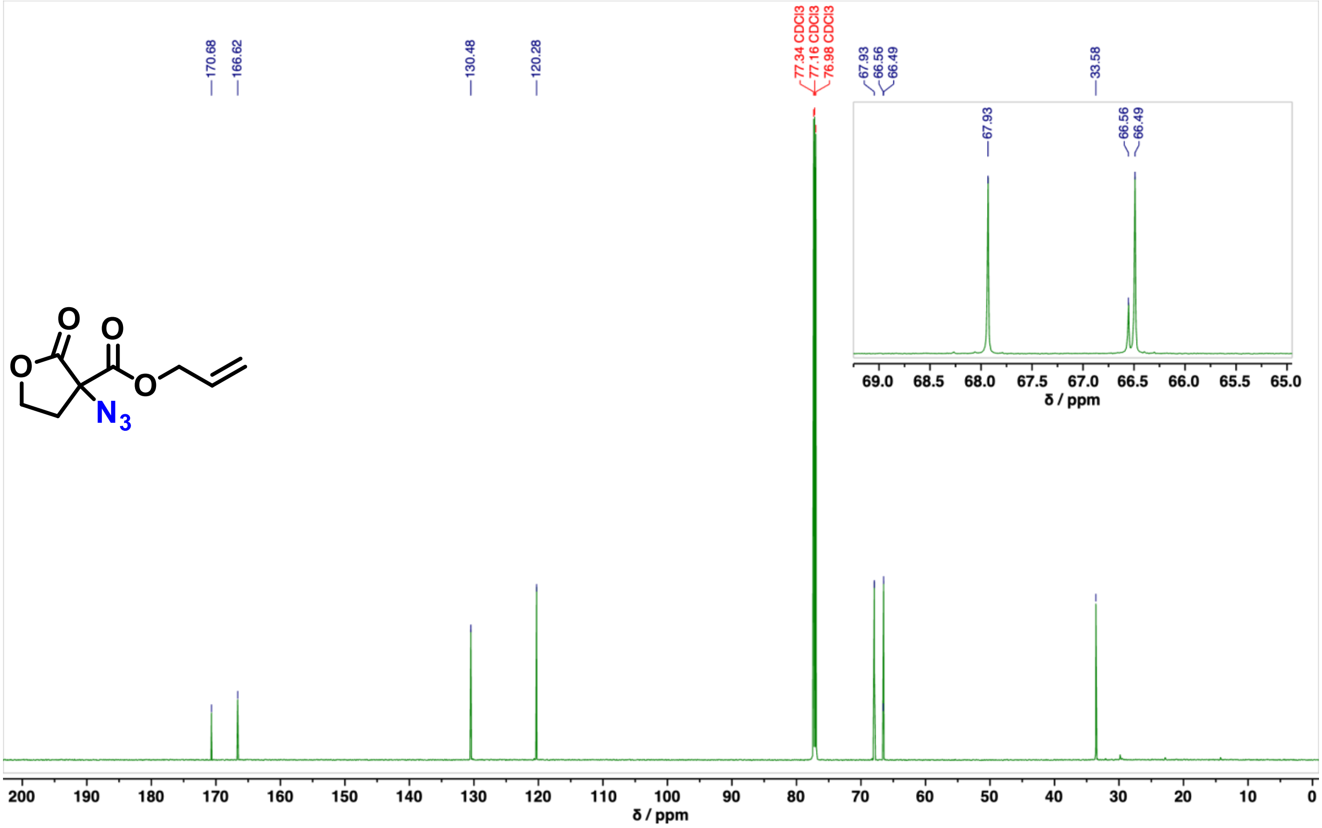

7c, ^{1}H-NMR (700 MHz, $CDCl_3$, 298 K, *δ* / ppm):

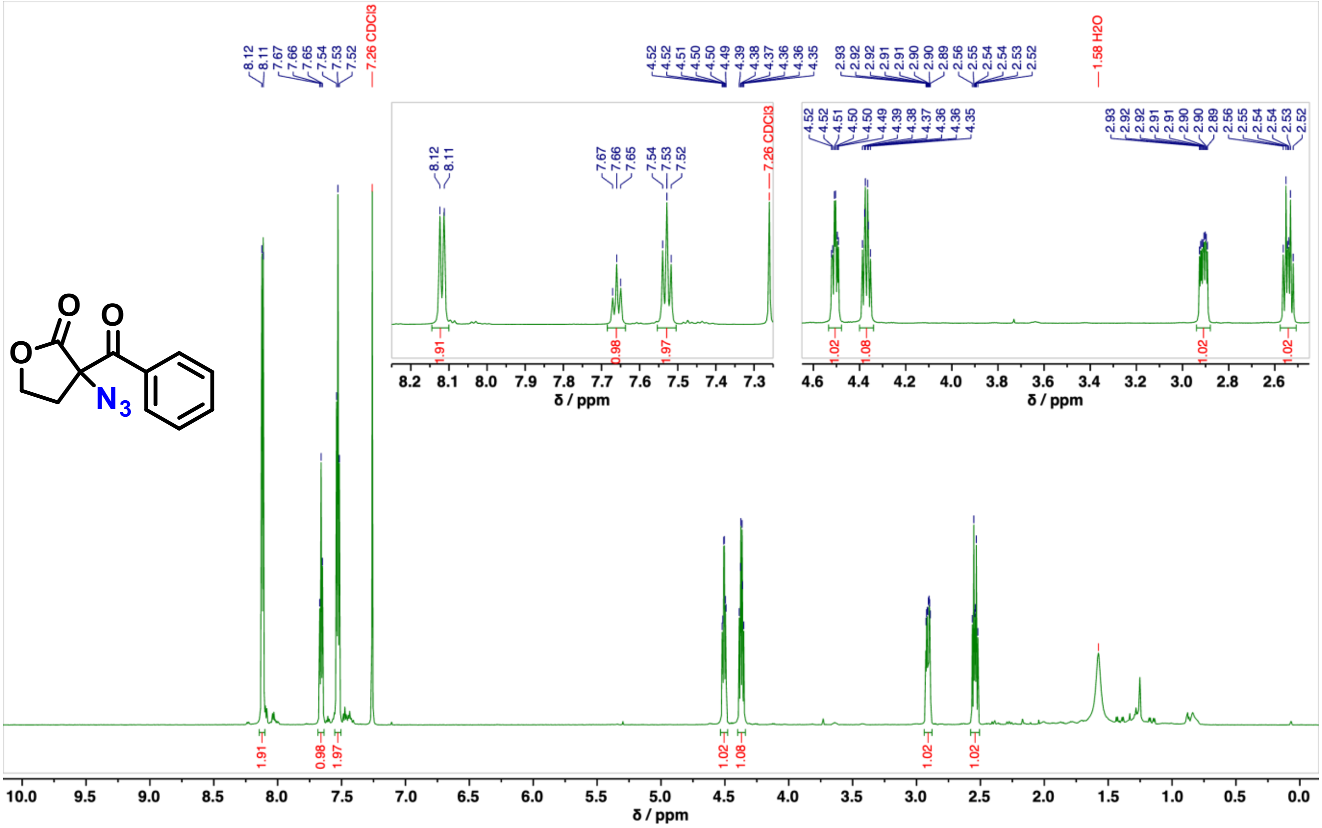

7c, ^{13}C-NMR (176 MHz, $CDCl_3$, 298 K, *δ* / ppm):

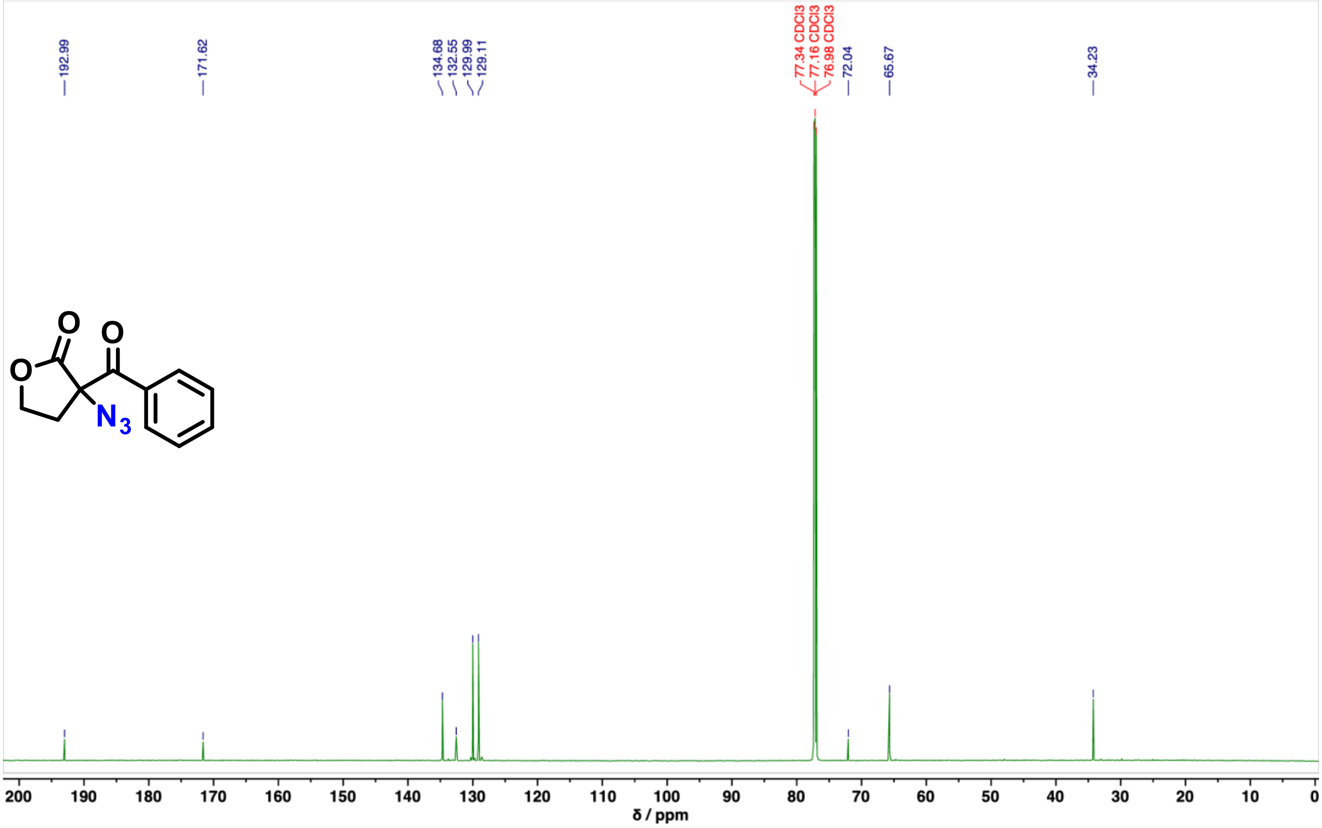

10a, ^{1}H-NMR (700 MHz, $CDCl_3$, 298 K, *δ* / ppm):

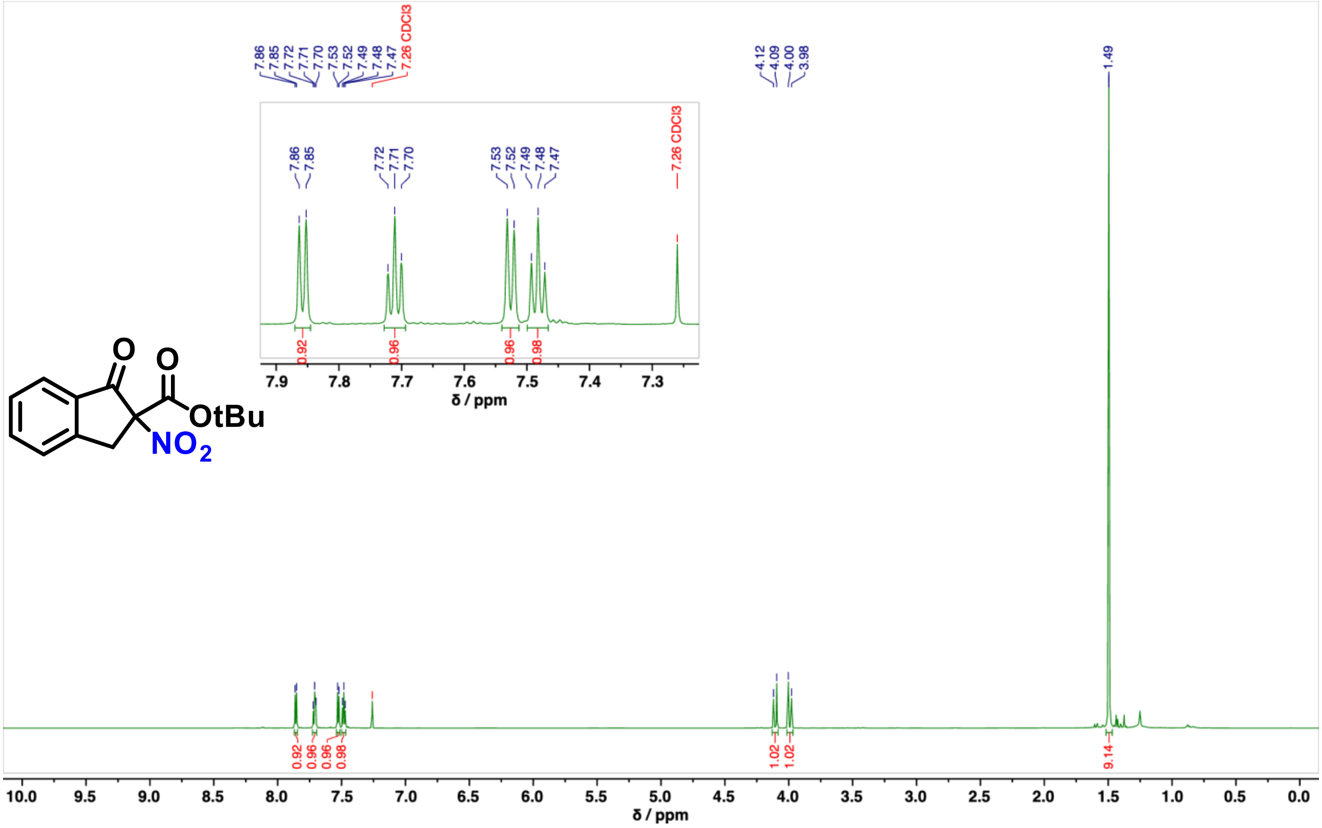

10a, ^{13}C-NMR (176 MHz, $CDCl_3$, 298 K, *δ* / ppm):

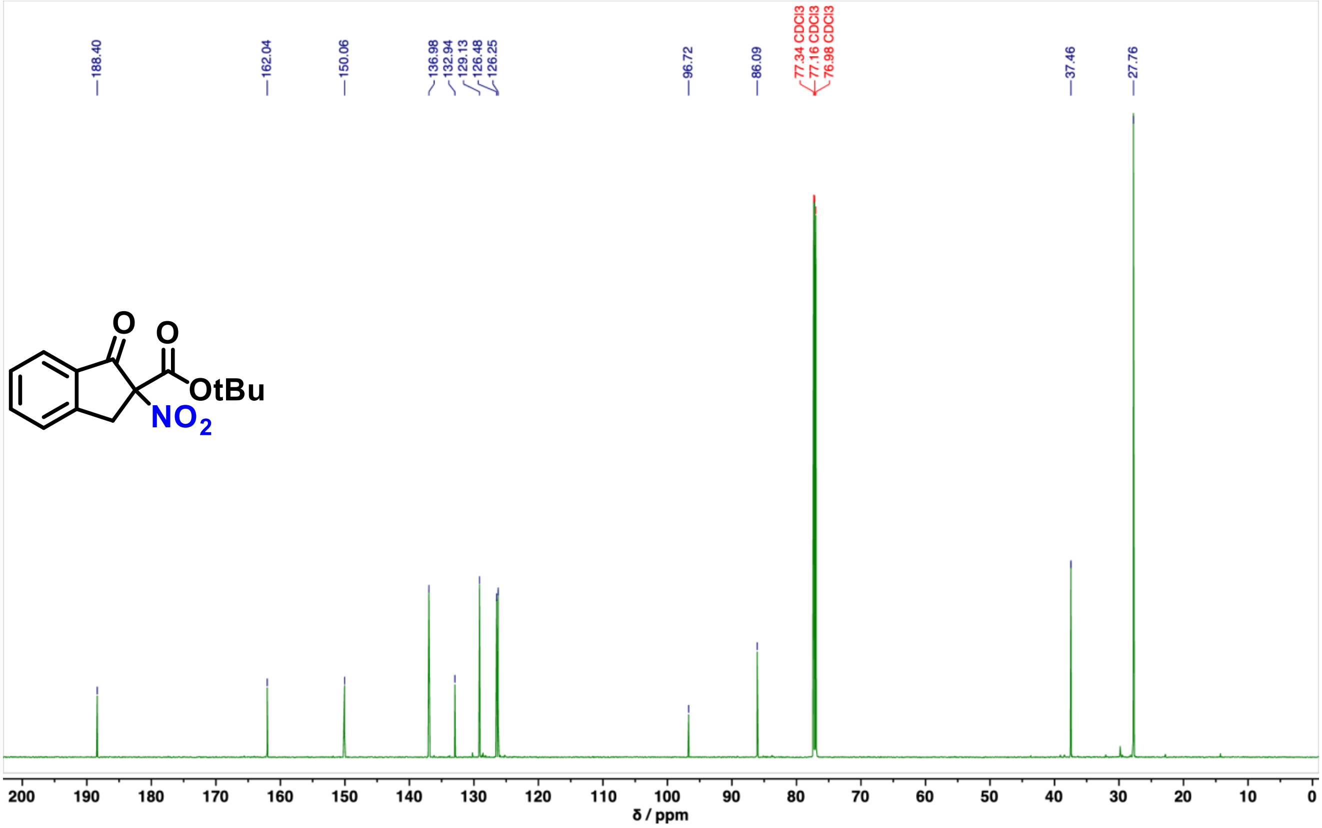

10b, ^{1}H-NMR (700 MHz, $CDCl_3$, 298 K, δ / ppm):

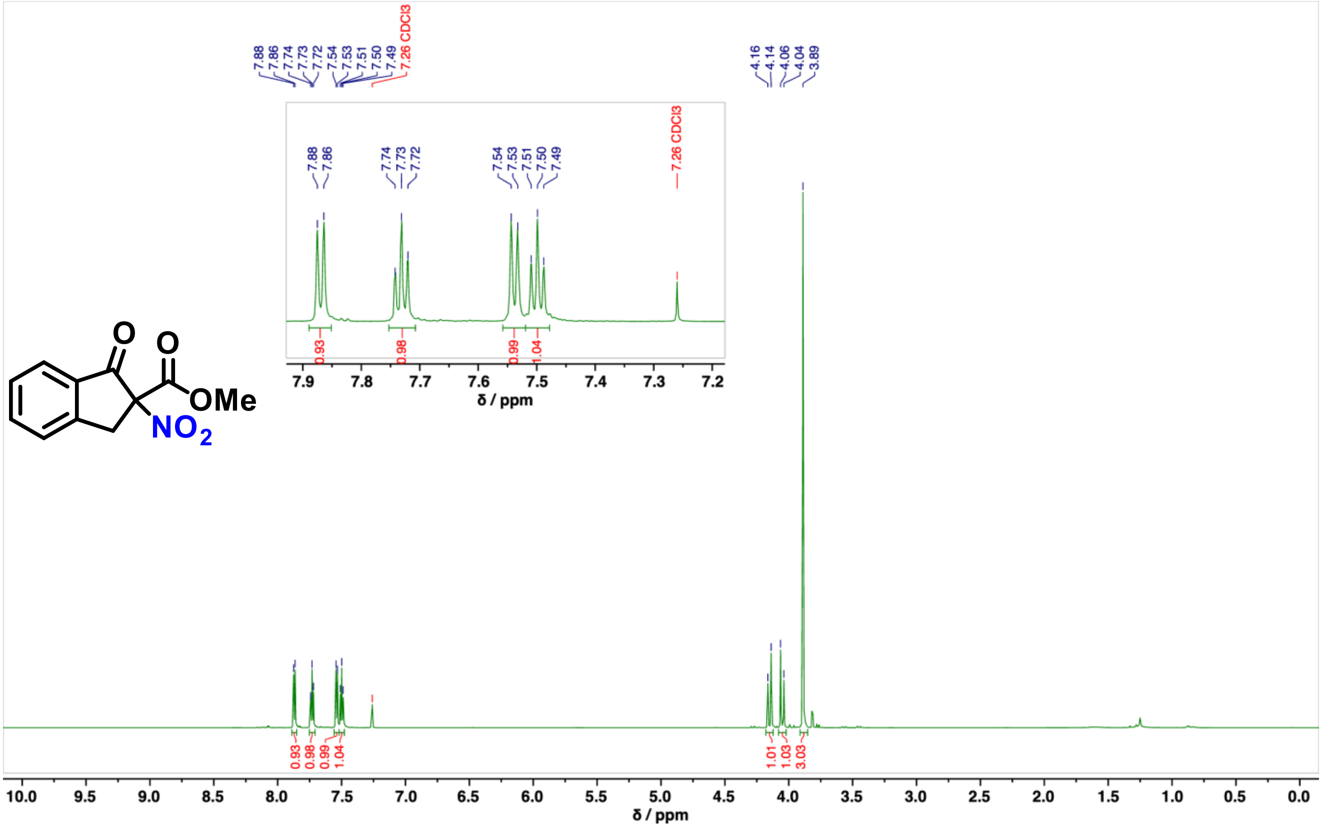

10b, ^{13}C-NMR (176 MHz, $CDCl_3$, 298 K, δ / ppm):

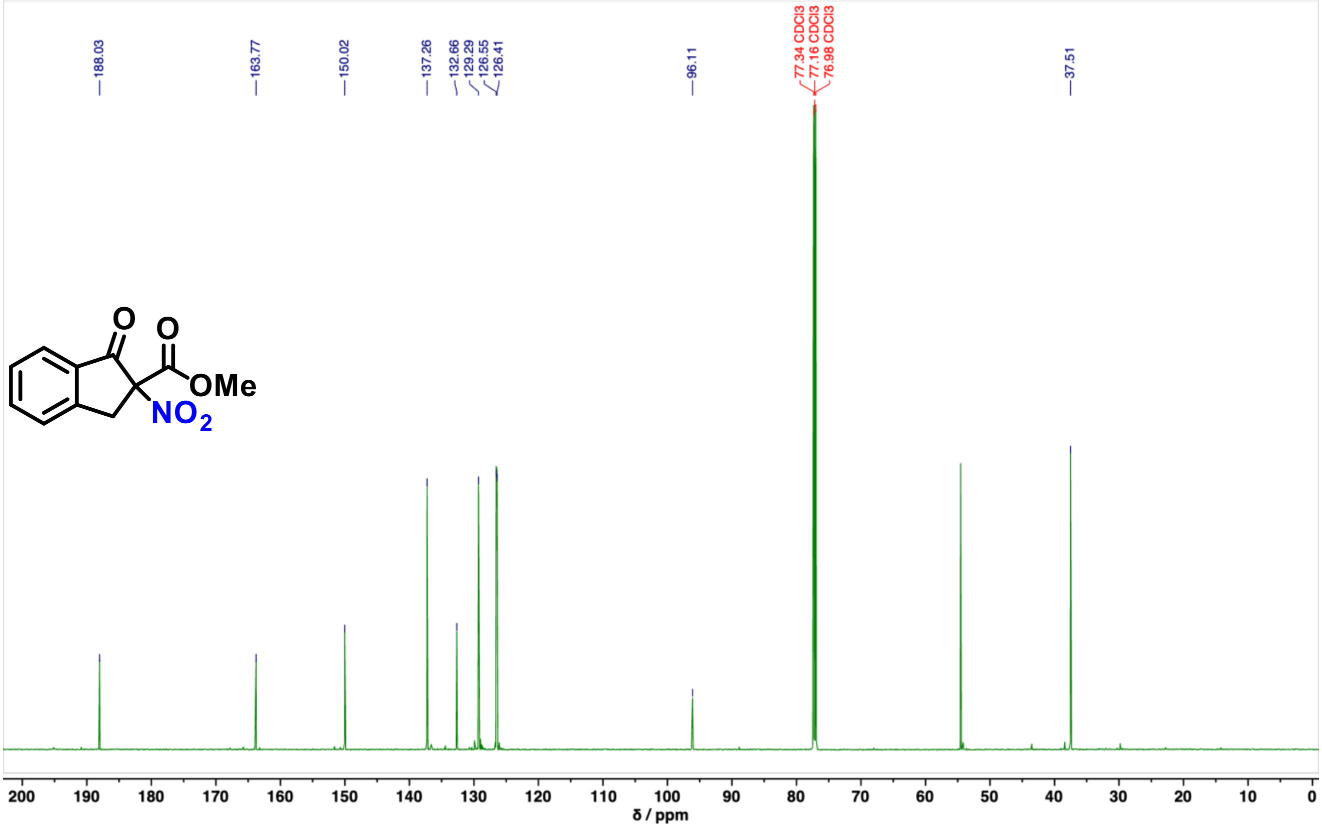

10c, ^{1}H-NMR (700 MHz, $CDCl_3$, 298 K, δ / ppm):

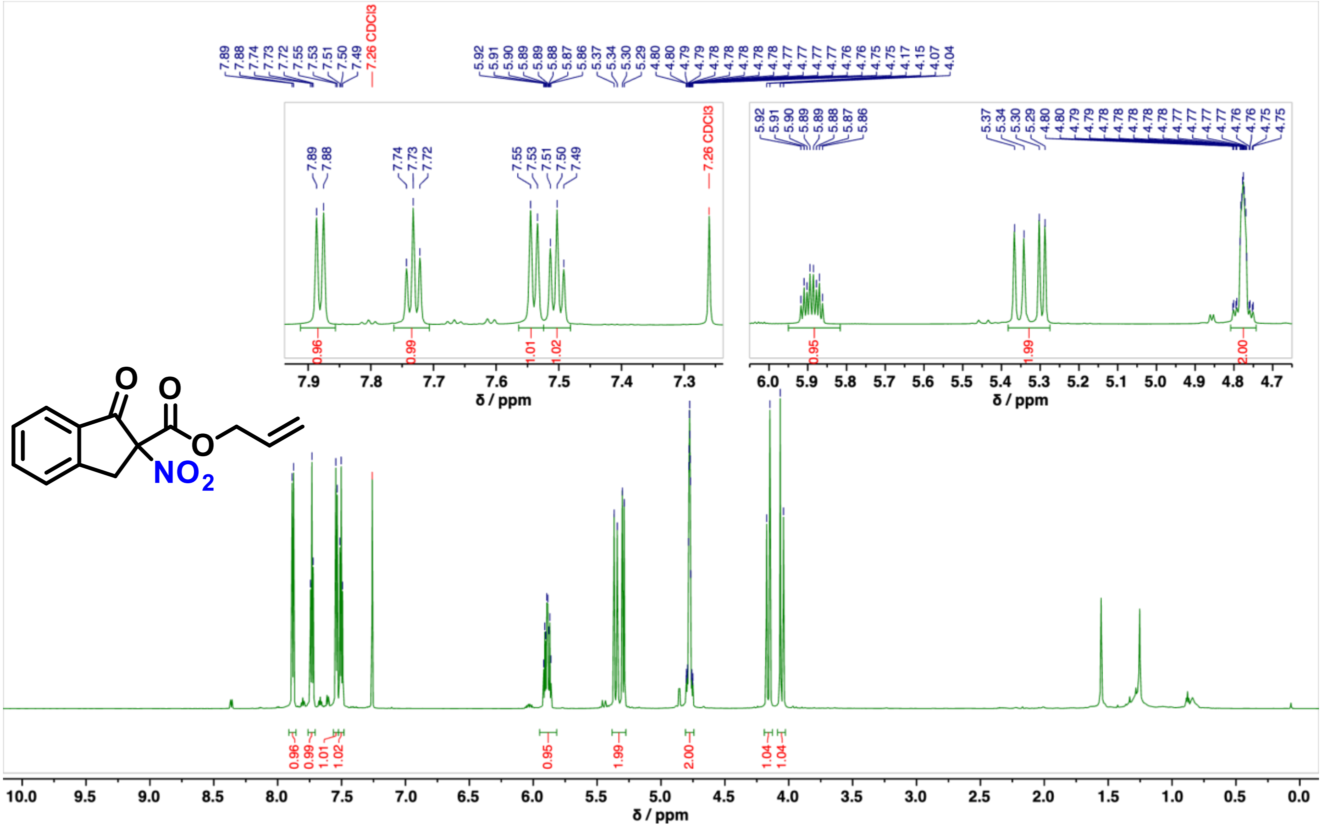

10c, ^{13}C-NMR (176 MHz, $CDCl_3$, 298 K, δ / ppm):

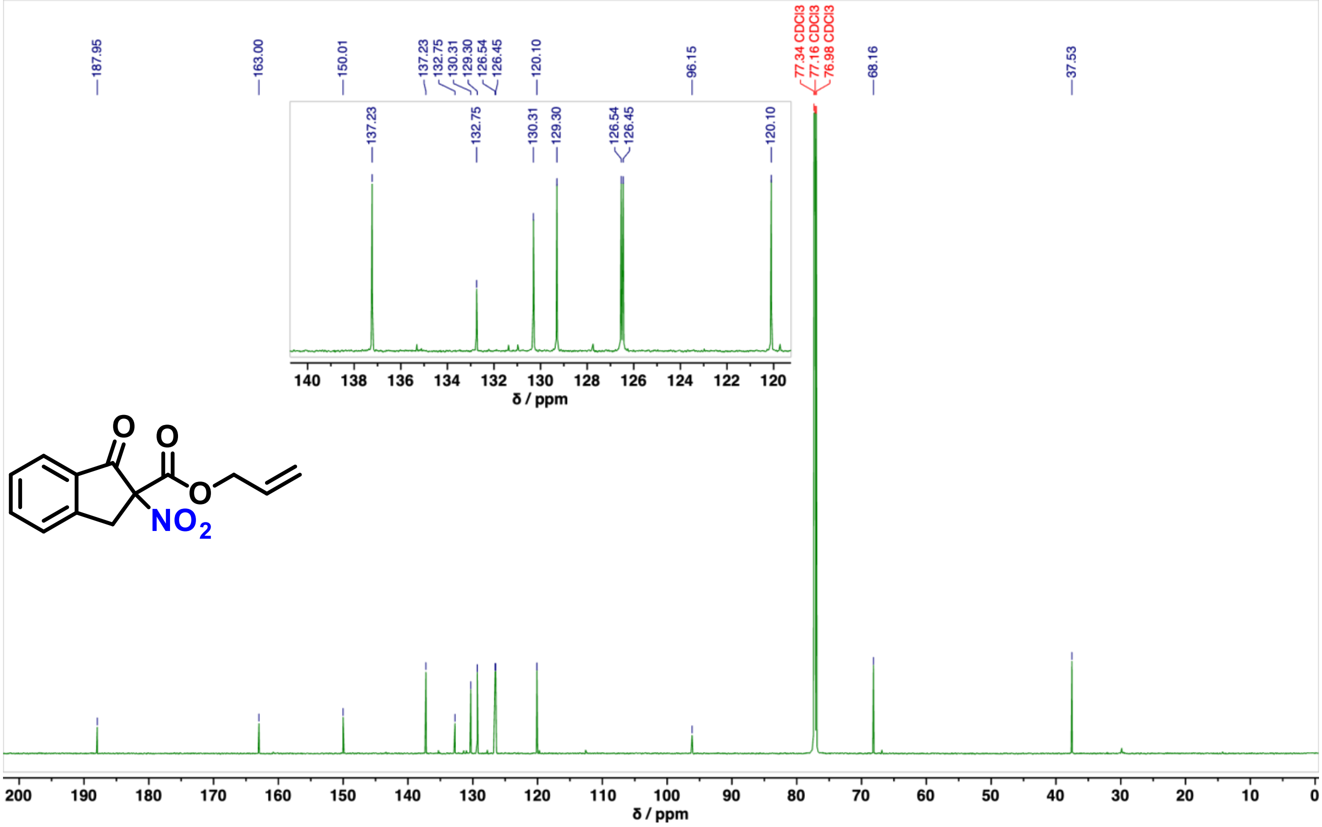

7. Infrared spectra

2a, IR (neat, FT-ATR, 298 K, $\bar{\nu}$ / cm^{-1}):

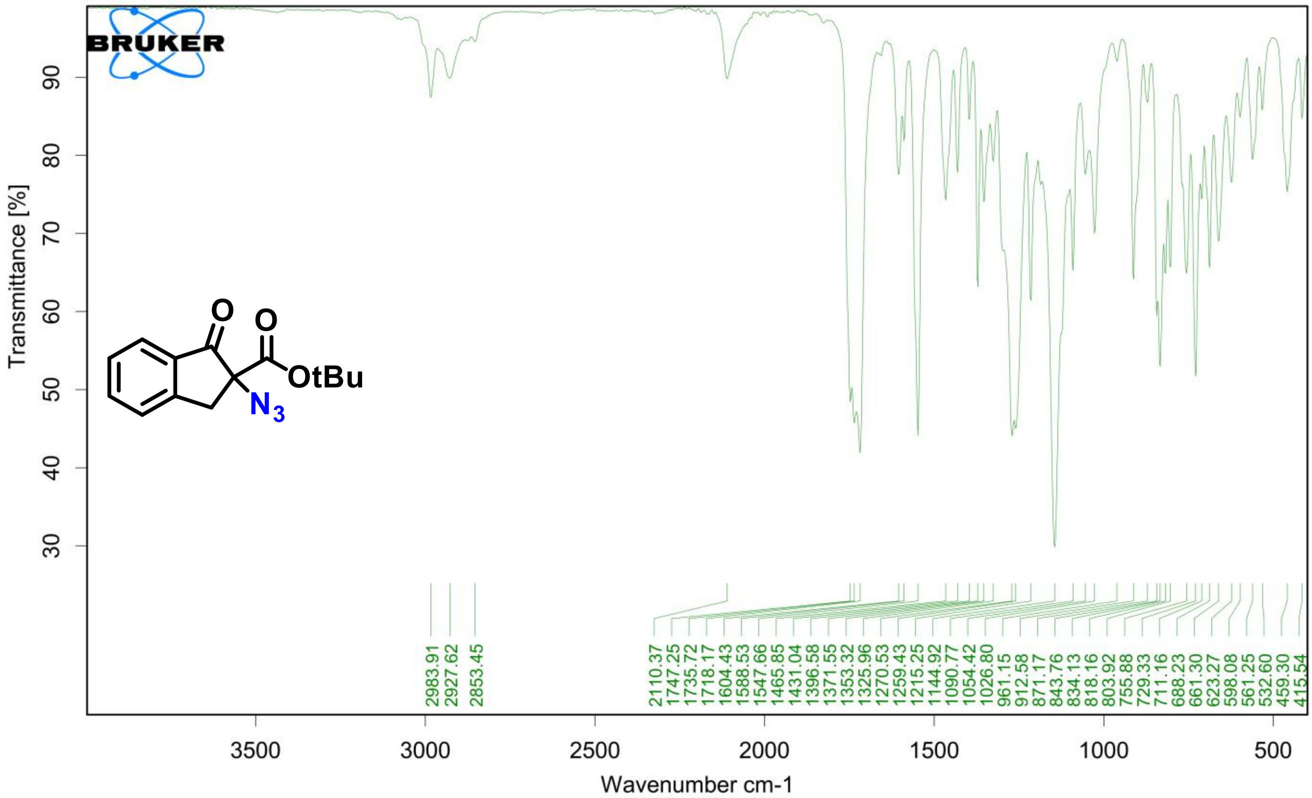

2b, IR (neat, FT-ATR, 298 K, $\bar{\nu}$ / cm^{-1}):

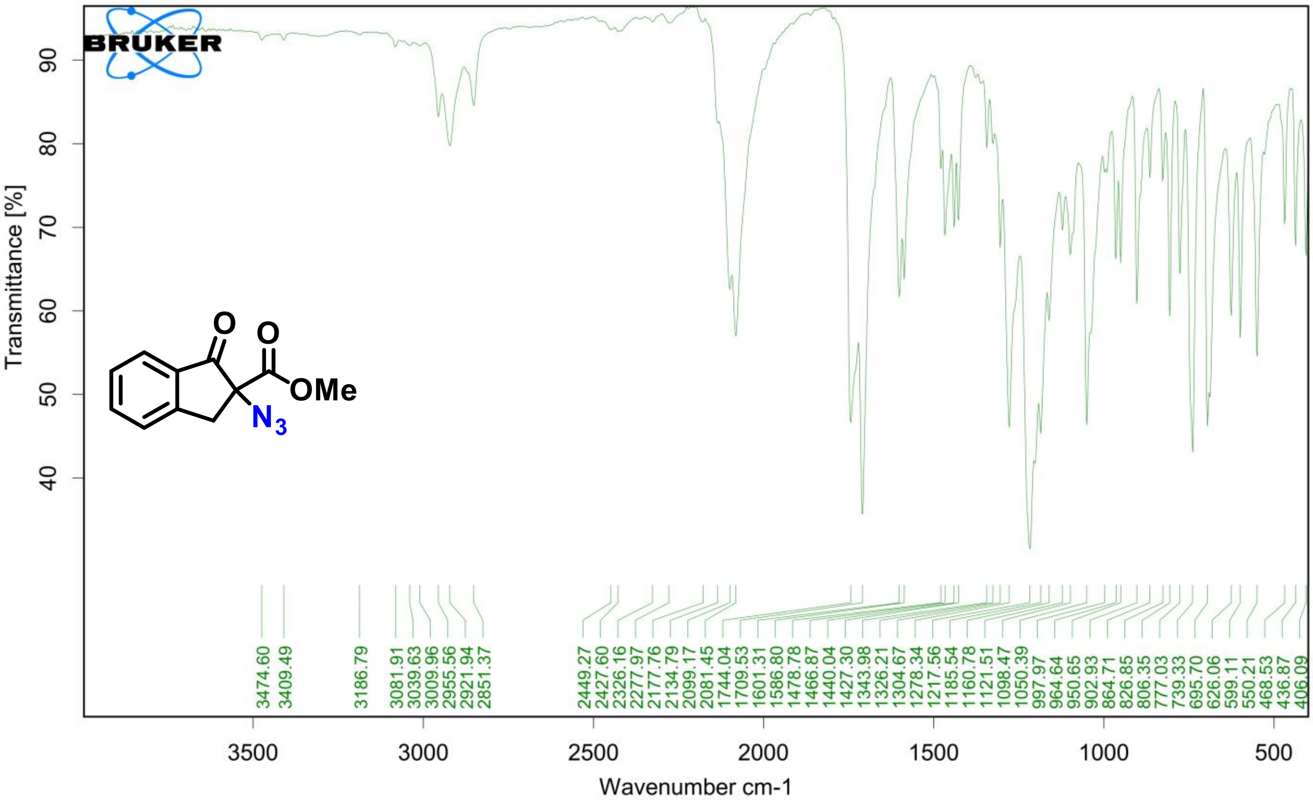

2c, IR (neat, FT-ATR, 298 K, $\bar{\nu}$ / cm^{-1}):

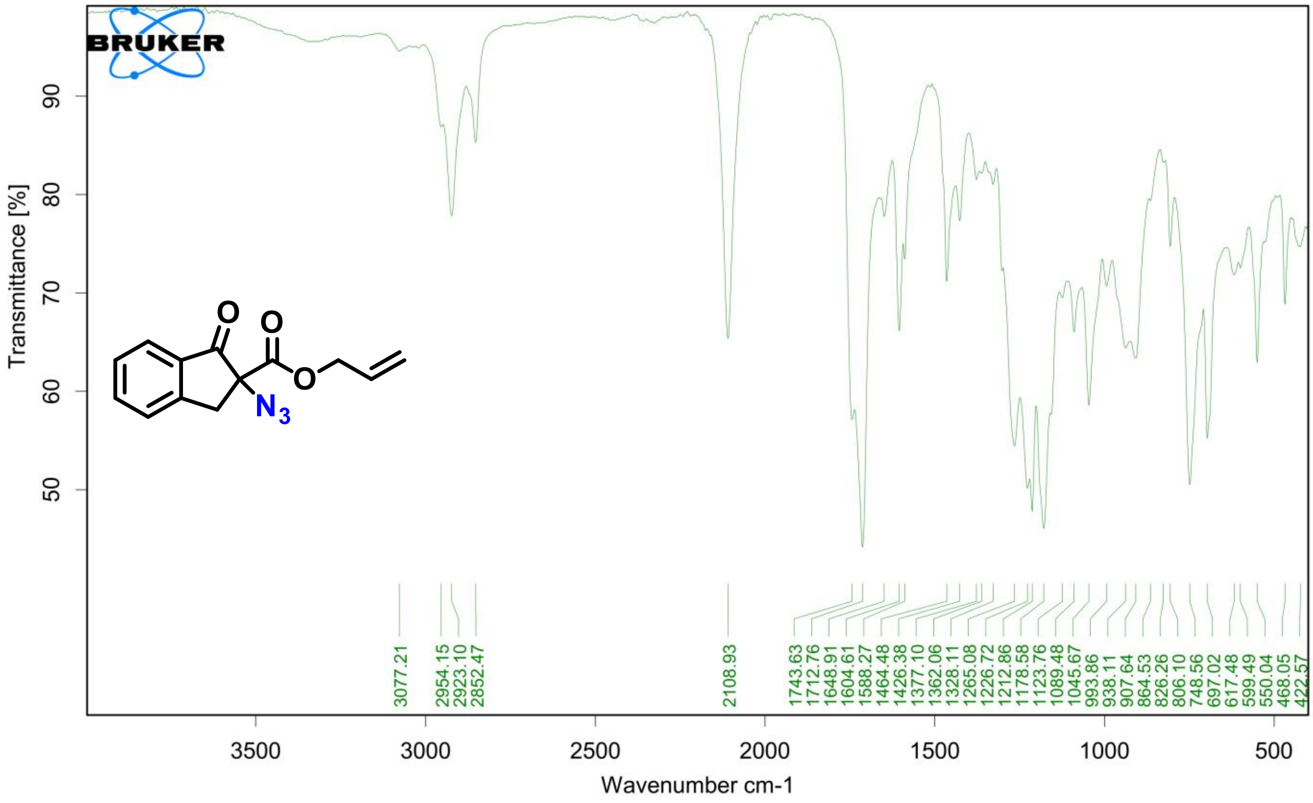

2d, IR (neat, FT-ATR, 298 K, $\bar{\nu}$ / cm^{-1}):

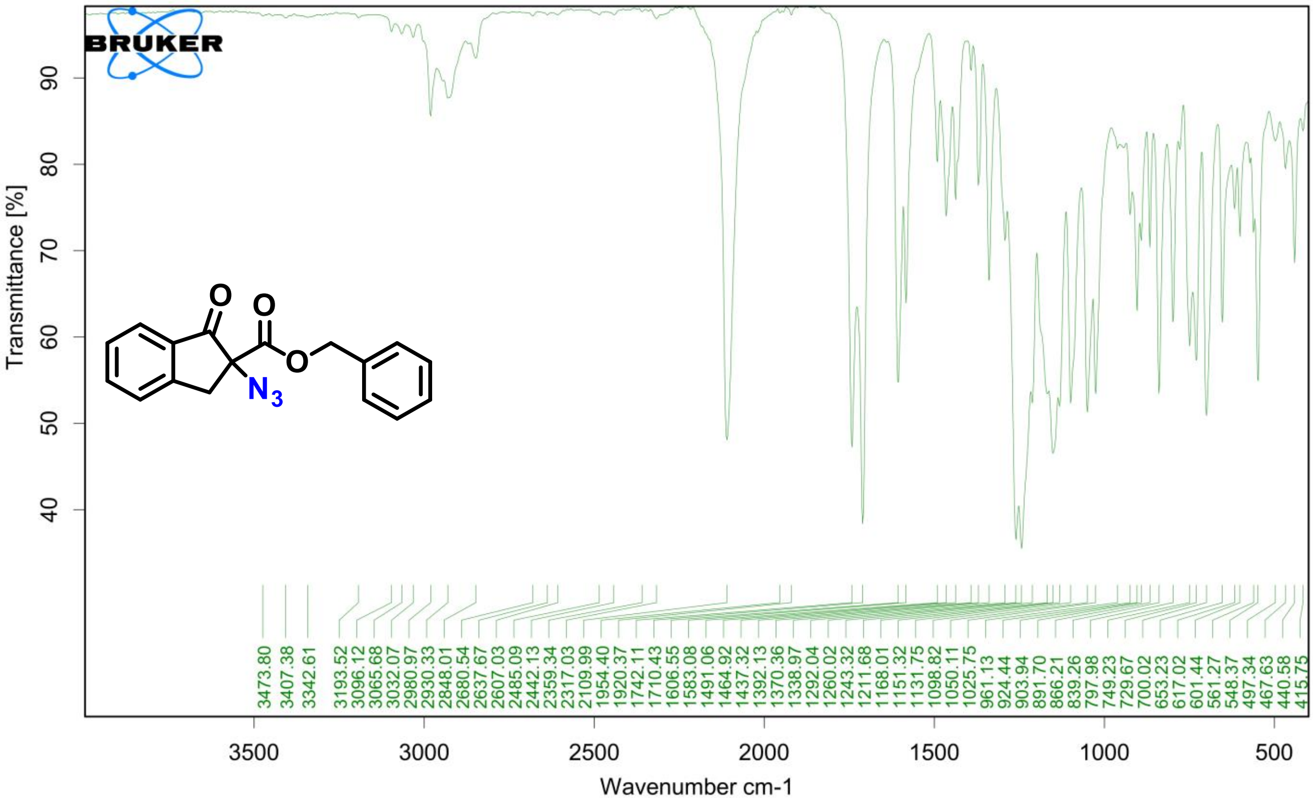

2e, IR (neat, FT-ATR, 298 K, $\bar{\nu}$ / cm^{-1}):

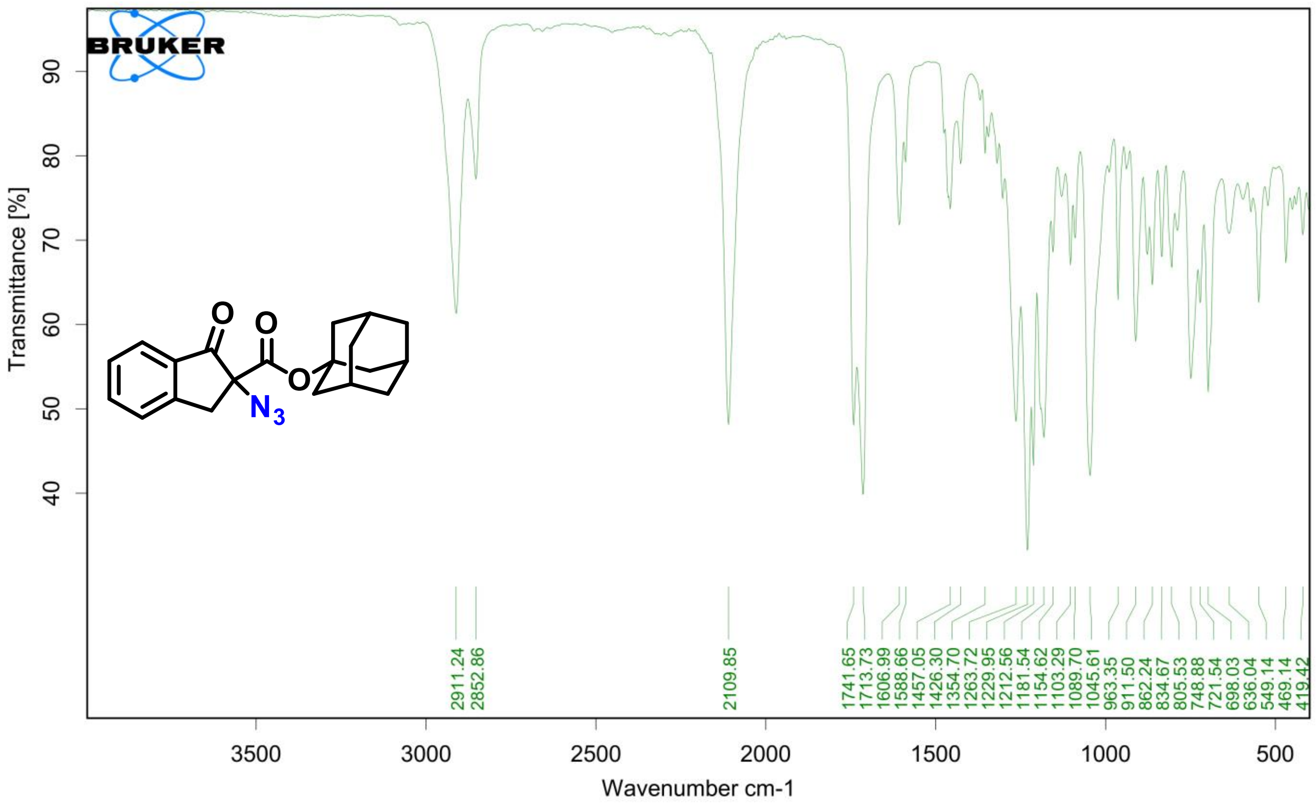

2f, IR (neat, FT-ATR, 298 K, $\bar{\nu}$ / cm^{-1}):

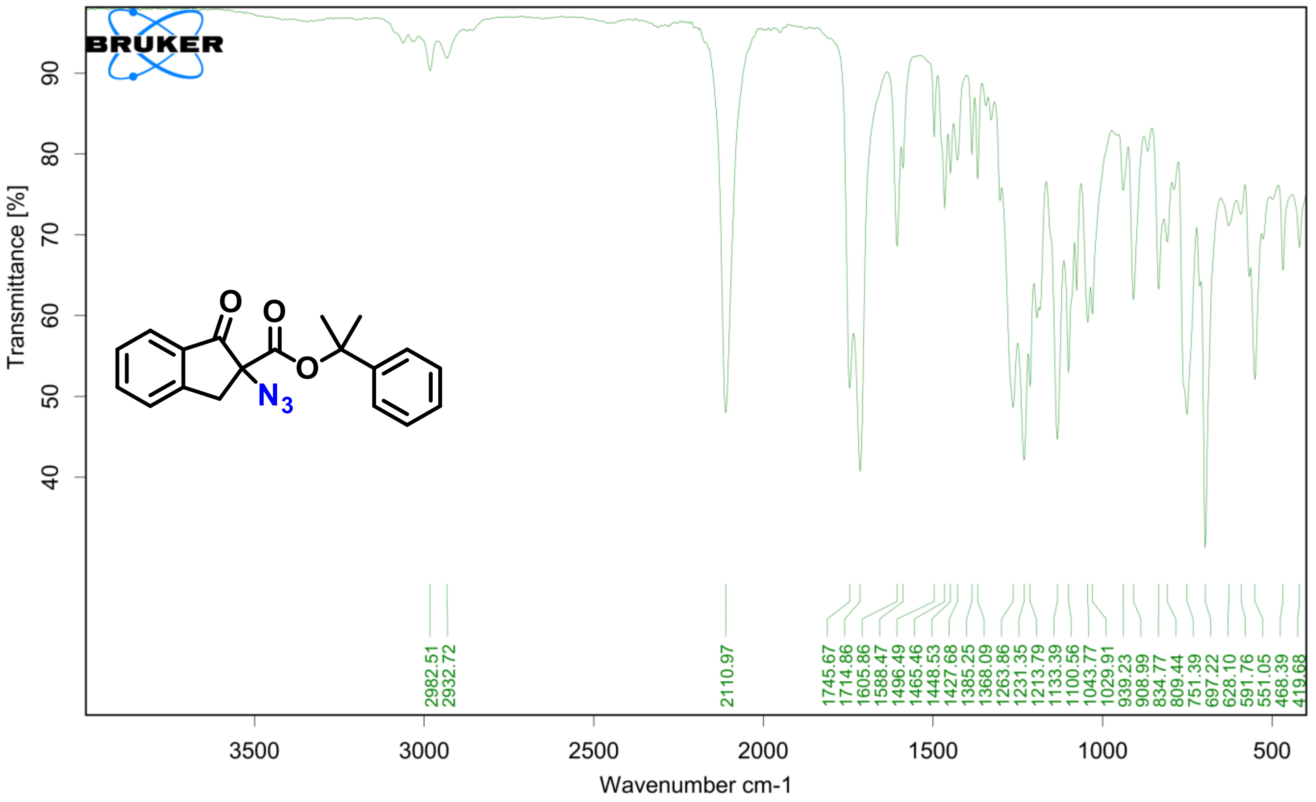

2g, IR (neat, FT-ATR, 298 K, $\bar{\nu}$ / cm^{-1}):

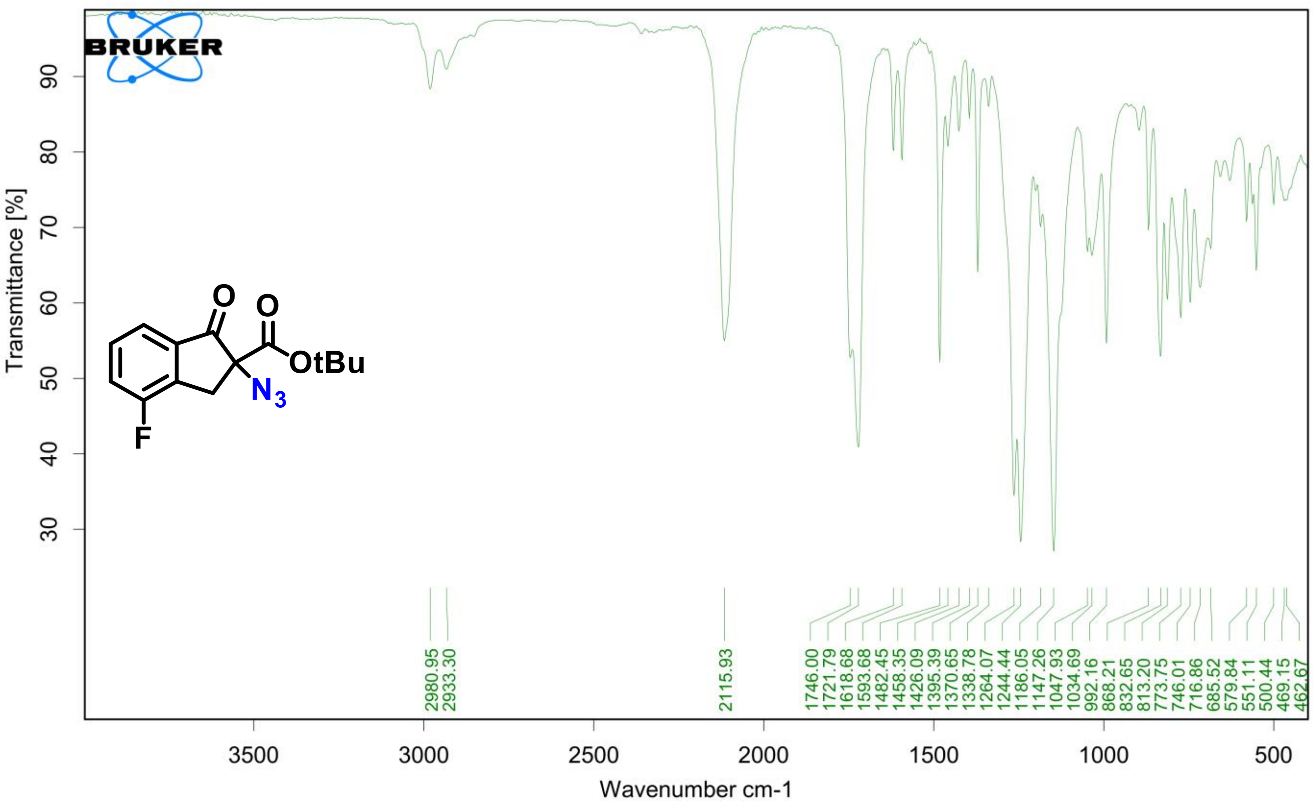

2h, IR (neat, FT-ATR, 298 K, $\bar{\nu}$ / cm^{-1}):

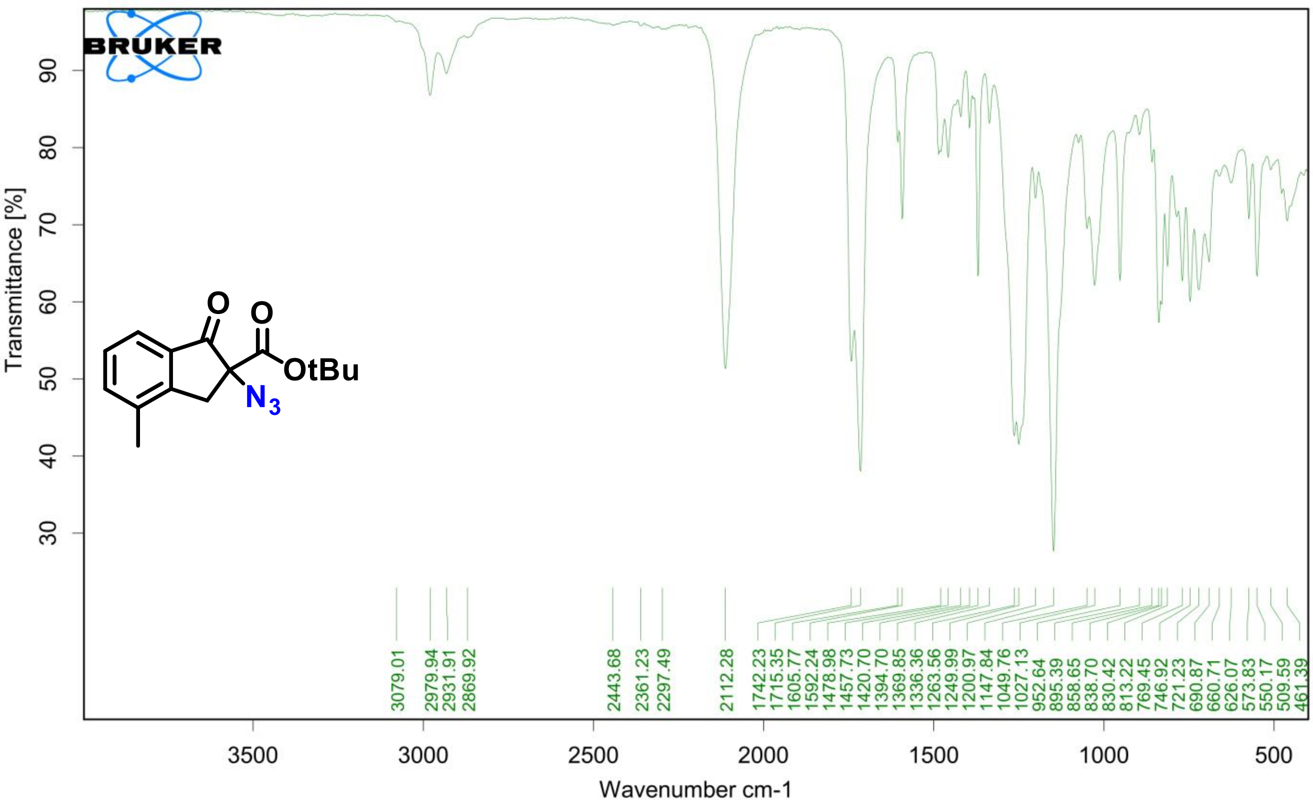

2i, IR (neat, FT-ATR, 298 K, $\bar{\nu}$ / cm^{-1}):

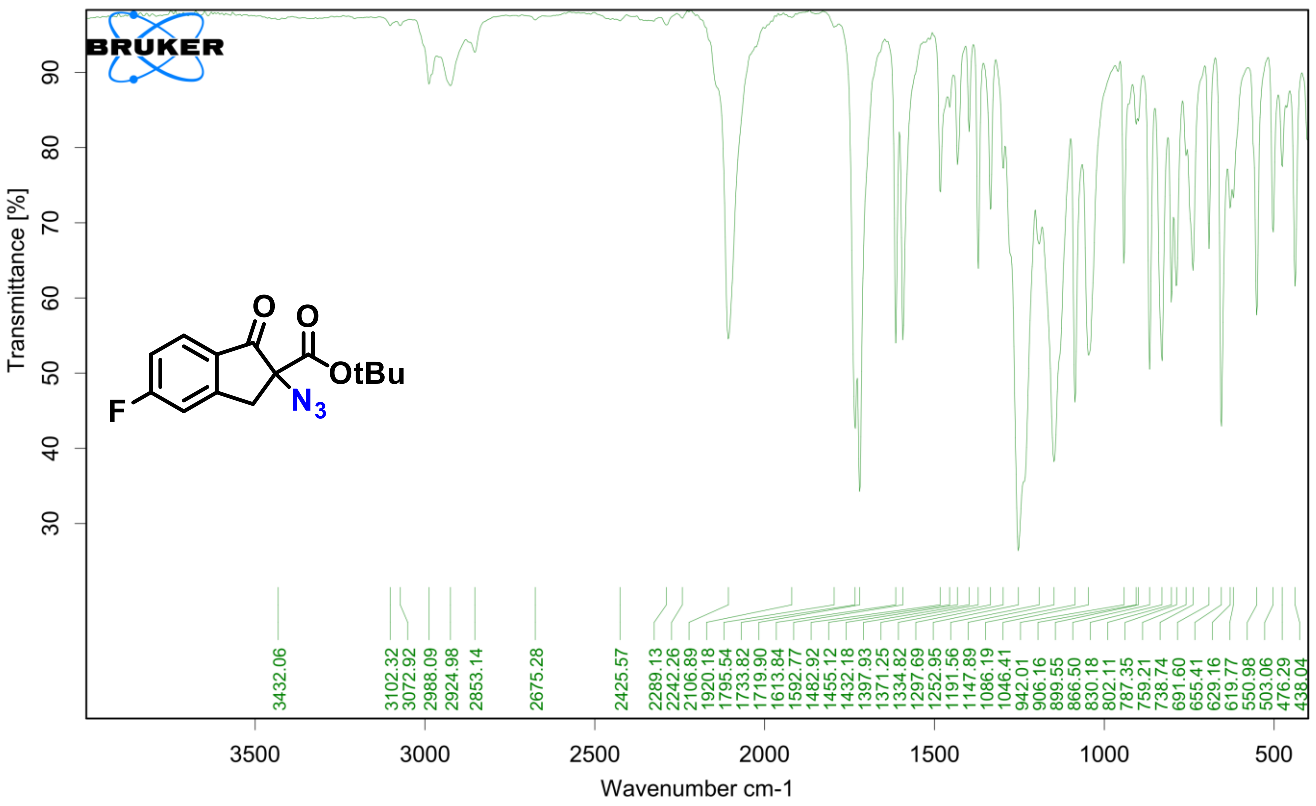

2j, IR (neat, FT-ATR, 298 K, $\bar{\nu}$ / cm^{-1}):

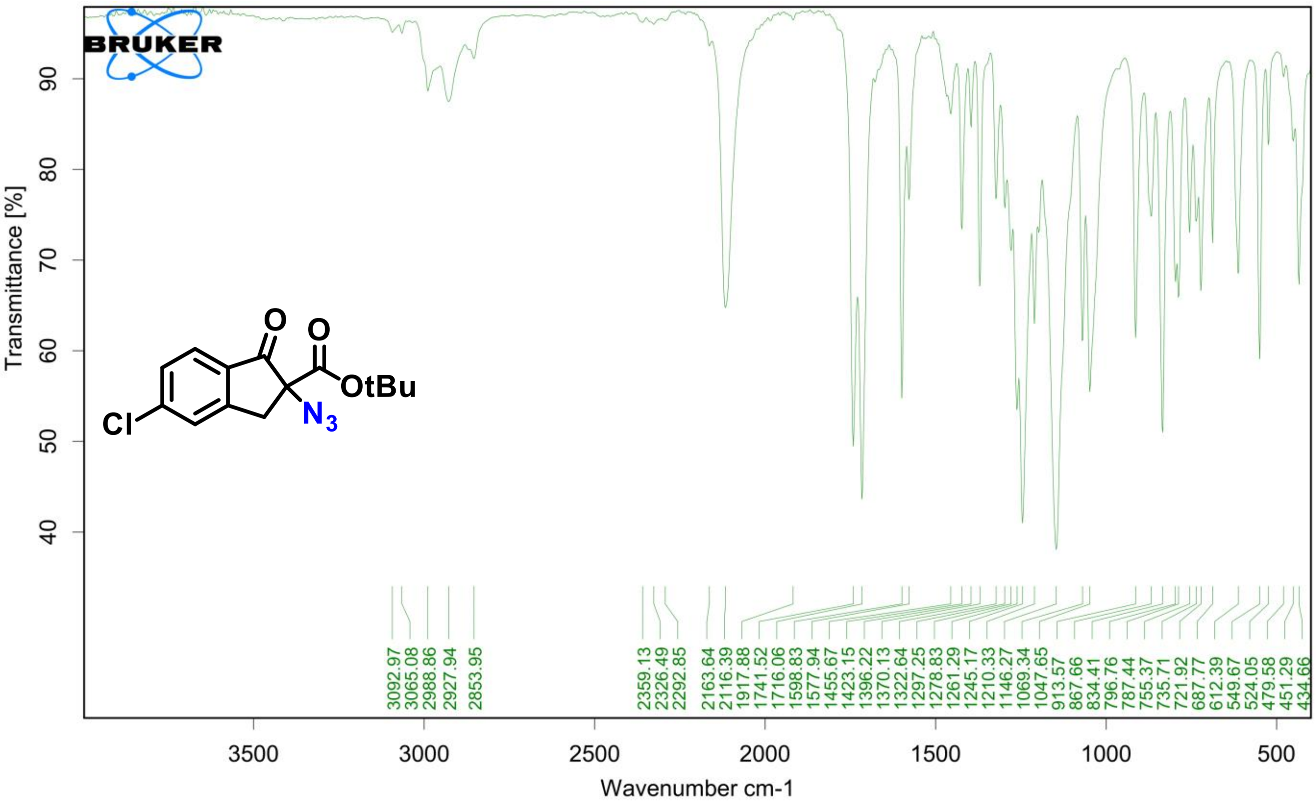

2k, IR (neat, FT-ATR, 298 K, $\bar{\nu}$ / cm^{-1}):

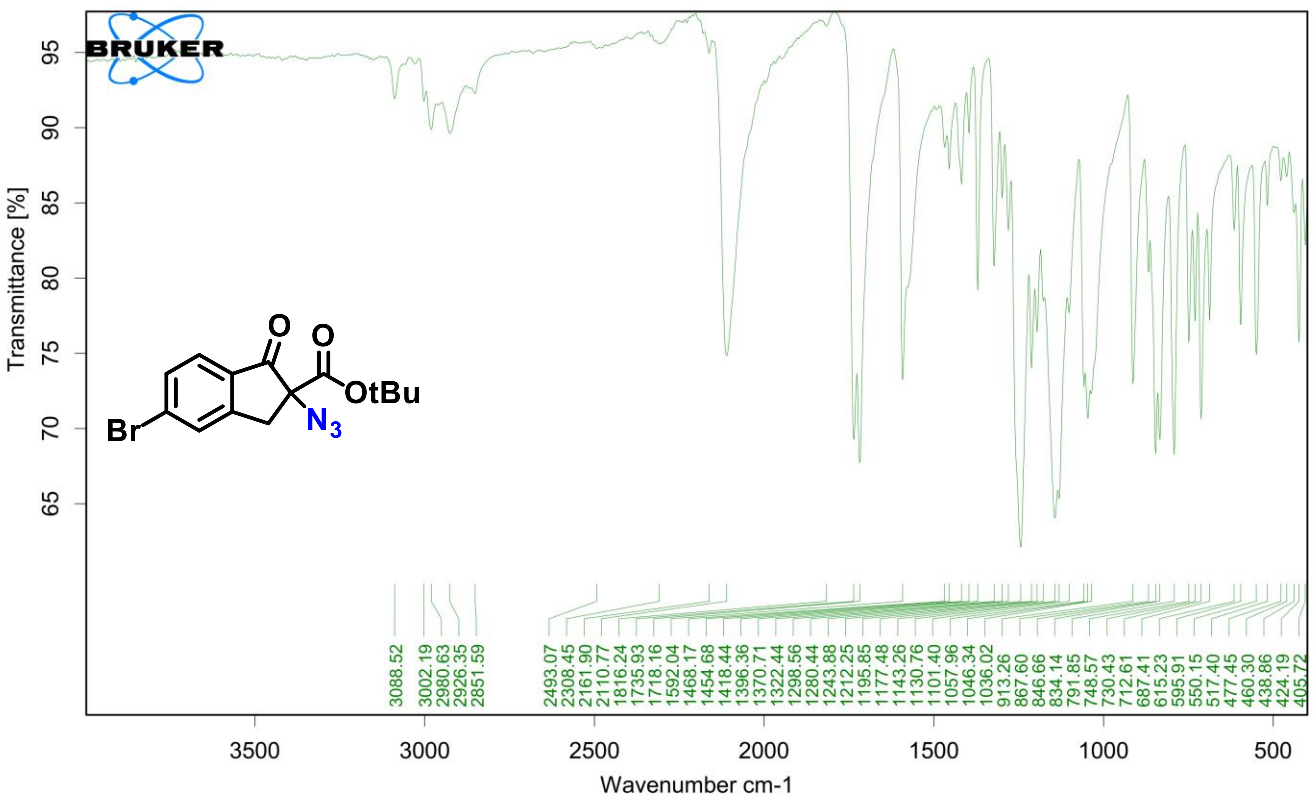

2l, IR (neat, FT-ATR, 298 K, $\bar{\nu}$ / cm^{-1}):

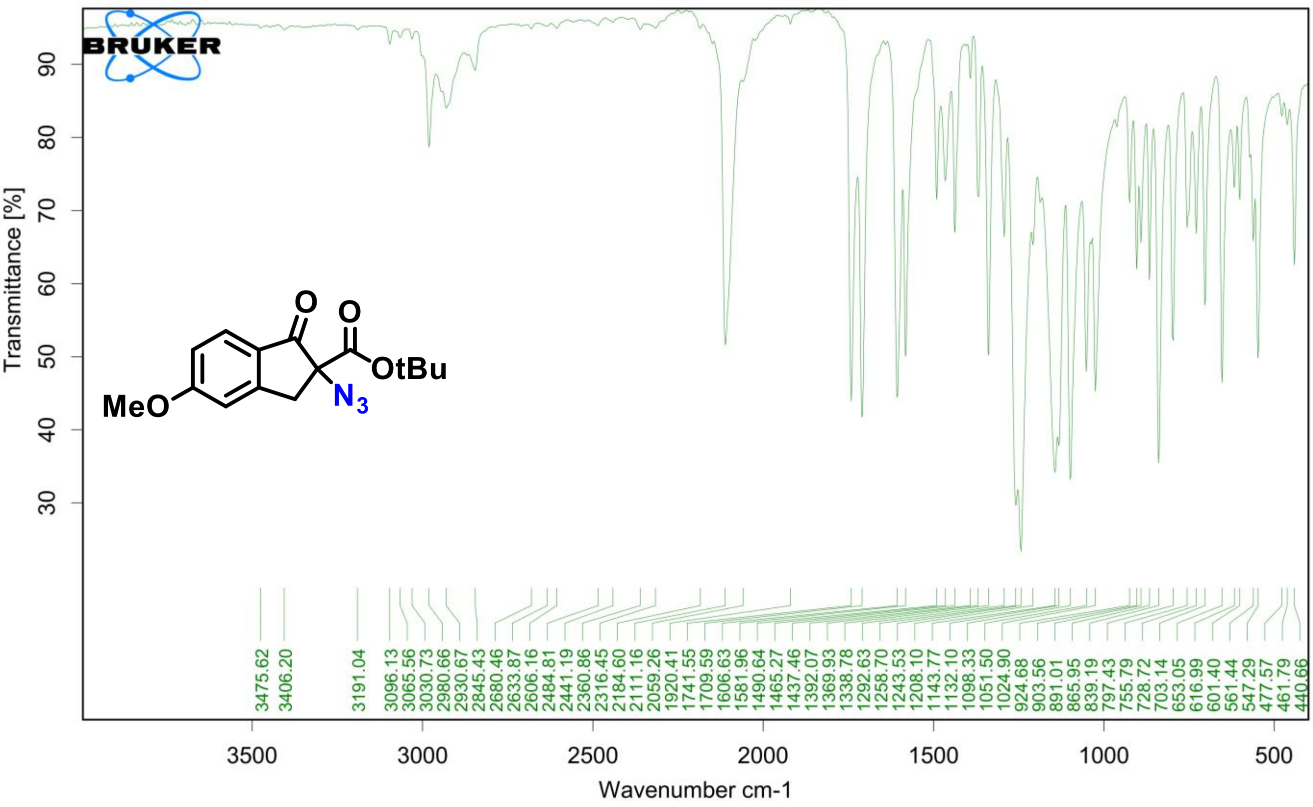

2m, IR (neat, FT-ATR, 298 K, $\bar{\nu}$ / cm^{-1}):

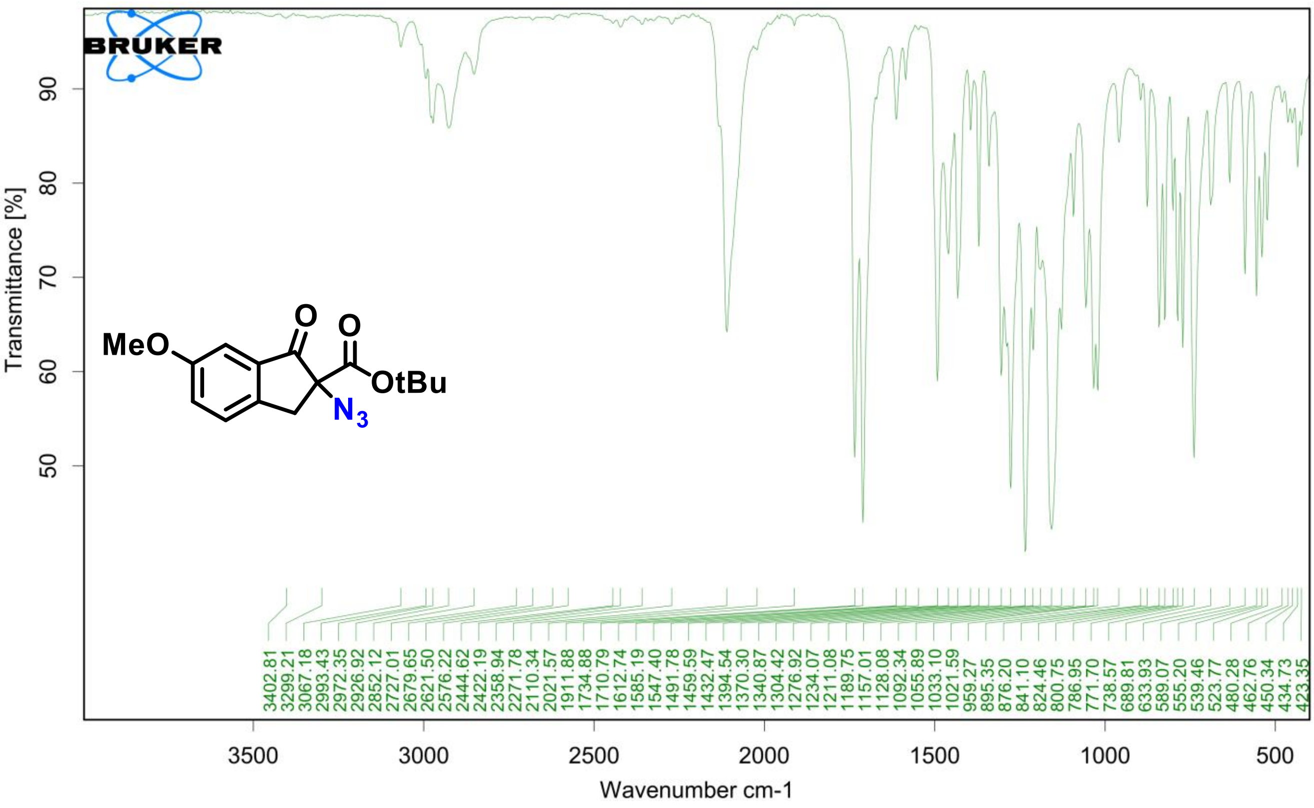

2n, IR (neat, FT-ATR, 298 K, $\bar{\nu}$ / cm^{-1}):

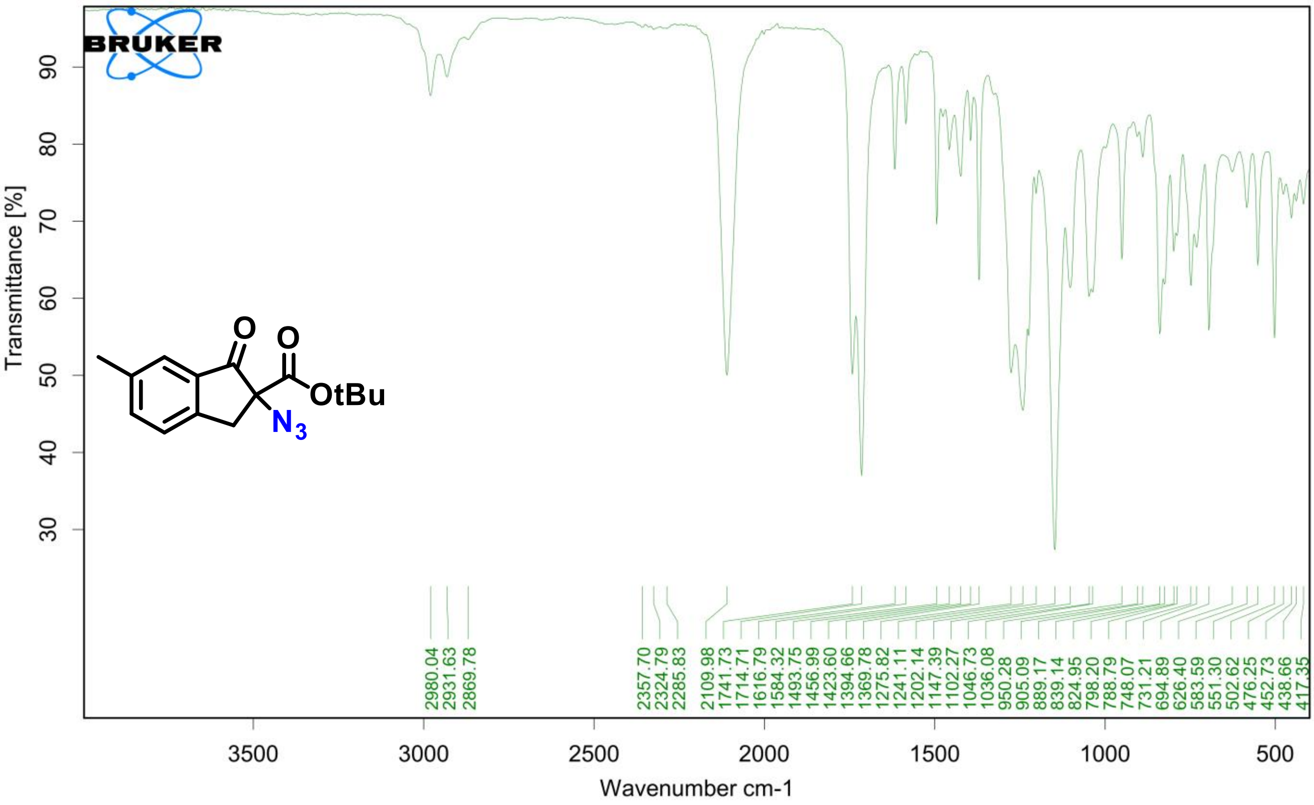

5a, IR (neat, FT-ATR, 298 K, $\bar{\nu}$ / cm^{-1}):

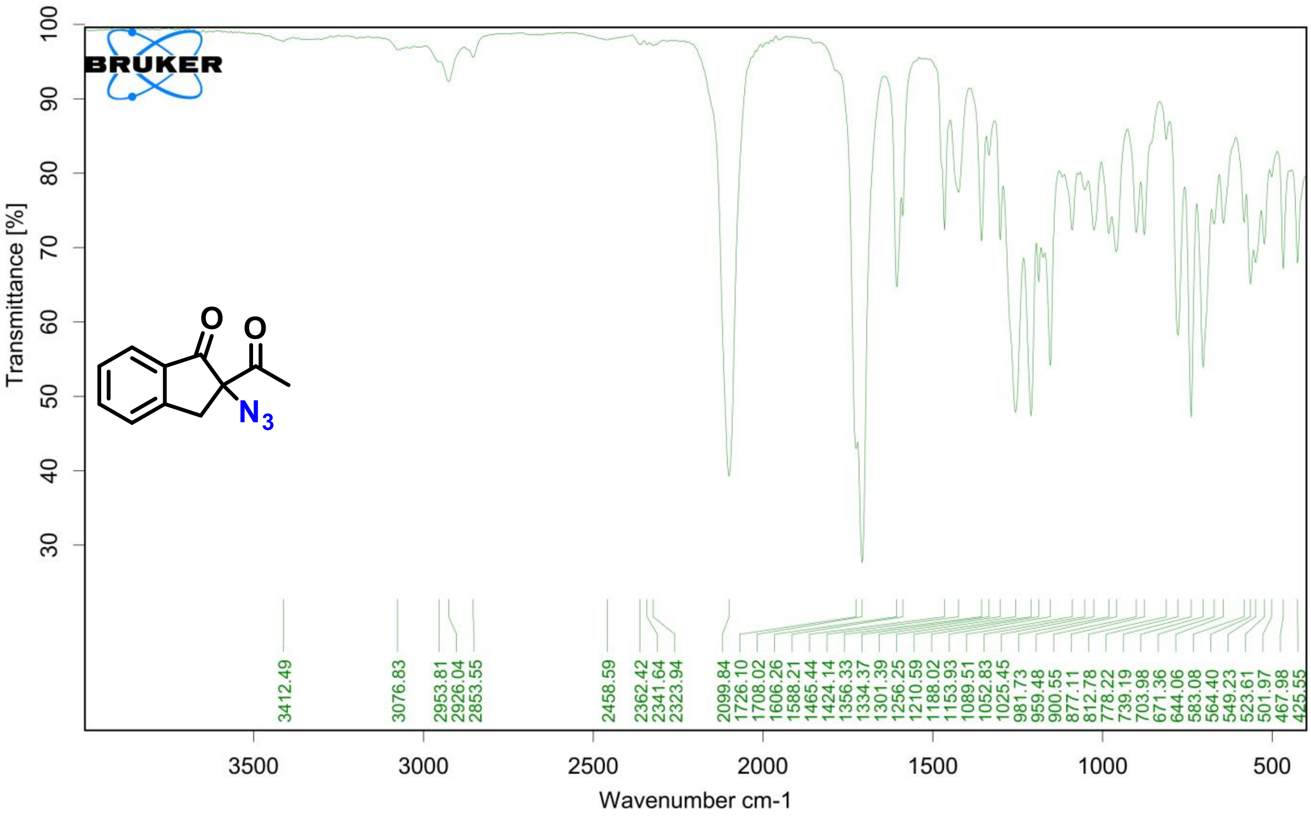

5b, IR (neat, FT-ATR, 298 K, $\bar{\nu}$ / cm^{-1}):

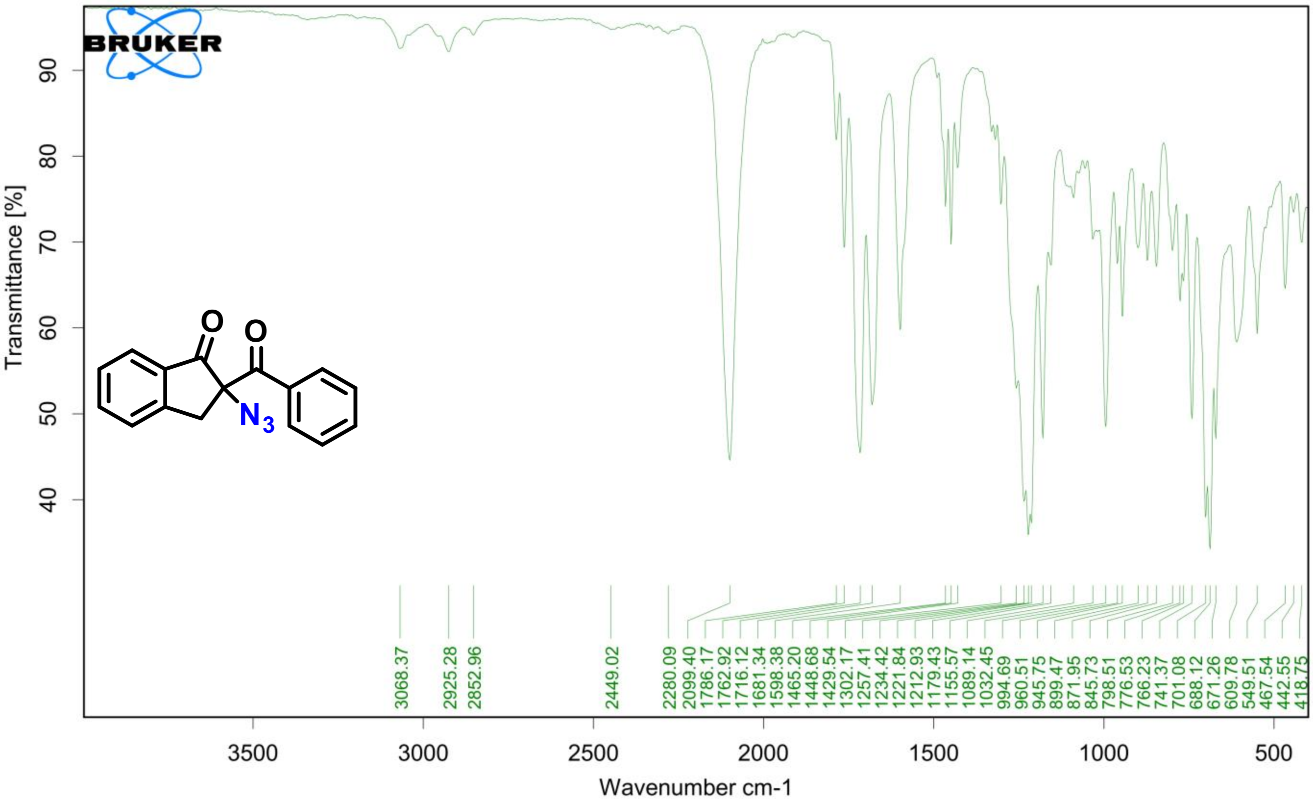

6, IR (neat, FT-ATR, 298 K, $\bar{\nu}$ / cm^{-1}):

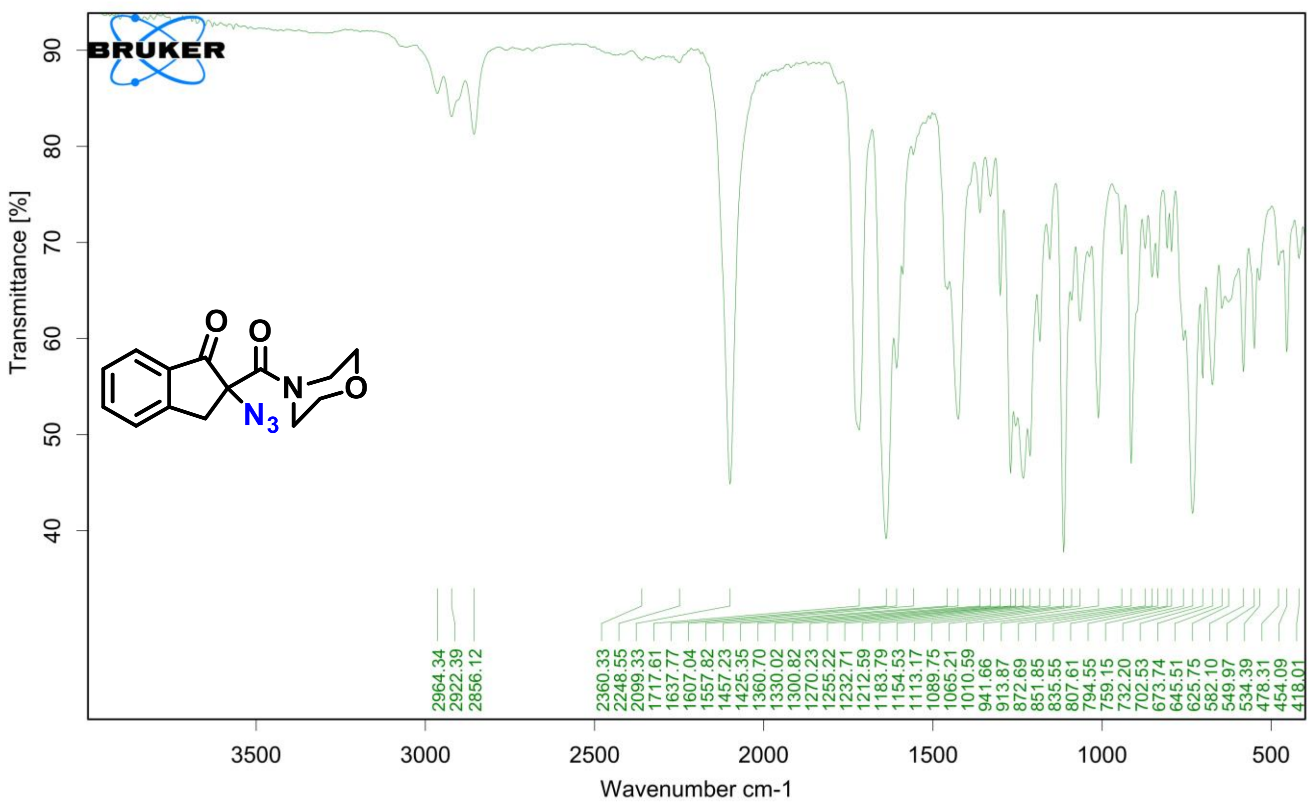

7a, IR (neat, FT-ATR, 298 K, $\bar{\nu}$ / cm^{-1}):

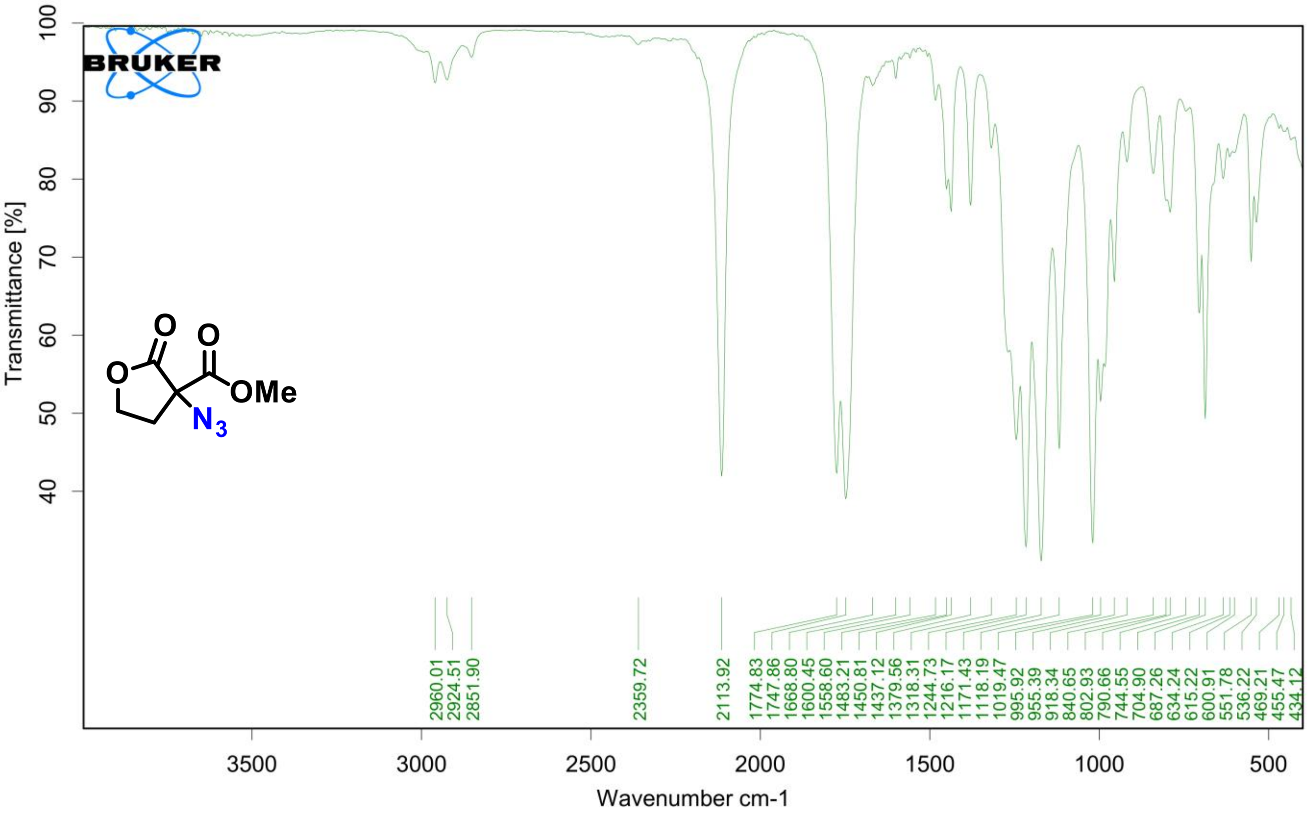

7b, IR (neat, FT-ATR, 298 K, $\bar{\nu}$ / cm^{-1}):

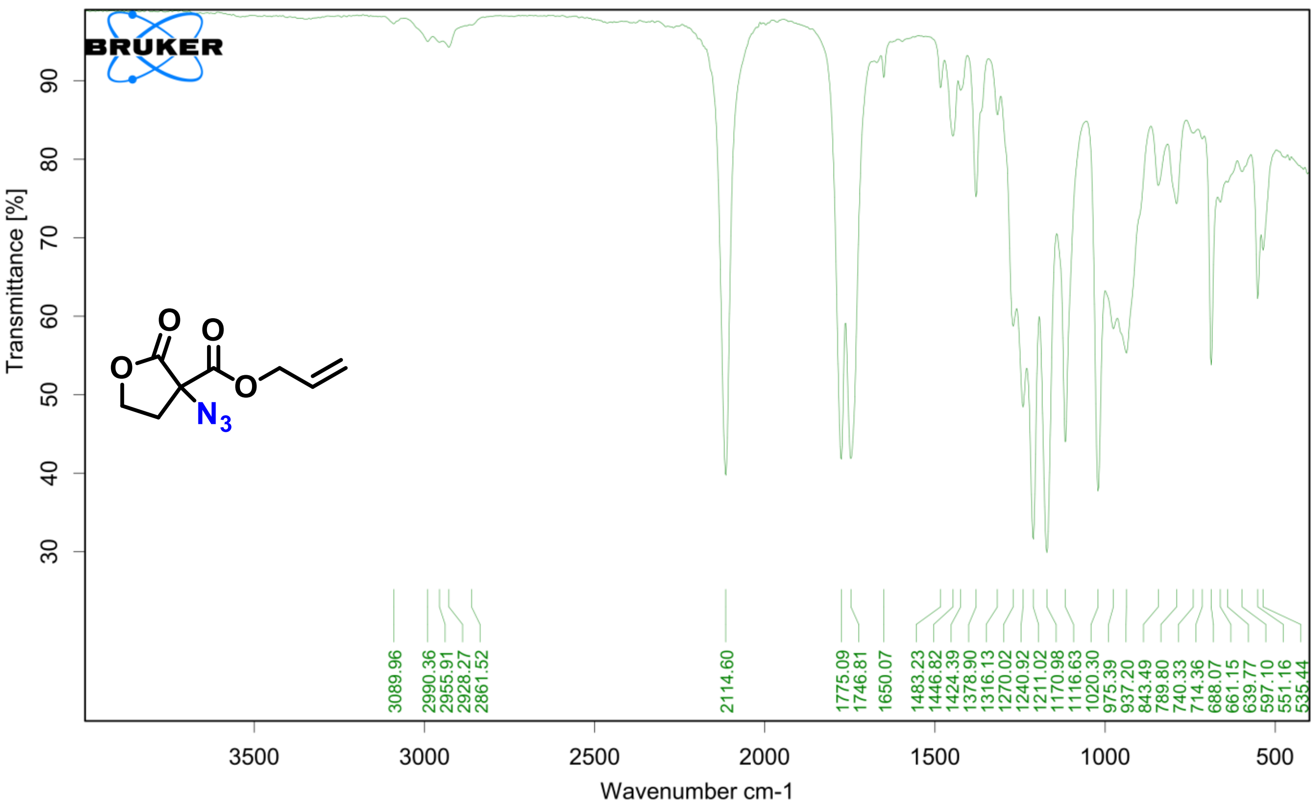

7c, IR (neat, FT-ATR, 298 K, $\bar{\nu}$ / cm^{-1}):

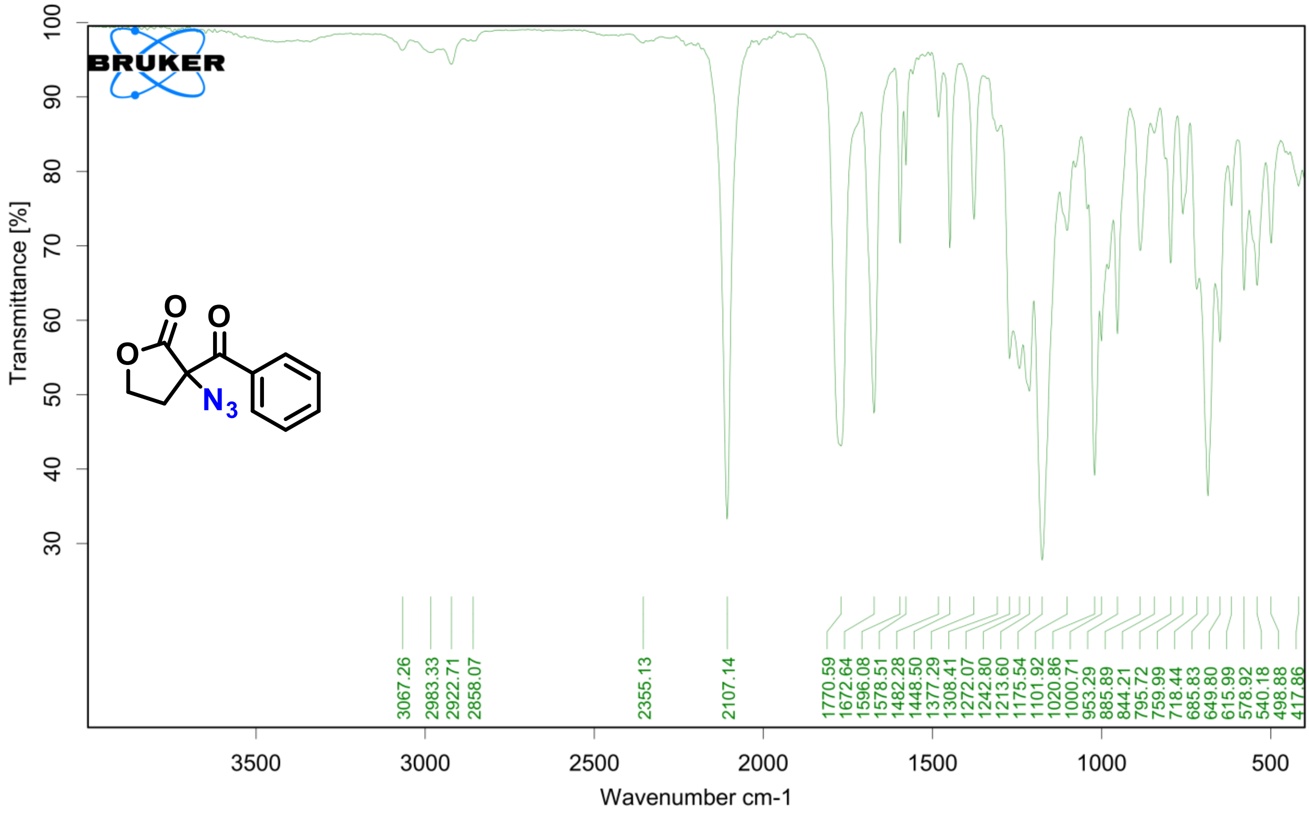

10a, IR (neat, FT-ATR, 298 K, $\bar{\nu}$ / cm^{-1}):

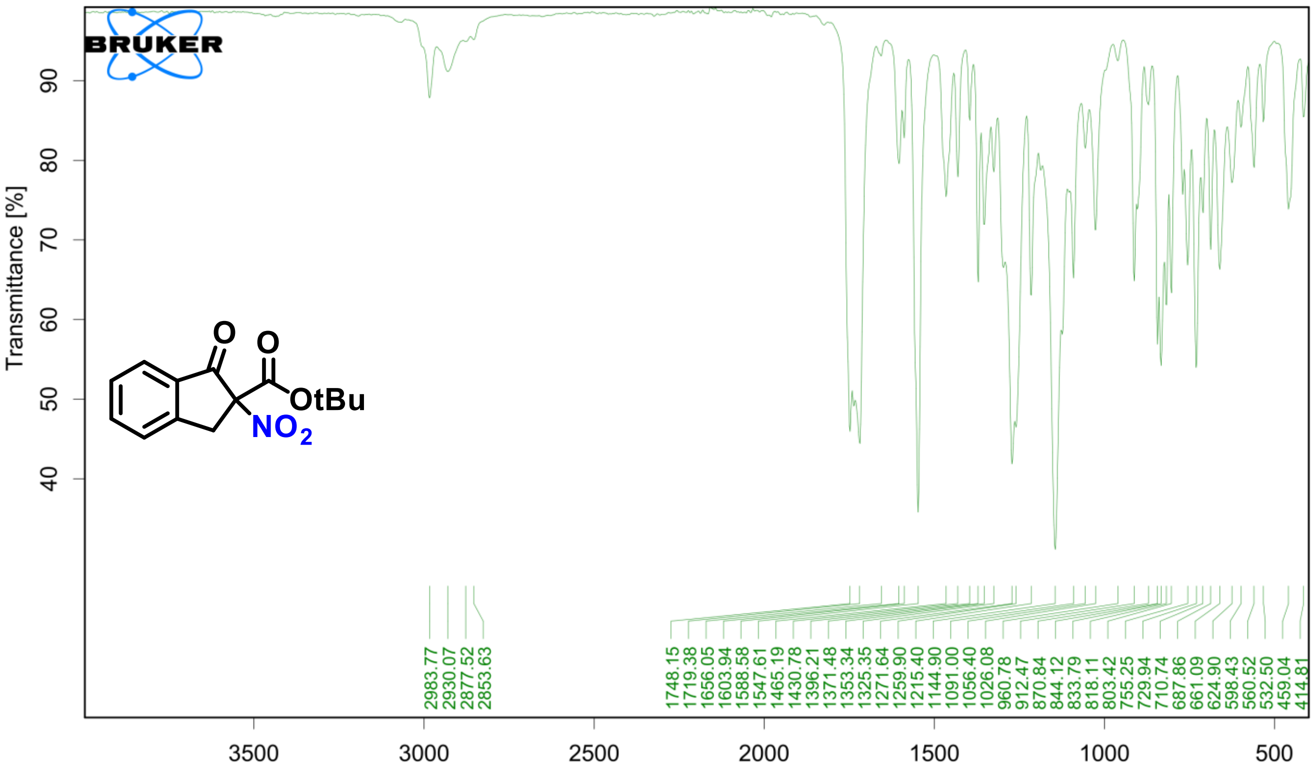

10b, IR (neat, FT-ATR, 298 K, $\bar{\nu}$ / cm^{-1}):

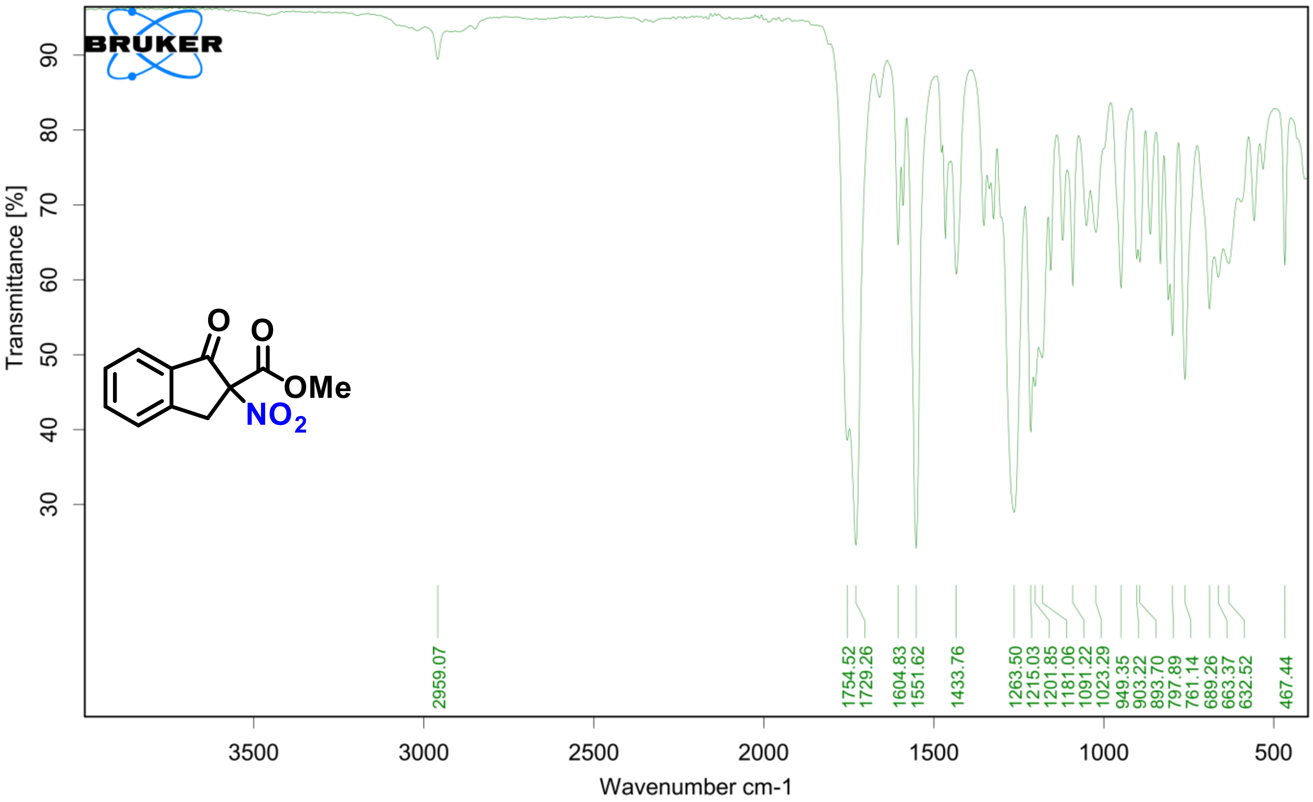

10c, IR (neat, FT-ATR, 298 K, $\bar{\nu}$ / cm^{-1}):

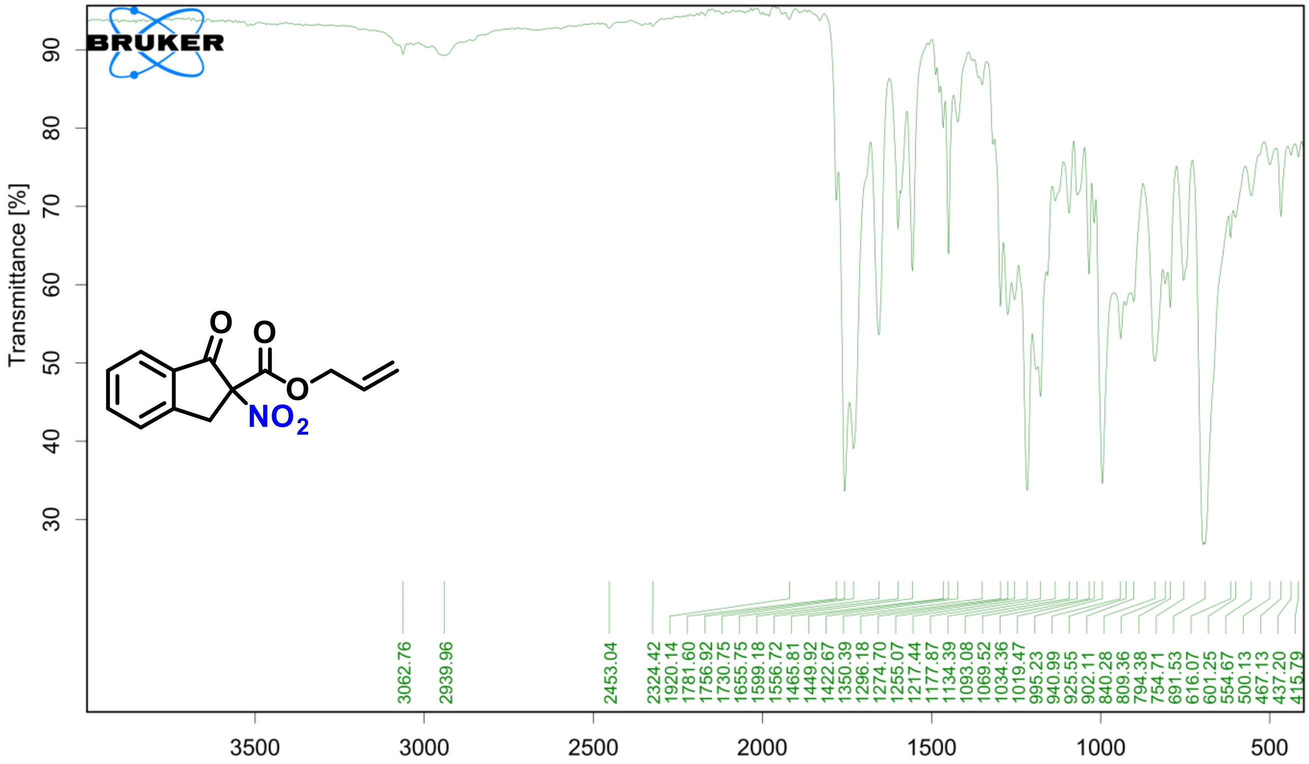

8. High-resolution mass spectra

(major only)

2a, HRMS (ESI$^+$-QqTOF, *m/z*):

calculated for $C_{14}H_{19}N_4O_3$ $[M+NH_4]^+$: 291.1452, found: 291.1452 (0.00 ppm)

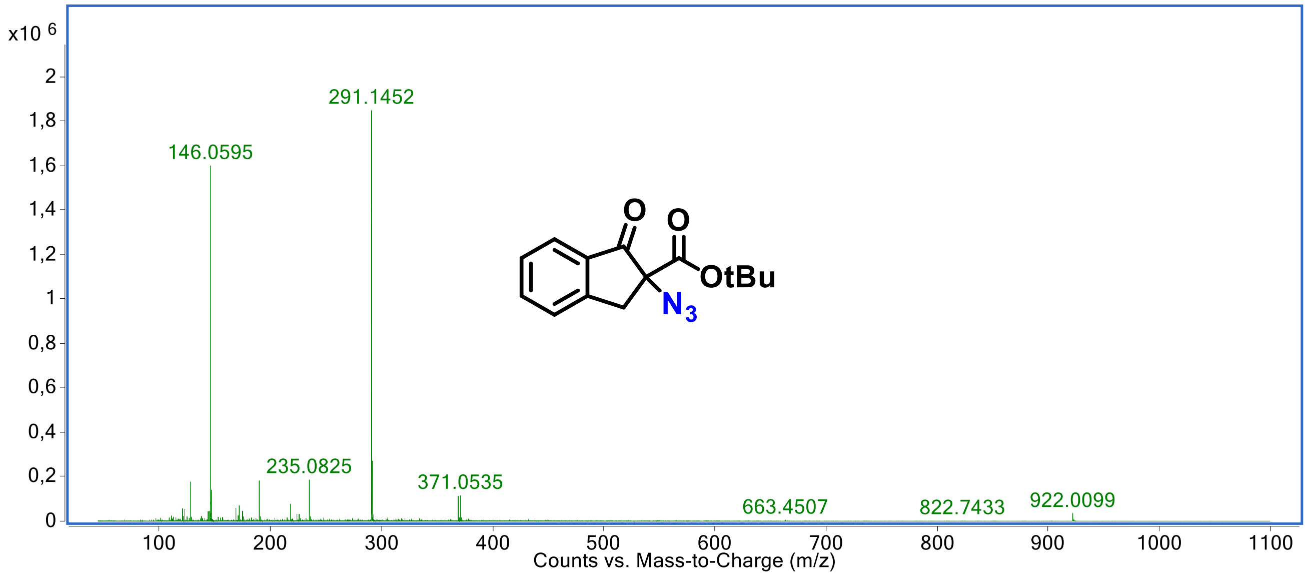

2b, HRMS (ESI$^+$-QqTOF, *m/z*):

calculated for $C_{11}H_{13}N_4O_3$ $[M+NH_4]^+$: 249.0982, found: 249.0982 (0.00 ppm)

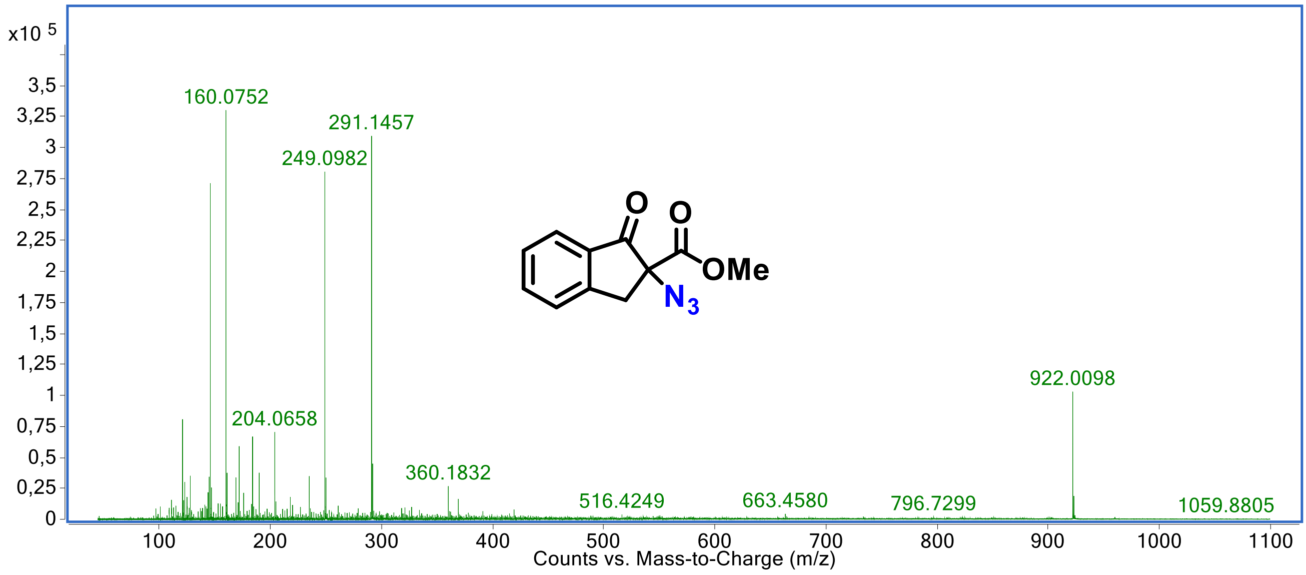

2c, HRMS (ESI$^+$-QqTOF, *m/z*):

calculated for $C_{13}H_{15}N_4O_3Na$ [M+NH$_4$]$^+$: 275.1139, found: 275.1139 (0.00 ppm)

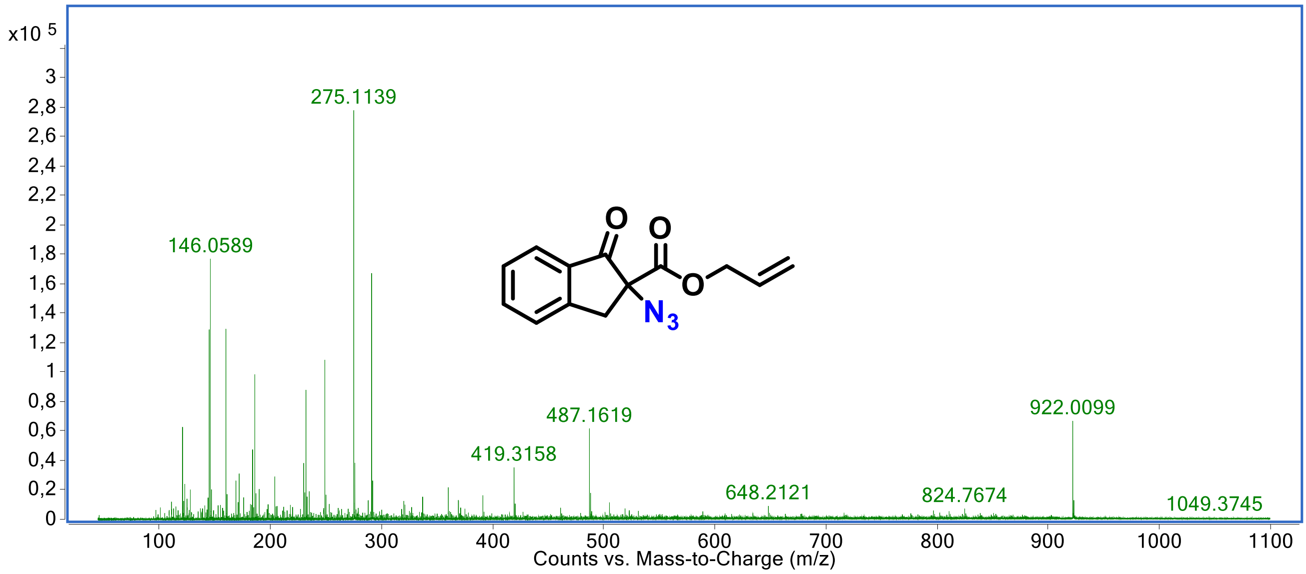

2d, HRMS (ESI$^+$-QqTOF, *m/z*):

calculated for $C_{17}H_{17}N_4O_3$ [M+NH$_4$]$^+$: 325.1295, found: 325.1295 (0.00 ppm)

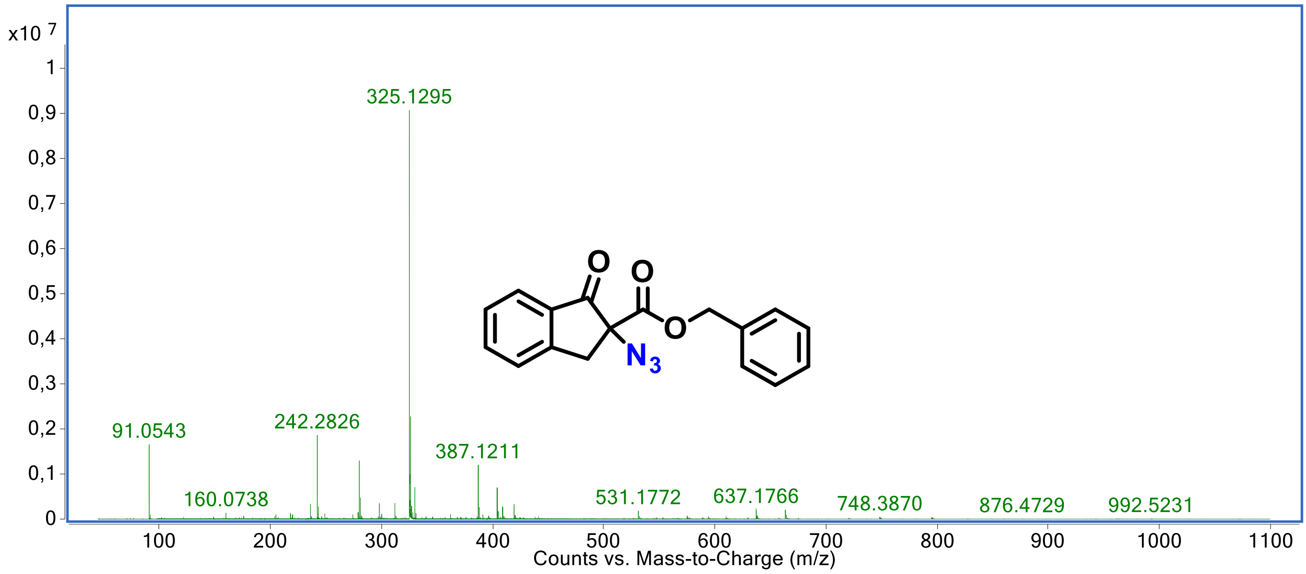

2e, IR HRMS (ESI$^+$-QqTOF, *m/z*):

calculated for $C_{20}H_{26}N_4O_3$ $[M+NH_4]^+$: 369.1921, found: 369.1922 (0.27 ppm)

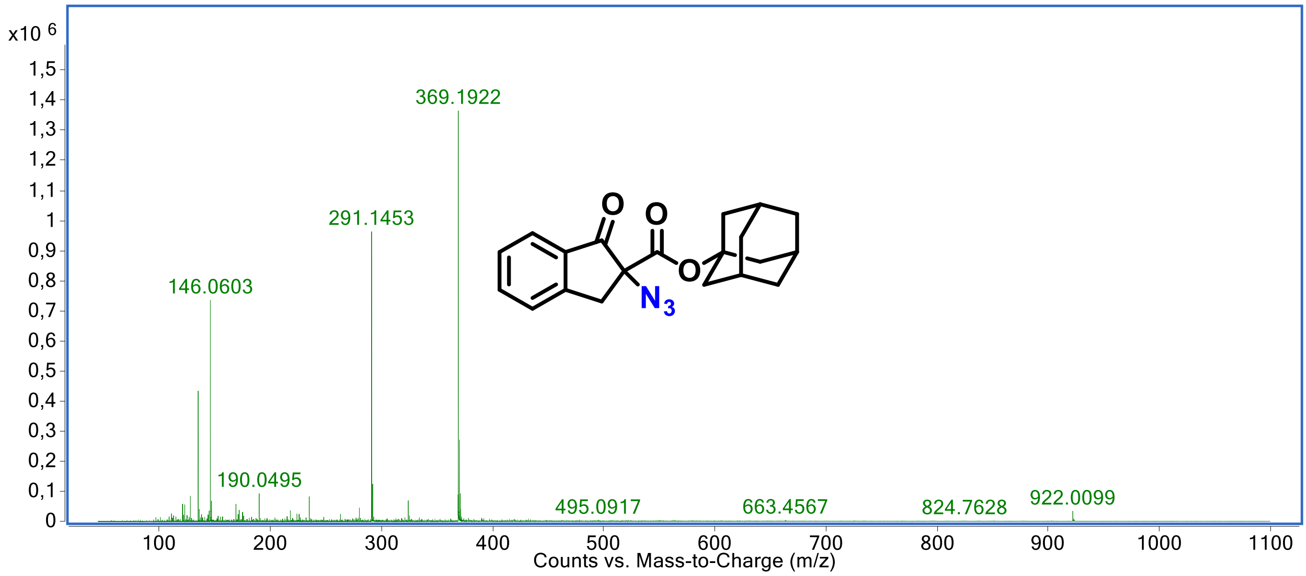

2f, HRMS (ESI$^+$-QqTOF, *m/z*):

calculated for $C_{19}H_{21}N_4O_3$ $[M+NH_4]^+$: 353.1608, found: 353.1608 (0.00 ppm)

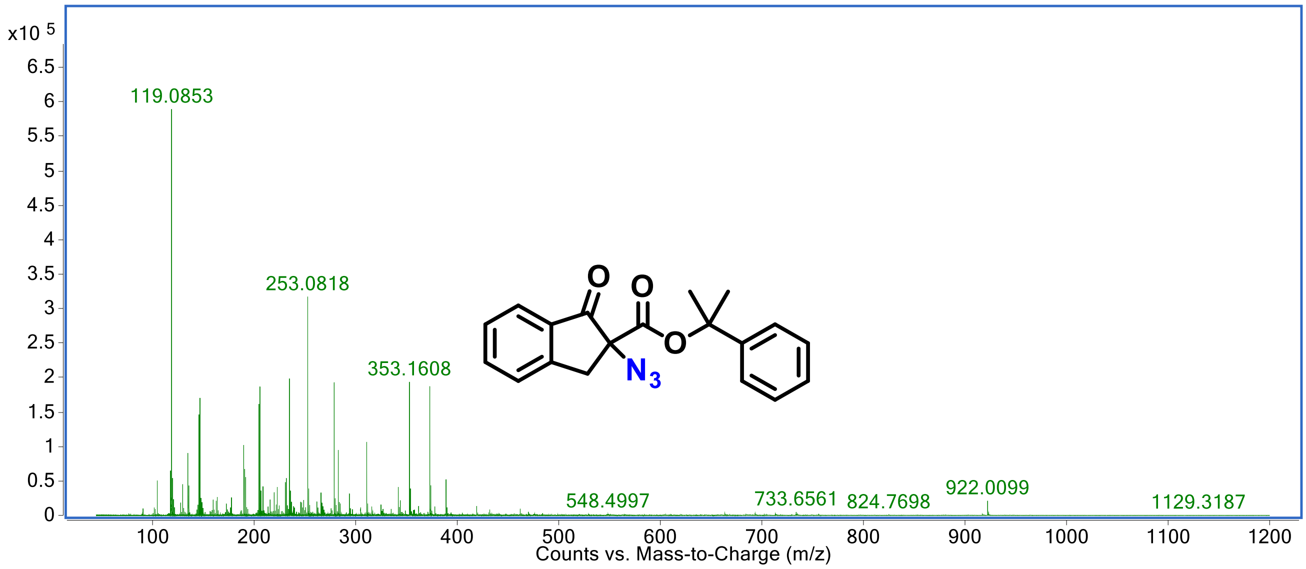

2g, HRMS (ESI$^+$-QqTOF, *m/z*):

calculated for $C_{14}H_{18}FN_4O_3$ [M+NH$_4$]$^+$: 309.1357, found: 309.1357 (0.00 ppm)

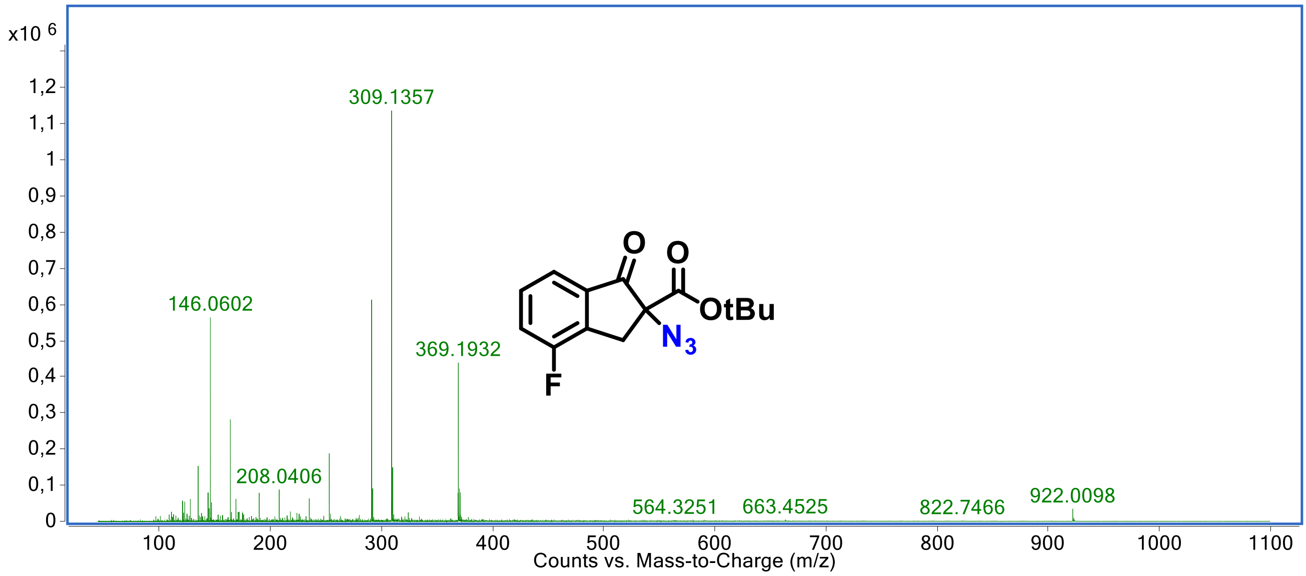

2h, HRMS (ESI$^+$-QqTOF, *m/z*):

calculated for $C_{15}H_{21}N_4O_3$ [M+NH$_4$]$^+$: 305.1608, found: 305.1608 (0.00 ppm)

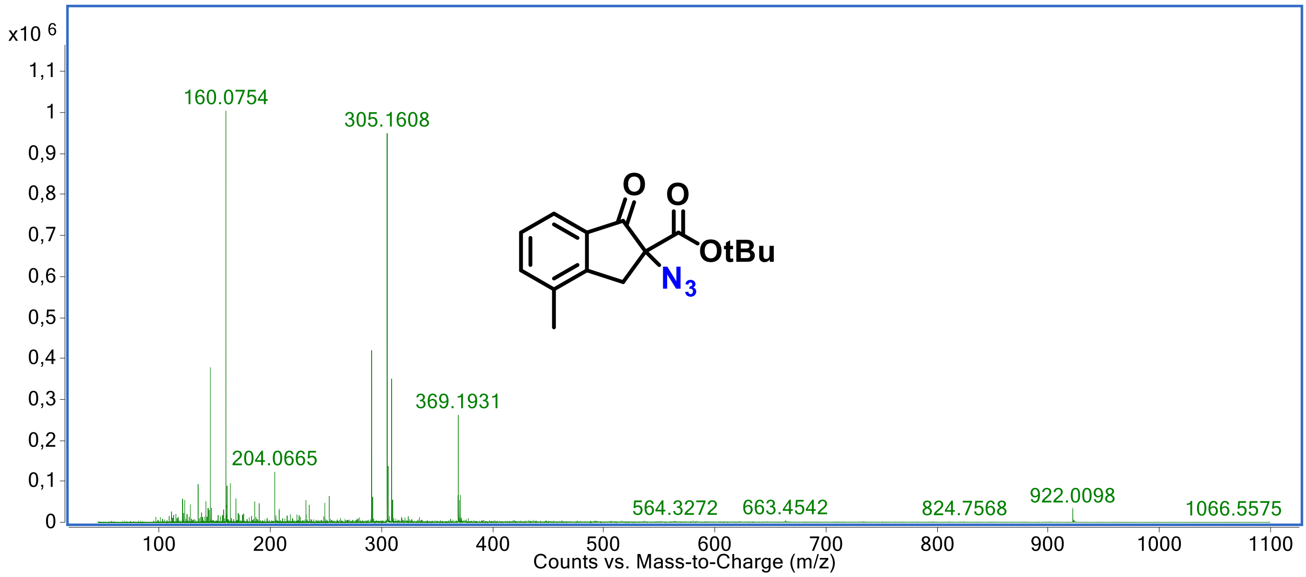

2i, HRMS (ESI$^+$-QqTOF, *m/z*):

calculated for $C_{14}H_{18}FN_4O_3$ $[M+NH_4]^+$: 309.1357, found: 309.1357 (0.00 ppm)

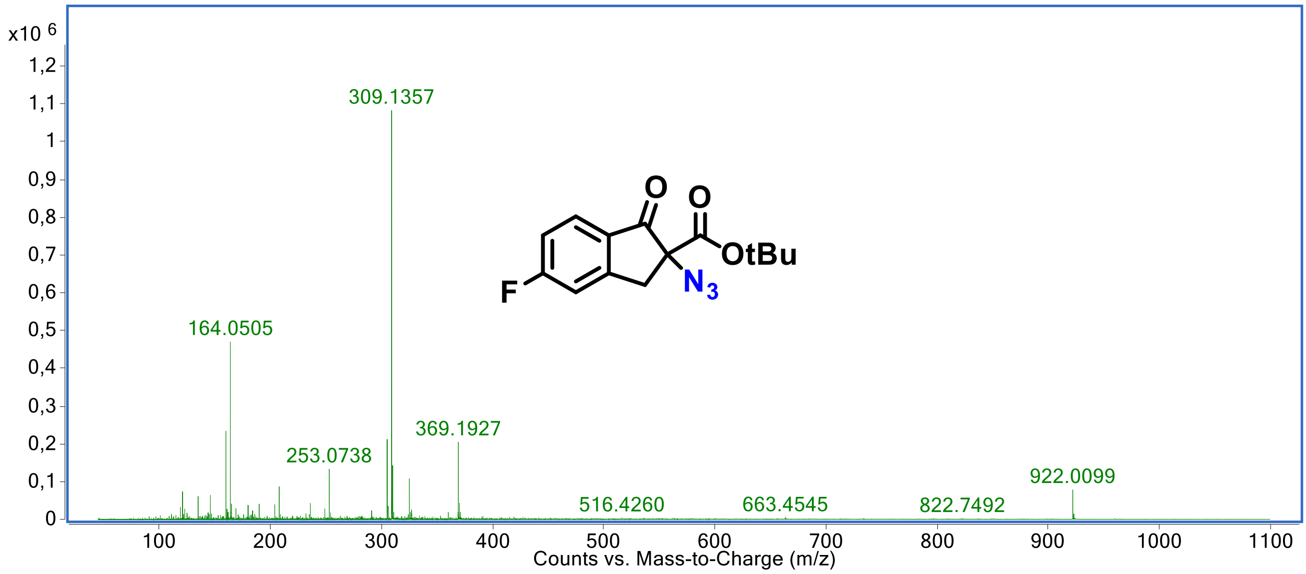

2j, HRMS (ESI$^+$-QqTOF, *m/z*):

calculated for $C_{14}H_{18}{}^{35}ClN_4O_3$ $[M+NH_4]^+$: 325.1062, found: 325.1061 (-0.31 ppm)

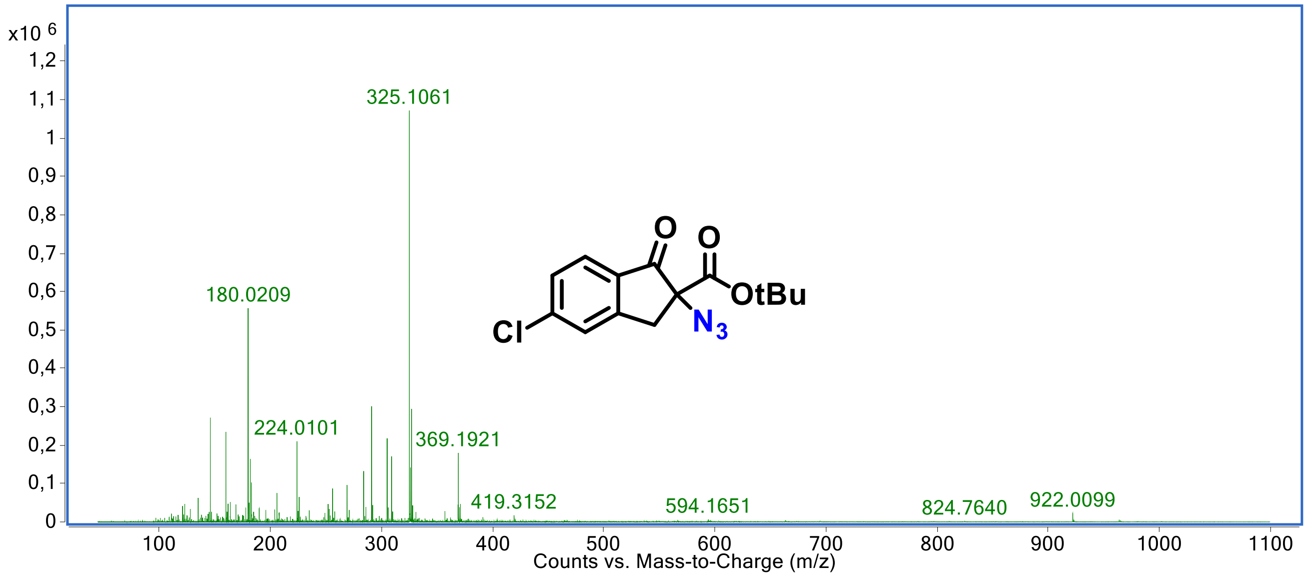

2k, HRMS (ESI$^+$-QqTOF, *m/z*):

calculated for $C_{14}H_{18}{}^{81}BrN_4O_3$ $[M+NH_4]^+$: 371.0538, found: 369.0538 (0.00 ppm)

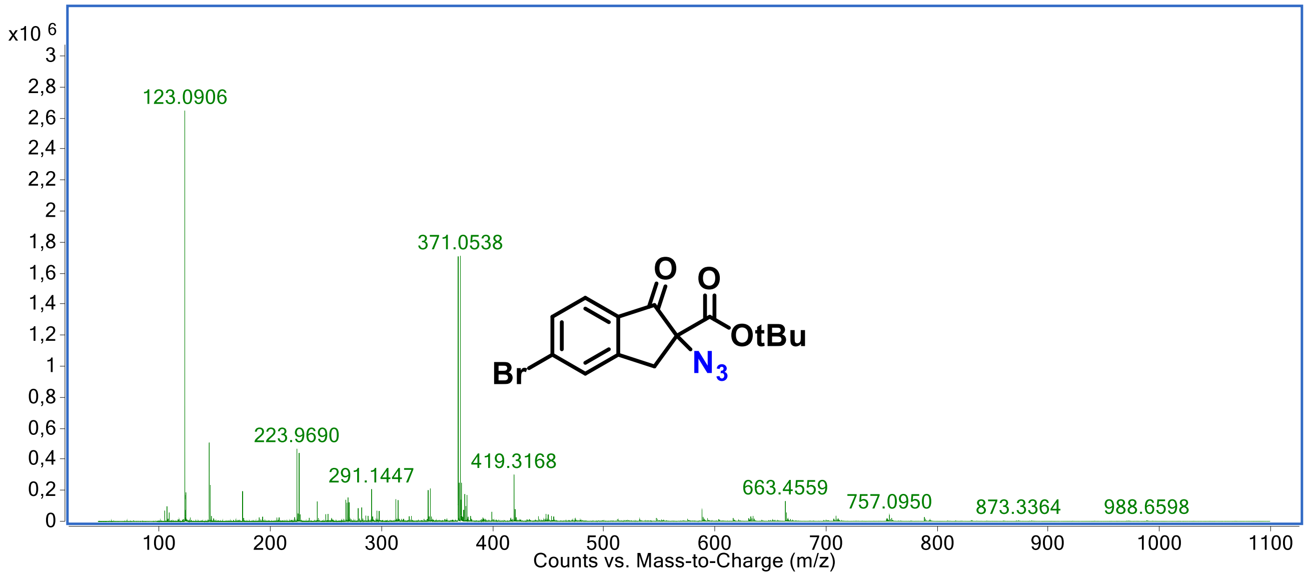

2l, HRMS (ESI$^+$-QqTOF, *m/z*):

calculated for $C_{15}H_{21}N_4O_4$ $[M+NH_4]^+$: 321.1557, found: 321.1557 (0.00 ppm)

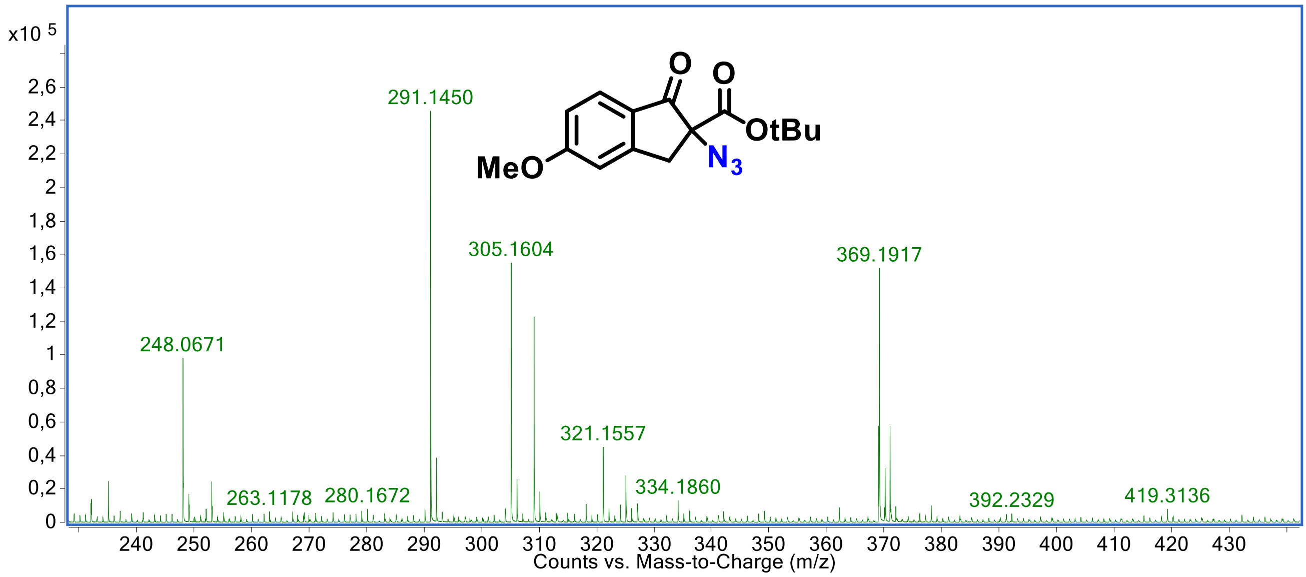

2m, HRMS (ESI$^+$-QqTOF, *m/z*):

calculated for $C_{15}H_{21}N_4O_4$ [M+NH$_4$]$^+$: 321.1557, found: 321.1557 (0.00 ppm)

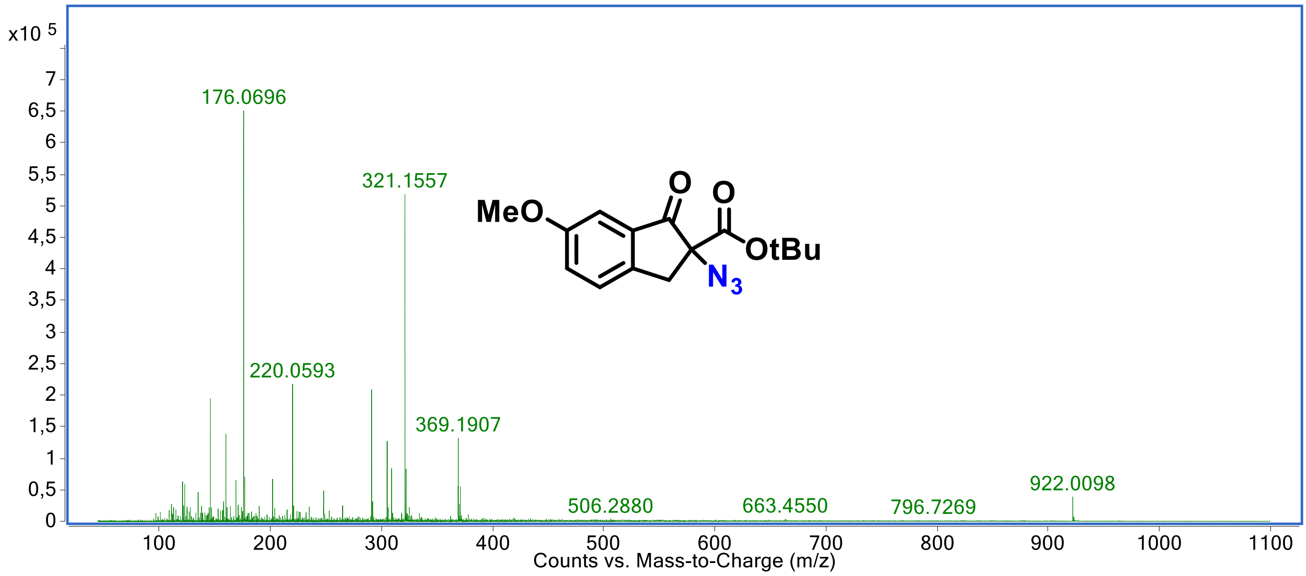

2n, HRMS (ESI$^+$-QqTOF, *m/z*):

calculated for $C_{15}H_{21}N_4O_3$ [M+NH$_4$]$^+$: 305.1608, found: 305.1608 (0.00 ppm)

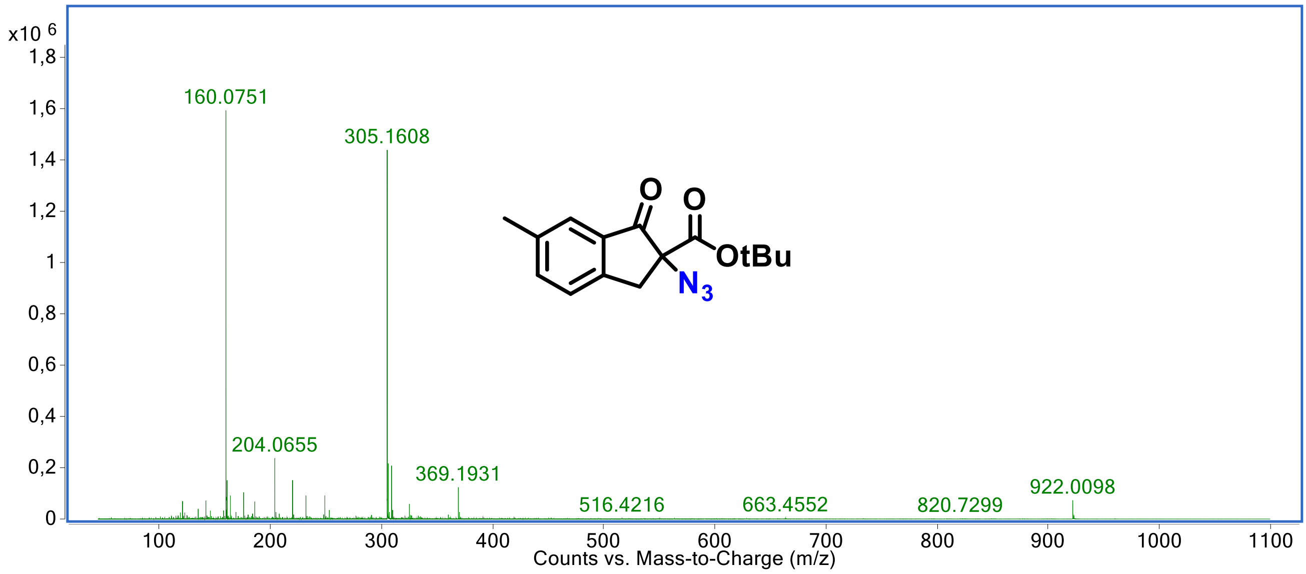

5a, HRMS (ESI$^+$-QqTOF, *m/z*):

calculated for $C_{11}H_{13}N_4O_2$ $[M+NH_4]^+$: 233.1033, found: 233.1033 (0.00 ppm)

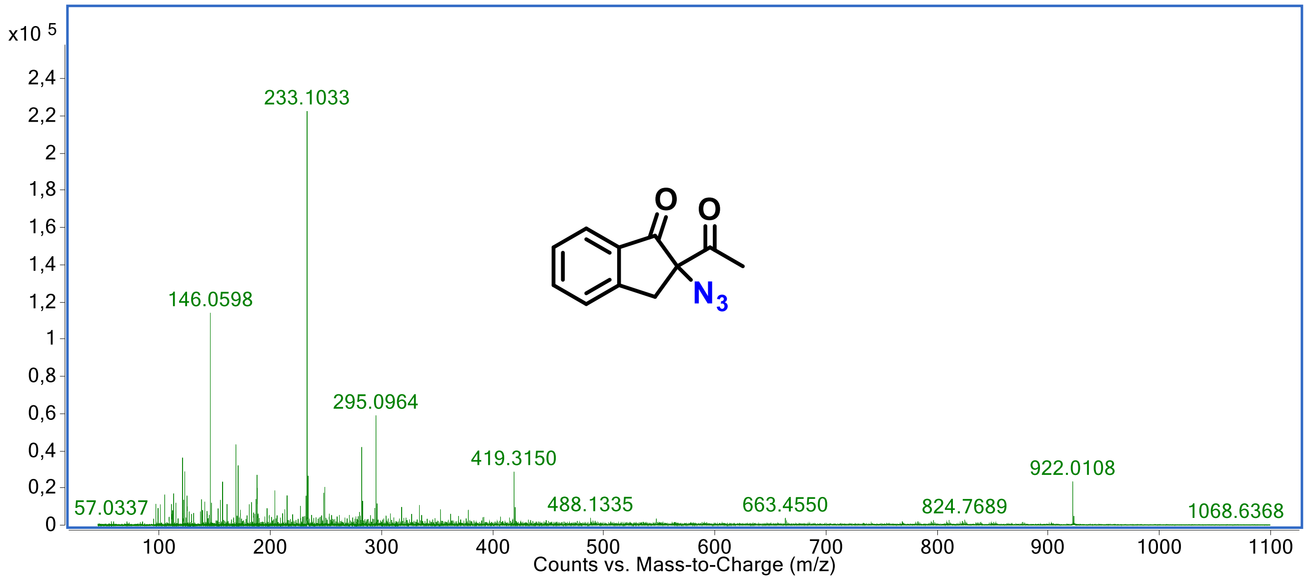

5b, HRMS (ESI$^+$-QqTOF, *m/z*):

calculated for $C_{16}H_{15}N_4O_2$ $[M+NH_4]^+$: 295.1190, found: 295.1190 (0.00 ppm)

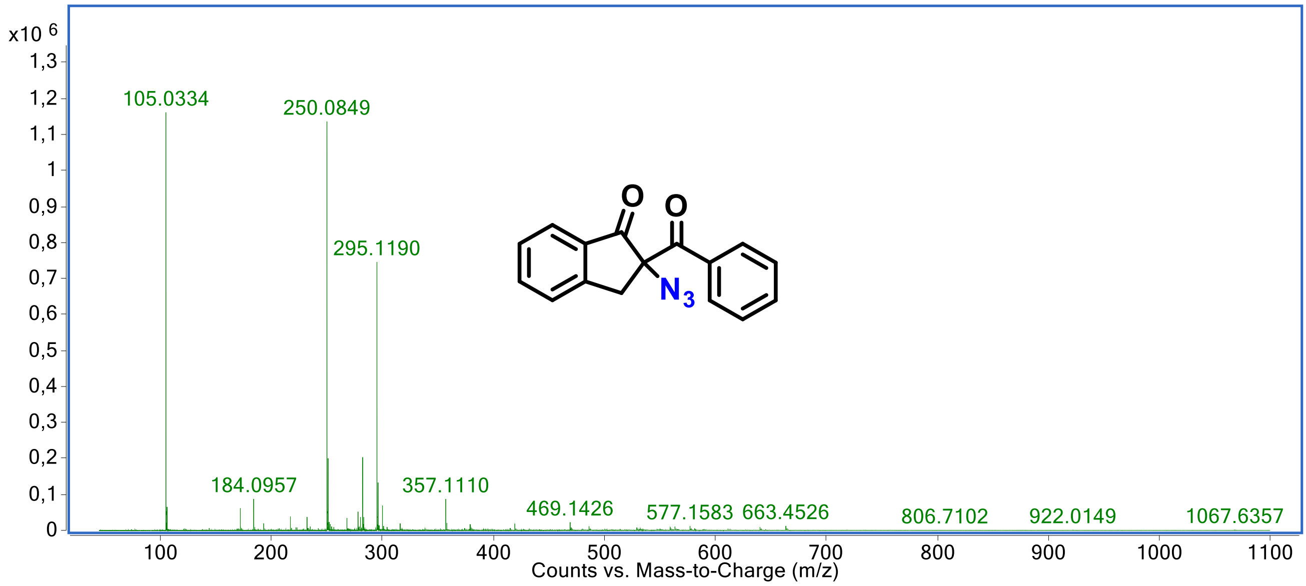

6, HRMS (ESI$^+$-QqTOF, *m/z*):

calculated for $C_{14}H_{15}N_4O_3$ [M+H]$^+$: 287.1139, found: 287.1139 (0.00 ppm)

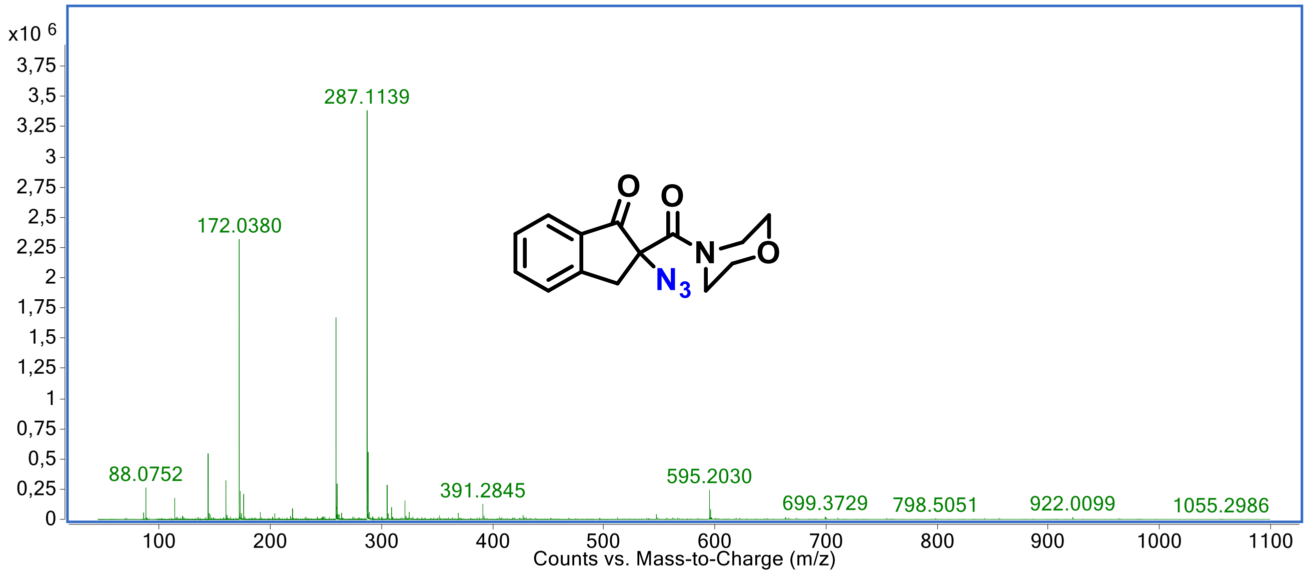

7a, HRMS (ESI$^+$-QqTOF, *m/z*):

calculated for $C_6H_8N_3O_4$ [M+NH$_4$]$^+$: 203.0775, found: 203.0776 (0.49 ppm)

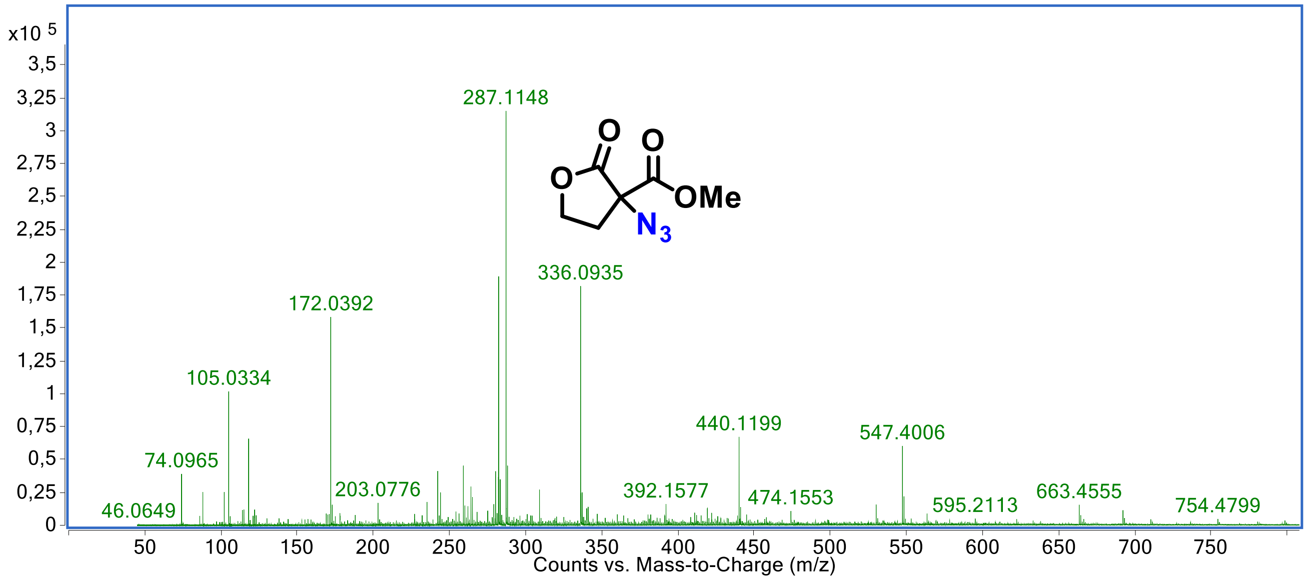

7b, HRMS (ESI$^+$-QqTOF, *m/z*):

calculated for $C_8H_{13}N_4O_4$ [M+NH$_4$]$^+$: 229.0931, found: 229.0931 (0.00 ppm)

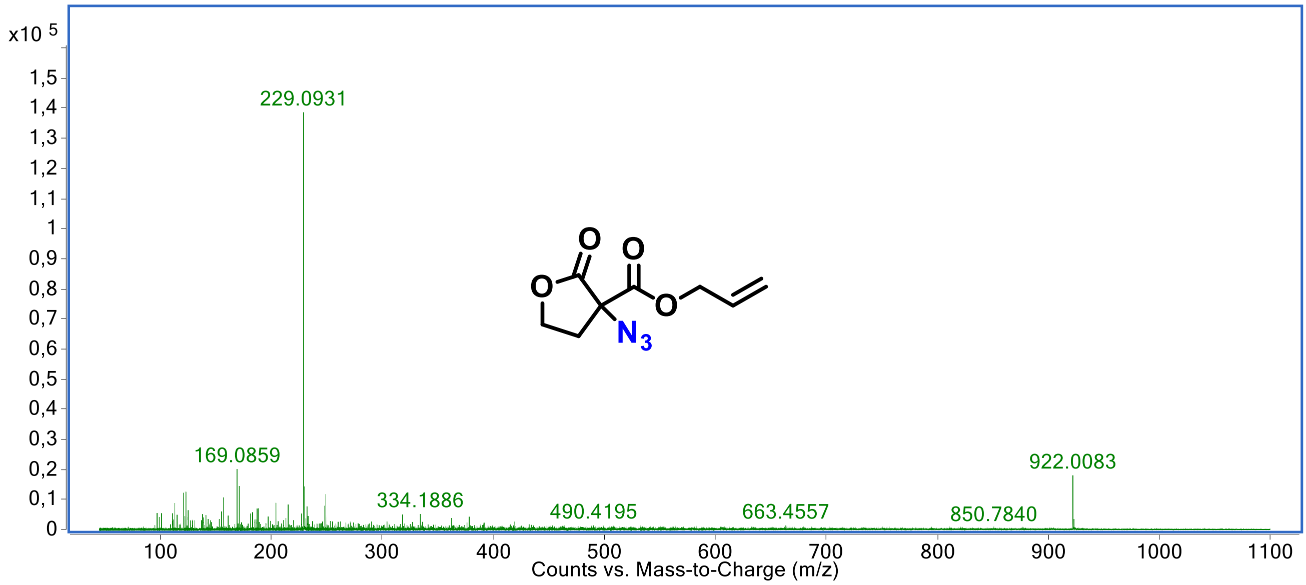

7c, HRMS (ESI$^+$-QqTOF, *m/z*):

calculated for $C_{11}H_{13}N_4O_3$ [M+NH$_4$]$^+$: 249.0982, found: 287.0982 (0.00 ppm)

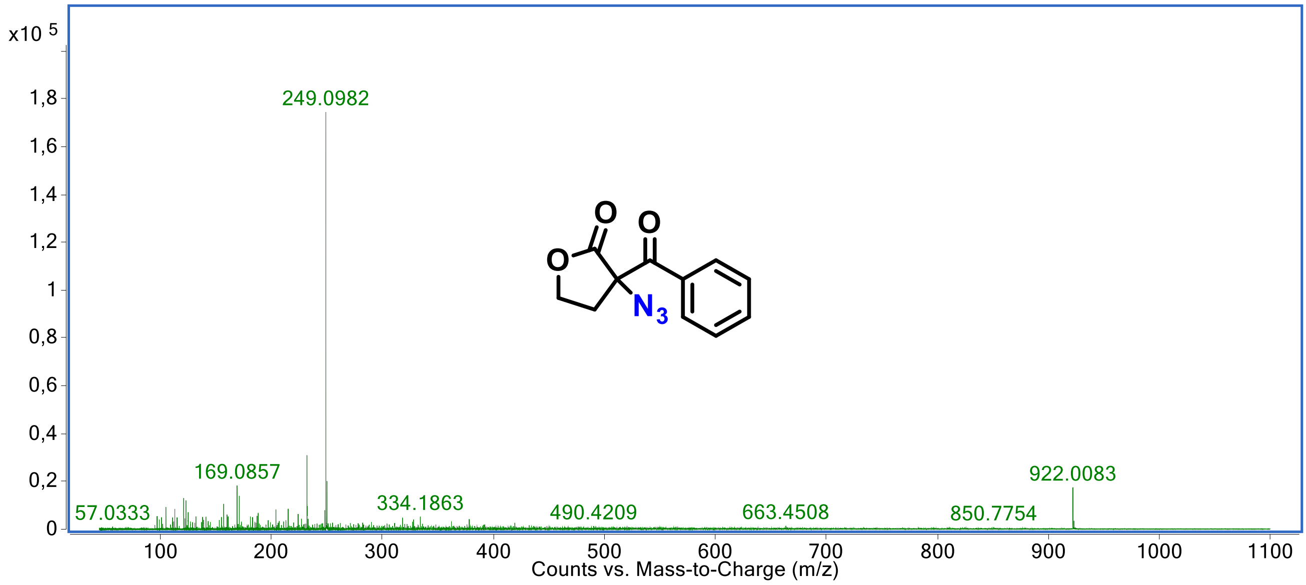

10a, HRMS (ESI$^+$-QqTOF, *m/z*):

calculated for $C_{14}H_{19}N_2O_5$ $[M+NH_4]^+$: 295.1288, found: 295.1288 (0.00 ppm)

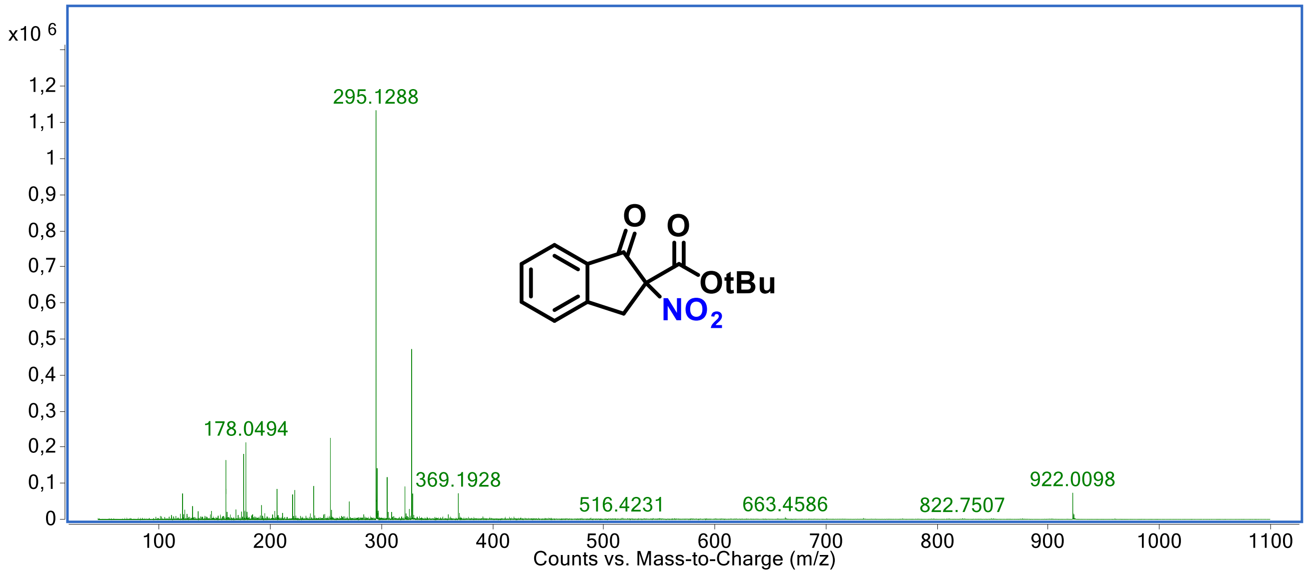

10b, HRMS (ESI$^+$-QqTOF, *m/z*):

calculated for $C_{11}H_{13}N_2O_5$ $[M+NH_4]^+$: 253.0820, found: 253.0820 (0.00 ppm)

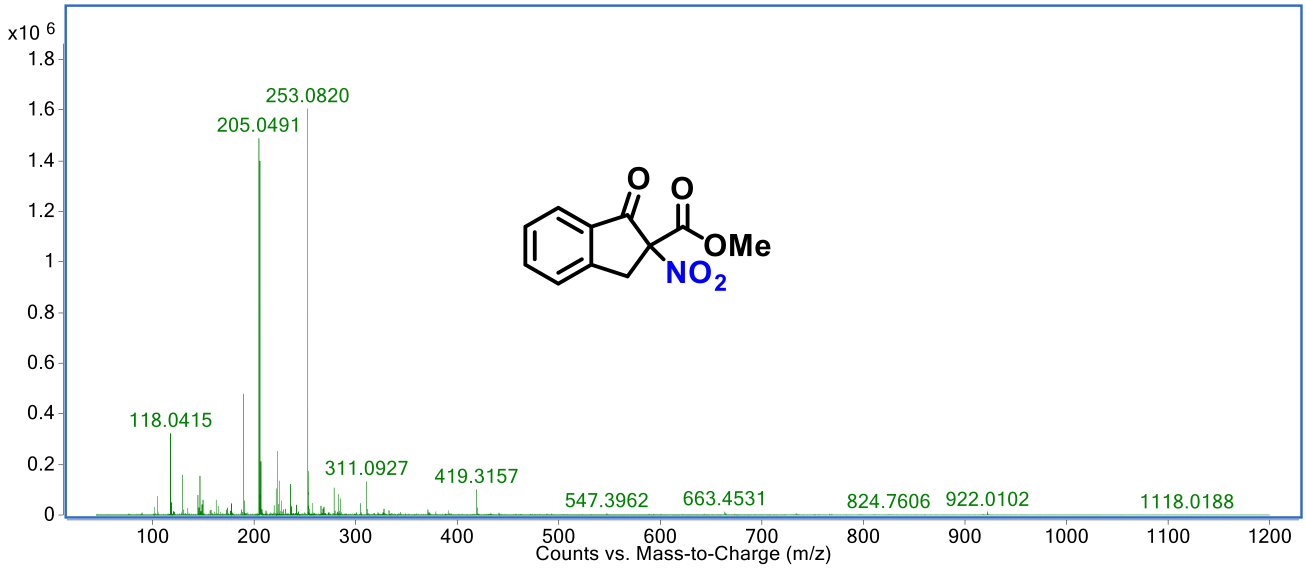

10c, HRMS (ESI$^+$-QqTOF, *m/z*):

calculated for $C_{13}H_{15}N_2O_5$ $[M+NH_4]^+$: 279.0975, found: 279.0975 (0.00 ppm)

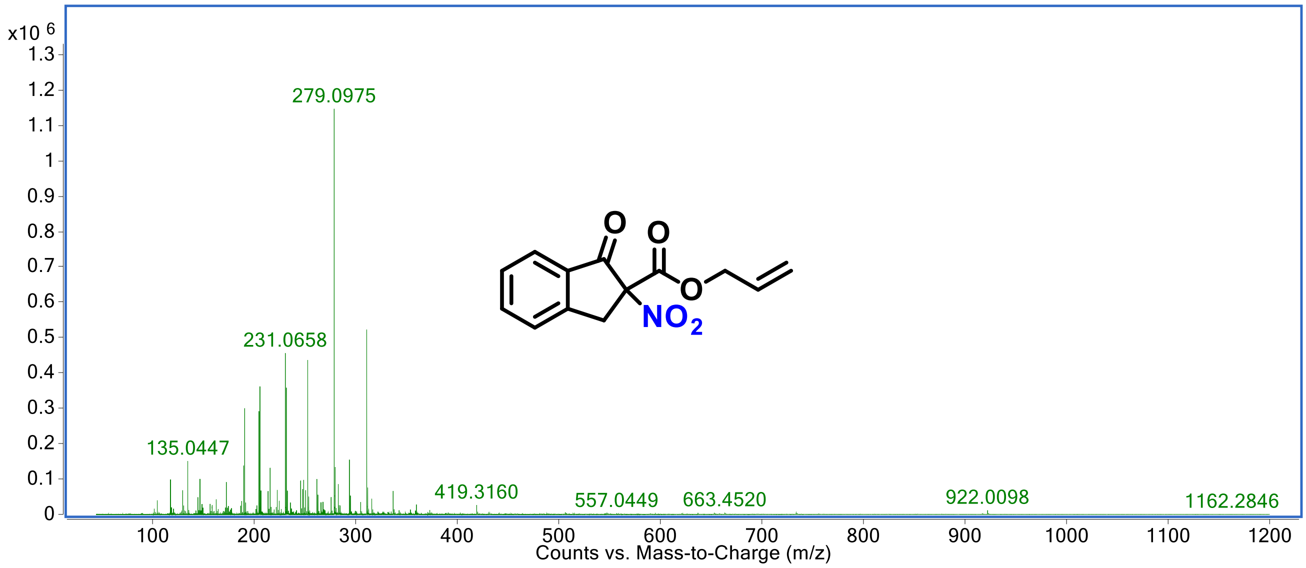

9. References

[1] Lifchits, O.; Demoulin, N.; and List, B.; Direct Asymmetric α-Benzoyloxylation of Cyclic Ketones; *Angew. Chem. Int. Ed.* **2011**, 50, 9680-9683.

[2] (a) Bräse, S. and Banert, K. *Organic Azides*: *Syntheses and Applications*; Wiley, 2010. DOI: 10.1002/9780470682517. (b) Bräse, S.; Gil, C.; Knepper, K. and Zimmermann, V.; Organic azides: an exploding diversity of a unique class of compounds; *Angew. Chem. Int. Ed* **2005**, 44, 5188-5240. (c) Treitler, D. S. and Leung, S. How Dangerous Is Too Dangerous? A Perspective on Azide Chemistry. *J. Org. Chem*. **2022**, 87, 11293-11295. (d) Kolb, H. C.; Finn, M. G. and Sharpless, K. B.; Click Chemistry: Diverse Chemical Function from a Few Good Reactions; *Angew. Chem. Int. Ed.* **2001**, 40, 2004-2021. (e) Wiss, J.; Fleury, C.; Heuberger, C.; Onken, U.and Glor, M.; Explosion and Decomposition Characteristics of Hydrazoic Acid in the Gas Phase; *Org. Process Res. Dev.* **2007**, 11, 1096-1103. (f) Kopach, M. E.; Murray, M. M.; Braden, T. M.; Kobierski, M. E. and Williams, O. L.; Improved Synthesis of 1-(Azidomethyl)-3,5-bis-(trifluoromethyl)benzene: Development of Batch and Microflow Azide Processes; *Org. Process Res. Dev.* **2009**, 13, 152-160. (g) Bretherick, L. *Handbook of Reactive Chemical Hazards,* 4th ed.; Butterworths: London, 1990; p 1360.

[3] Moss, T. A.; Fenwick, D. R. and Dixon, D. J.; Enantio- and Diastereoselective Catalytic Alkylation Reactions with Aziridines; *J. Am. Chem. Soc.* **2008**, 130, 10076-10077.

[4] Y.-N. Duan, L.-Q. Cui, L.-H. Zuo and C. Zhang; Recyclable Hypervalent-Iodine-Mediated Dehydrogenative α,*β*'-Bifunctionalization of *β*-Keto Esters Under Metal-Free Conditions; *Chem. Eur. J.* **2015**, 21, 13052-13057.

[5] Vita, M. V. and Waser, J.; Azidation of *β*-Keto Esters and Silyl Enol Ethers with α-Benziodoxole Reagent; *Org. Lett.* **2013**, 15, 3246-3249.

[6] Craig, R. A.; Loskot, S. A.; Mohr, J. T.; Behenna, D. C.; Harned, A. M. and Stoltz, B. M.; Palladium-Catalyzed Enantioselective Decarboxylative Allylic Alkylation of Cyclopentanones; *Org. Lett.* **2015**, 17, 5160-5163.

[7] (a) Christoffers, J. and Önal, N., Azeotropic Transesterification of *β*-Keto Esters. *Eur. J. Org. Chem.* **2000**, 1633-1635. (b) Baumhof, P.; Mazitschek, R. and Giannis, A., A Mild and Effective Method for the Transesterification of Carboxylic Acid Esters; *Angew. Chem. Int. Ed.* **2001**, 40, 3672-3674. (c) Lian, M.; Li, Z.; Du, J.; Meng, Q. and Gao, Z. Asymmetric Direct α-Hydroxylation of *β*-Oxo Esters by Phase-Transfer Catalysis Using Chiral Quaternary Ammonium Salts. *Eur. J. Org. Chem.* **2010**, 34, 6525-6530. (d) Pericas, À.; Shafir, A. and Vallribera, A. Zinc(II) oxide: An efficient catalyst for selective transesterification of *β*-ketoesters; *Tetrahedron* **2008**, 64, 9258-9263.

[8] Li, M.; Hawkins, A.; Barber, D. M.; Bultinck, P.; Herrebout, W. and Dixon, D. J.; Enantio- and diastereoselective palladium catalysed arylative and vinylative allene carbocyclisation cascades; *Chem. Commun.* **2013**, 49, 5265-5267.

[9] Q. Tian, Z. Gan, X. Wang, D. Li, W. Luo, H. Wang, Z. Dai and J. Yuan; Imidazolium Chloride: An Efficient Catalyst for Transamidation of Primary Amines; *Molecules* **2018**, 2234-2237.

[10] L. Cicco, V. Addante, A. Temperini, C. A. Donau, K. Karaghiosoff, F. M. Perna and V. Capriati; Toward Customized Tetrahydropyran Derivatives through Regioselective *α*-Lithiation and Functionalization of 2-Phenyltetrahydropyran; *Eur. J. Org. Chem.* **2016**, 19, 3157-3161.

[11] H. Kawada, H. Ebiike, M. Tsukazaki, M. Nakamura, K. Morikami, K. Yoshinari, M. Yoshida, K. Ogawa, N. Shimma, T. Tsukuda and J. Ohwada; Lead optimization of a dihydropyrrolopyrimidine inhibitor against phosphoinositide 3-kinase (PI3K) to improve the phenol glucuronic acid conjugation; *Bioorg. Med. Chem. Lett.* **2013**, 23, 673-678.

[12] T. Ooi, T. Miki, K. Fukumoto and K. Maruoka; Asymmetric Synthesis of α-Acyl-γ-butyrolactones Possessing All-Carbon Quaternary Stereocenters by Phase-Transfer-Catalyzed Alkylation; *Adv. Synth. Catal.* **2006**, 348, 1539-1542.

[13] M. Tiffner, L. Stockhammer, J. Schörgenhumer, K. Röser and M. Waser; Towards an Asymmetric Organocatalytic α-Azidation of *β*-Ketoesters; *Molecules* **2018**, 1142-1151.

[14] M. Uyanik, N. Sahara, M. Tsukahara, Y. Hattori and K. Ishihara; Chemo- and Enantioselective Oxidative *α*-Azidation of Carbonyl Compounds; *Angew. Chem. Int. Ed.* **2020**, 59, 17110–17117.

[15] Q.-H. Deng, T. Bleith, H. Wadepohl, and L. H. Gade; Enantioselective Iron-Catalyzed Azidation of *β*-Keto Esters and Oxindoles; *J. Am. Chem. Soc.* **2013**, 135, 5356–5359.

[16] M. V. Vita and J. Waser; Azidation of β-Keto Esters and Silyl Enol Ethers with a Benziodoxole Reagent; *Org. Lett.* **2013**, 15, 3246-3249.

[17] K. Shibatomi, Y. Soga, A. Narayama, I. Fujisawa, and S. Iwasa; Highly Enantioselective Chlorination of *β*-Ketoesters and Subsequent S_N2 Displacement of Tertiary Chlorides: A Flexible Method for the Construction of Quaternary Stereogenic Centers; *J. Am. Chem. Soc.* **2012**, 134, 9836–9839.

[18] T. Harschneck, S. Hummel, S. F. Kirsch, and P. Klahn; Practical Azidation of 1,3-Dicarbonyls; *Chem. Eur. J.* **2012**, 18, 1187-1193.

[19] F.-Z. Han, L.-L. Li, L.-N. Jia, X.-P. Hu; Catalyst-free nitration of the aliphatic CAH bonds of tertiary *β*-keto esters with tert-butyl nitrite: Access to *α*-quaternary *α*-amino acid precursors; *Tetrahedron Lett.* **2022**, 99, 153844-153849.
